# Protein signature of human skin fibroblasts allows the study of the molecular etiology of rare neurological diseases

**DOI:** 10.1186/s13023-020-01669-1

**Published:** 2021-02-09

**Authors:** Andreas Hentschel, Artur Czech, Ute Münchberg, Erik Freier, Ulrike Schara-Schmidt, Albert Sickmann, Jens Reimann, Andreas Roos

**Affiliations:** 1 Leibniz-Institut für Analytische Wissenschaften - ISAS - e.V, Dortmund, Germany; 2 Department of Pediatric Neurology, Faculty of Medicine, University Hospital Essen, University of Duisburg-Essen, Essen, Germany; 3 Muscle Laboratory, Department of Neurology, University of Bonn, Medical Centre, Bonn, Germany; 4 Children’s Hospital of Eastern Ontario Research Institute, Ottawa, ON Canada

**Keywords:** Allgrove syndrome, Aladin, AAAS, Triple-A syndrome, Myopodin/synaptopodin-2, Ataxin-2

## Abstract

**Background:**

The elucidation of pathomechanisms leading to the manifestation of rare (genetically caused) neurological diseases including neuromuscular diseases (NMD) represents an important step toward the understanding of the genesis of the respective disease and might help to define starting points for (new) therapeutic intervention concepts. However, these “discovery studies” are often limited by the availability of human biomaterial. Moreover, given that results of next-generation-sequencing approaches frequently result in the identification of ambiguous variants, testing of their pathogenicity is crucial but also depending on patient-derived material.

**Methods:**

Human skin fibroblasts were used to generate a spectral library using pH8-fractionation of followed by nano LC-MS/MS. Afterwards, Allgrove-patient derived fibroblasts were subjected to a data independent acquisition approach. In addition, proteomic signature of an enriched nuclear protein fraction was studied. Proteomic findings were confirmed by immunofluorescence in a muscle biopsy derived from the same patient and cellular lipid homeostasis in the cause of Allgrove syndrome was analysed by fluorescence (BODIPY-staining) and coherent anti-Stokes Raman scattering (CARS) microscopy.

**Results:**

To systematically address the question if human skin fibroblasts might serve as valuable biomaterial for (molecular) studies of NMD, we generated a protein library cataloguing 8280 proteins including a variety of such linked to genetic forms of motoneuron diseases, congenital myasthenic syndromes, neuropathies and muscle disorders. In silico-based pathway analyses revealed expression of a diversity of proteins involved in muscle contraction and such decisive for neuronal function and maintenance suggesting the suitability of human skin fibroblasts to study the etiology of NMD. Based on these findings, next we aimed to further demonstrate the suitability of this in vitro model to study NMD by a use case: the proteomic signature of fibroblasts derived from an Allgrove-patient was studied. Dysregulation of paradigmatic proteins could be confirmed in muscle biopsy of the patient and protein-functions could be linked to neurological symptoms known for this disease. Moreover, proteomic investigation of nuclear protein composition allowed the identification of protein-dysregulations according with structural perturbations observed in the muscle biopsy. BODIPY-staining on fibroblasts and CARS microscopy on muscle biopsy suggest altered lipid storage as part of the underlying disease etiology.

**Conclusions:**

Our combined data reveal that human fibroblasts may serve as an in vitro system to study the molecular etiology of rare neurological diseases exemplified on Allgrove syndrome in an unbiased fashion.

## Introduction

A considerable number of patients suffer from diseases affecting the proper function of the nervous system or skeletal muscle or even both. Although these diseases are individually considered as rare, they nevertheless cause a significant burden for the patients and their families. Along the neuronal axis, prominent examples of these disorders are genetically-caused forms of brain malformations (as a very heterogeneous disease entity), motoneuron diseases such as 5q-associated spinal muscular atrophy and familiar amyotrophic lateral sclerosis, neuropathies/peripheral nerve diseases and disorders affecting the nerve terminals (congenital myasthenic syndromes; CMS) as well as myopathic disorders. Often, these diseases have both a wide clinical phenotype and partially overlapping manifestations suggesting the presence of common pathophysiological cascades. Together, this group of diseases affects a large fraction (approximately 3–5%) of all children, usually at early age and often leading to premature death or chronic disability. Despite the discovery of many genes—especially in the next-generation-sequencing era—causative for different forms of neurological diseases, in recent years, limited progress has been achieved in the therapeutic intervention of these diseases which is not based on gene-replacement. In addition, also for neuromuscular diseases (NMDs) treatable by gene replacement, additional therapeutic concepts targeting pathological alterations which are not reversed by gene/protein-restoration (such as altered signaling modules) are demanded [1]. One strategy to overcome this problem might be the classification of disease groups based on the main underlying pathomechanisms leading toward the definition of common treatment-concepts [2]. However, doing so, a profound understanding of these mechanisms is needed. Clinical proteomics has gained enormous popularity over the last decade finding increasing application in the elucidation of neurological diseases [3], albeit with a main focus on common neurological disorders such as dementia and Parkinson’s disease [4] as these represent a real challenge for the health care system and aging. Often, biochemical and functional studies of neurological disorders are “limited” to animal models. This is caused by a lack of availability of human material, especially in vitro models. However, different clinical proteomic studies toward a better understanding of the underlying pathophysiology of rare forms of inherited neurological diseases have been carried out on patient-derived skin fibroblasts. Results of these studies provided significant insights into cellular mechanisms leading to phenotypical manifestation [5, 6] suggesting that fibroblasts might serve as a valuable in vitro model for molecular studies of these diseases. To systematically address this hypothesis, in this study, a protein/spectral library of human skin fibroblasts was generated and in silico-based pathway analyses were performed to highlight the abundance of proteins crucial of neuronal development, maintenance and function. Moreover, an alignment of our library with proteins known to be responsible for genetic forms of NMDs revealed the expression of a magnitude of such linked to motoneuron diseases, congenital myasthenic syndromes, neuropathies/peripheral nervous system disease and muscle disorders. Using this library as the basis for data independent acquisition approaches (DIA), an exemplary rare inherited neurological disease, Allgrove syndrome (MIM: 231550), was studied. This rare disorder, also known as triple-A syndrome, is characterized by achalasia, alacrimia and adrenocorticotropic hormone (ACTH)-resistant adrenal cortex insufficiency complicated by neurological abnormalities. The disease is caused by autosomal recessive mutations within the *AAAS* gene, which encodes a nucleoporin called ALADIN [7, 8].

Proteomic findings on fibroblasts and results of validation studies on a muscle biopsy (c.762delC mutation within the *AAAS* gene) provided significant molecular insights into underlying molecular etiology and hereby added perturbed lipid homeostasis to the list of pathophysiological processes. Hence, our combined data support the concept of fibroblasts serving as a valuable model to study the molecular genesis of neurological diseases as exemplified here for Allgrove syndrome.

## Results

### Protein cataloguing in human fibroblasts

**Fig. 1:**
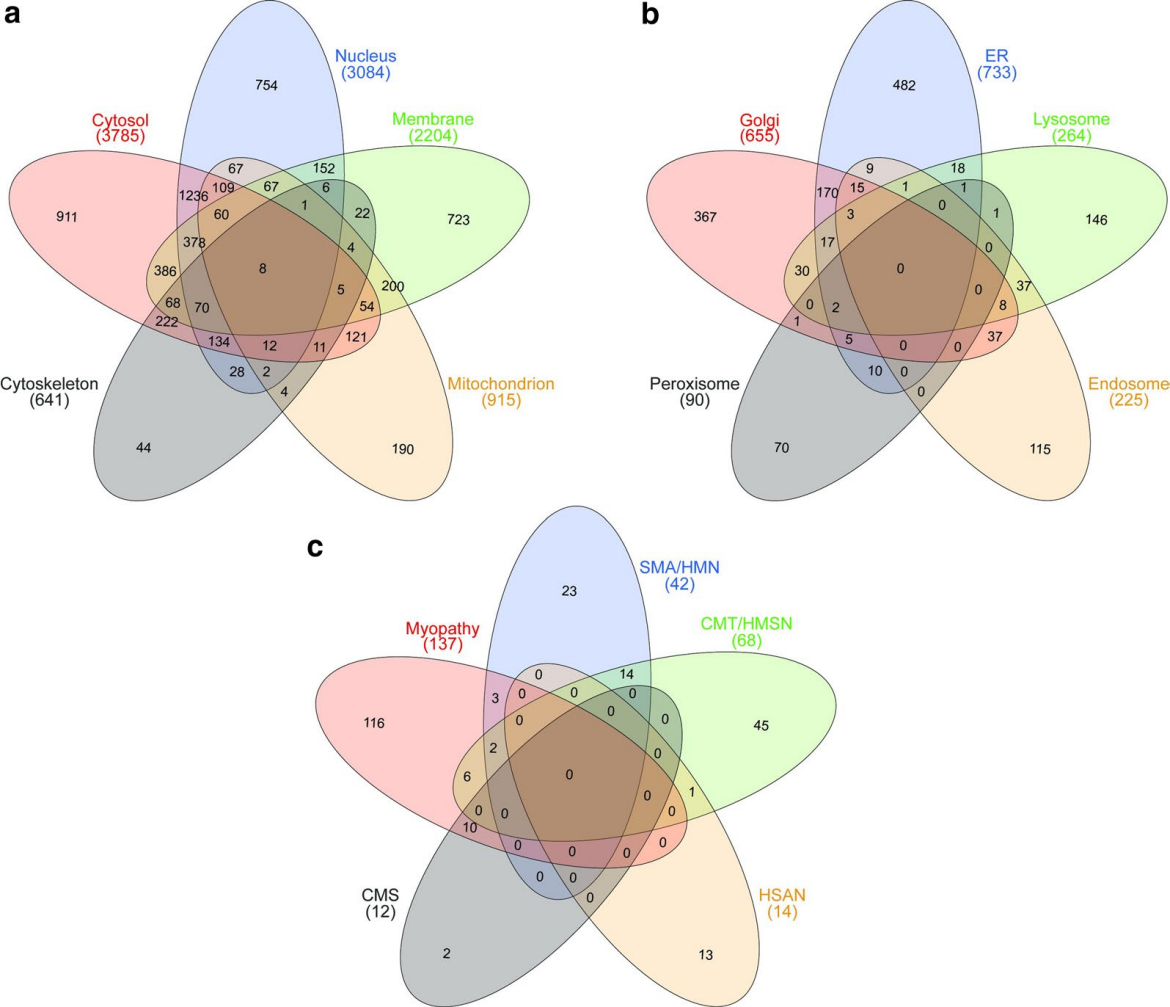
Overview of subcellular compartments (**a**, **b**) and NMDs (**c**) covered by the created fibroblast library: **a**, **b** Presentation of the different subcellular fibroblast compartments including the corresponding number of proteins identified to be localized to these respective compartments. While some proteins are exclusively present in one compartment, a considerable number of such localized to different/multiple compartments is covered in the library. Proteins listed for the subcellular compartments illustrated in **b** might be also covered in **a** as being resident to membranes or the cytosol. **c** Illustrates the distribution of proteins identified for certain NMDs along the neuromuscular axis including motoneuron and peripheral nervous system diseases, defects in neuromuscular transmission and muscular disorders

To investigate if human skin fibroblasts may serve as suitable in vitro models to study the molecular etiology of neurological diseases such as neuromuscular disorders and may thus help to overcome a limitation of human material enabling these studies, we created and analysed a protein/spectral library: after pH8-based fractionation of tryptic peptides obtained from whole protein extracts of fibroblasts, comprising patient and control samples, followed by LC-MS/MS-based protein identification, we generated a database of 8280 proteins (referring to a total number of 96,512 peptides) covering a total range of 5 orders of magnitude. Hereby, Vimentin (P08670; a class-III intermediate filament attached to the nucleus, endoplasmic reticulum and mitochondria) was identified as the highest abundant and Kallikrein-7 (P49862; degradation-catalyst of intercellular cohesive structures in the cornified skin layer) as the lowest abundant protein (Additional file 1: Table 1). These 8280 proteins cover all subcellular compartments (Fig. 1a, b). For the cytosol and the nucleus (including the nucleoplasm) 911 and 754 proteins are annotated to be exclusively present in these subcellular structures, respectively (Fig. 1a). The number of identified membrane proteins also include such localizing to the endoplasmic reticulum membrane, mitochondrial membrane, Golgi membrane and nuclear membrane, with 723 proteins only localized to one subcellular compartment. In addition, our library covers 190 proteins known to be only present in mitochondria and 44 proteins exclusively belonging to the cytoskeleton (Fig. 1a).

To evaluate the proteomic signature of human skin fibroblasts for potential analyses of genetically caused NMD, catalogued proteins were analysed: we filtered for proteins where defects in the corresponding genes were linked to the manifestation of diseases along the neuromuscular axis including motoneuron and peripheral nervous system diseases, defects in neuromuscular transmission and muscle disorders (https://neuromuscular.wustl.edu/) [9]. This approach revealed the expression of a total of 257 from 385 proteins (66.8%) linked to the manifestation of a respective disorder: for hereditary motoneuron disorders (consisting of spinal muscular atrophy (SMA), distal SMA and hereditary motoneuropathies (HMN)), 42 out of a total of 53 registered proteins (80.8%) were identified. For hereditary motor sensory neuropathies (HMSN), comprising a list of 91 known genes/proteins, 68 (74.7%) were identified. In addition, 14 out of 26 known genes/proteins (46.2%) causative for hereditary sensory and autonomic neuropathies (HSAN) were detected in skin fibroblasts. For congenital myasthenic syndrome (CMS) 12 of the currently known causative 35 genes/proteins (34.3%) were identified in our protein/spectral library. In addition, from a further list of 351 proteins related to muscle diseases, comprising not only such based on the above-mentioned sources but also candidates based on experimental findings for different muscle diseases, 137 proteins (39.0%) could be identified. These proteins are listed in Additional file 2: Table 2 and the distribution is illustrated in Fig. 1c. The spectral library data have been deposited to the ProteomeXchange Consortium via the PRIDE partner repository with the dataset identifier PXD019060.

### Global proteomic profiling of Allgrove-patient derived fibroblasts

After demonstration of the expression of a variety of proteins of neuromuscular significance in human skin fibroblasts, we next aimed to systematically address the suitability of these cells to study the molecular genesis of neuromuscular diseases and thus investigated the proteomic signature of fibroblasts derived from a patient suffering from Allgrove syndrome (see above) with pronounced neuromuscular symptoms [10]. Our approach allowed the quantification of 4817 proteins out of which 228 (4.73%) were significantly increased and 156 (3.24%) significantly decreased (Additional file 3: Table 3). The proteomic profiling data have been deposited to the ProteomeXchange Consortium via the PRIDE partner repository with the dataset identifier PXD019201. To unravel underlying pathomechanisms, in silico-based pathway analyses were performed including GO-term analysis as well as proteomaps considering the increased and decreased proteins separately.

#### Cellular processes influenced by proteins with increased abundance

**Fig. 2:**
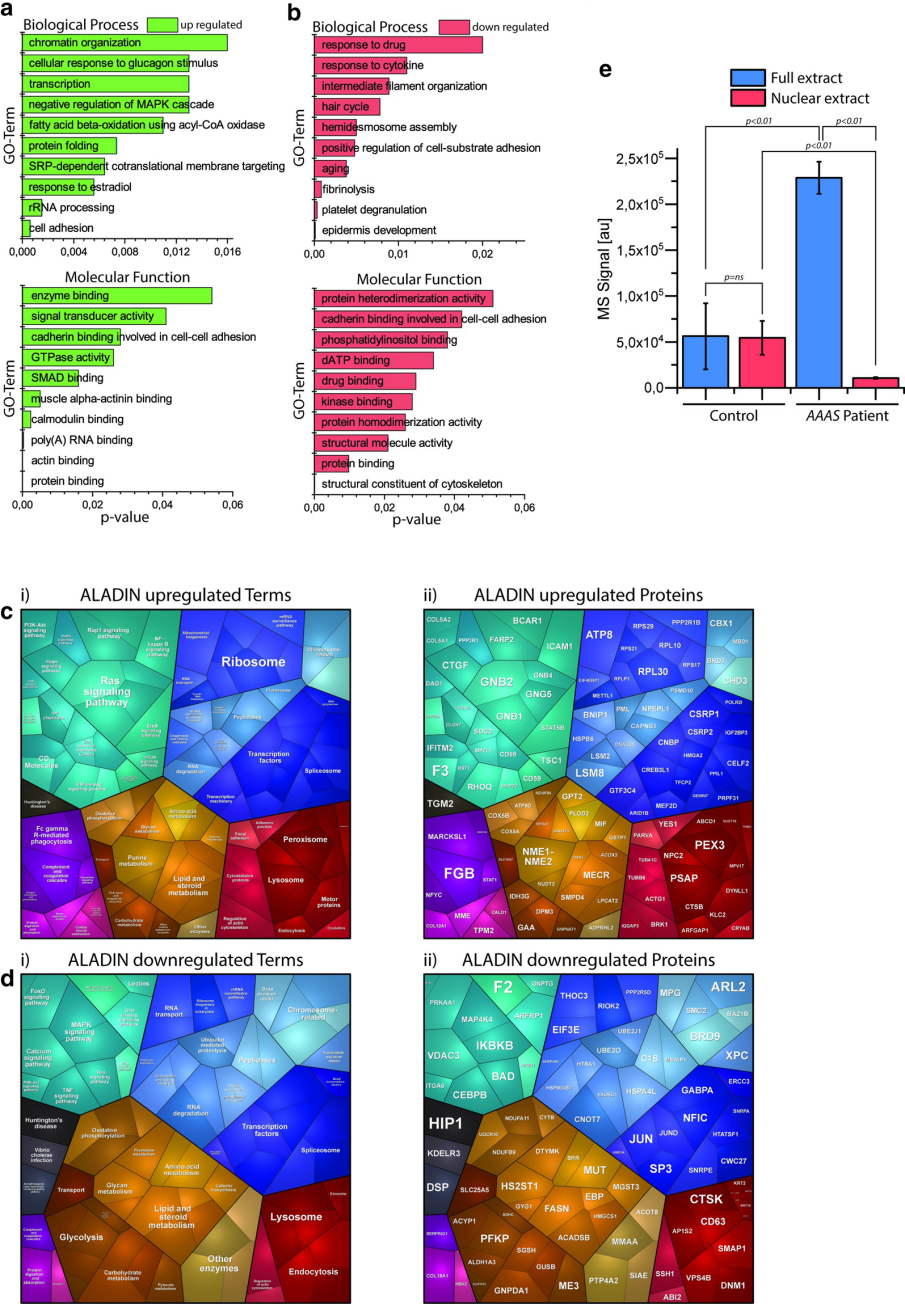
Results of the GO-Term (**a**) and the in silico pathway analysis (**b**) of all significant regulated proteins utilizing the Proteomaps platform: **a**, **b** GO-Term analysis of all significant regulated proteins showing the 10 most significant up regulated (green) and down regulated (red) GO-Terms of the categories biological process and Molecular function. **c** Illustration of the in silico pathway analysis using Proteomaps for elevated proteins. (i) is showing all significant pathways affected by the individual proteins which are highlighted in (ii). **d** Illustration of the in silico pathway analysis using Proteomaps for downregulated proteins. (i) is showing all significant pathways affected by the individual proteins which are highlighted in (ii). The colour code refers to signalling (turquoise), genetic processing (blue), cellular processes (red), metabolism (orange), organismal systems (purple) and human diseases (black). **e** Comparison of the relative expression levels of Synaptopodin-2 measured in the whole protein extract (blue) and nuclear protein extract (red) of control and patient fibroblasts. Number of biological replicates of each condition was 4 (n = 4). Abbreviations: au (arbitrary units), ns (not significant)

Our GO-term based analysis focussing on biological processes, molecular functions and cellular compartments revealed that proteins increased in the patient-derived cells are among others affecting cell adhesion, transcript production and processing, protein folding, fatty acid oxidation, signal transduction and cytoskeleton (relevant for muscle fibre contraction) (Fig. 2a). Moreover, a vulnerability of the functional ER-Golgi network crucial for protein processing can be deduced from our proteomic data (Additional file 3: Table 3), a molecular observation which accords with the known sub-cellular localization of the ALADIN protein within the endoplasmic/sarcoplasmic reticulum [11]. A proteomaps-based pathway analysis revealed altered regulation of signalling processes (including Ras-signalling), transcript-production, -processing and -translation (the latter is indicated by altered abundance of several ribosomal proteins) as well as metabolic processes including glycan-, purine- and lipid-metabolism (Fig. 2c i, ii). Moreover, vulnerability of muscle cell contraction as well as of regular protein clearance is indicated by the results of the proteomaps-based pathway analysis focussing on proteins presenting increased abundance in Allgrove-patient derived fibroblasts (Fig. 2c i, ii). Focussing on proteins of neurological/neuromuscular relevance it is important to note that a variety of such causative for or involved in the manifestation of neurological/neuromuscular disorders are increased in abundance. Among others, these proteins include Collagen alpha-1(XII) chain (MIM: 616471), Neprilysin (MIM: 617017 & 617018), Periostin [12], Synaptopodin-2 [13], Lysosomal alpha-glucosidase (MIM: 232,300), Ataxin-2 (MIM: 183090), Fragile X mental retardation syndrome-related protein 2 [14], Dystroglycan (MIM: 613818), Sphingomyelin phosphodiesterase 4 [15], Tropomyosin beta chain (MIM: 609285 & 108120), Atlastin-1 (MIM: 613708 & 182600), Alpha-crystallin B chain (MIM: 608810 & 613869), Dynein light chain 1 [16], Neuronal calcium sensor 1 [17] and Dolichol-phosphate mannosyltransferase subunit 3 (MIM: 612937) (Additional file 3: Table 3).

#### Cellular processes influenced by proteins with decreased abundance

Focussing on proteins presenting with decreased abundances in patient-derived fibroblasts, GO-term analysis revealed—among others—vulnerability of proteins involved in aging, structural molecule activity, protein binding and homodimerization (including kinases and cadherins) intermediate filament and also organization of the contractile apparatus (Fig. 2b). Proteomaps-based studies of cellular processes altered by decreased protein abundances also revealed altered signal transduction processes including Ca^2+^-, FoxO- and MAP-kinase signalling as well as again altered transcript-production, -processing and -translation. Moreover, vulnerability of the protein clearance machinery in addition to perturbed metabolic processes including glycolysis, amino acid turnover and lipid-metabolism is (in accordance with the indicated cellular vulnerabilities reflected by the increased proteins) indicated upon loss of the functional ALADIN in fibroblasts (Fig. 2d i, ii). Focussing on proteins of neurological/neuromuscular relevance it is worth noting that a variety of such causative for or involved in the manifestation of neurological/neuromuscular disorders are decreased in the patient-derived fibroblasts. Among others, these proteins include Atlastin-3 (MIM: 615632), Huntingtin-interacting protein 1 [18], Dynamin-1 (MIM: 616346), Heat shock protein HSP 90-beta [19, 20] and Glycogenin-1 (MIM: 613507 & 616199) (Additional file 3: Table 3).

#### Nuclear proteomic profiling of Allgrove-patient derived fibroblasts

Prompted by (i) the results of our global proteomic profiling revealing the differential expression of a variety of nuclear-resident proteins as well as by (ii) the known function of ALADIN as nucleoporin along with (iii) the myonuclear abnormalities observed in the muscle biopsy of the patient [10], we further aimed to study the impact of loss of functional ALADIN on nuclear protein composition. After enrichment of nuclei derived from patient and control fibroblasts and subsequent mass spectrometric analysis, ten proteins were found to be increased whereas nine were decreased in the nuclear fractions of patient-derived cells (Additional file 4: Table 4) confirming the concept of nuclear vulnerability upon loss of functional ALADIN. Next, proteins statistically significantly altered in both, the global and the nuclear proteome were selected to identify potential miss-targeting events. Indeed, this strategy resulted in the delineation of three proteins out of which two presented with increased abundance in both profiles (Periostin & Collagen alpha-1(XII) chain), whereas one (Synaptopodin-2) was reduced in the nuclear but increased in the global proteomic profile (Additional file 2: Table 2 and Additional file 3: Table 3; Fig. 2).

#### Validation of proteomic findings and further cellular studies

##### Lipid staining in fibroblasts

**Fig. 3:**
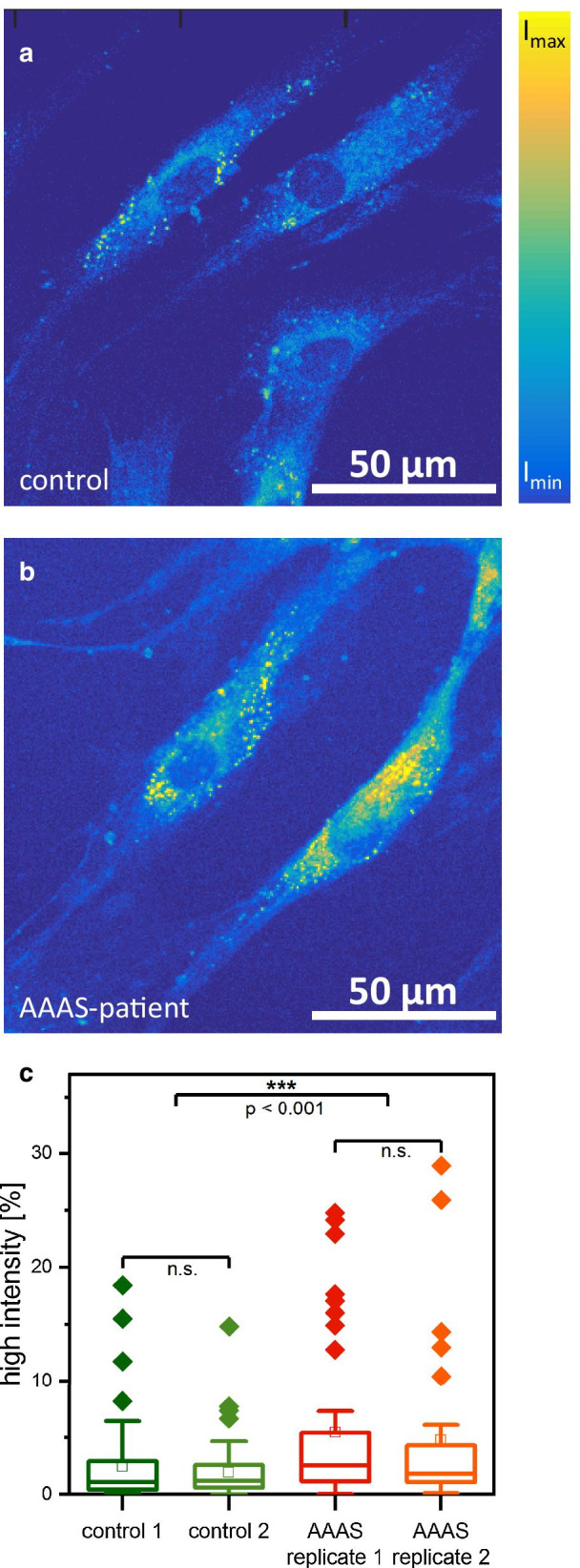
Single cell histogram analysis of BODIPY stained skin fibroblasts: **a** control sample and **b** *AAAS* mutant sample (Allgrove-patient), **c** evaluation of fluorescence intensity. Two control samples and two replicates of the *AAAS* mutant sample were analyzed. Hereby, at least 30 individual cells per sample were chosen for statistical analysis. For each cell, a histogram analysis of intensity values was performed, results were normalized to percentage values and the percentage values of all intensities exceeding 50% of I_max_ were summed up. The resulting data are depicted as box plot diagrams. Cells from AAAS samples have significantly more pixels of high intensity than control samples (*p* < 0.001)

Prompted by the known crucial role of the nuclear envelope/endoplasmic reticulum in lipid biosynthesis [21] as well as by altered expression of proteins involved in lipid maintenance and metabolism such as Perilipin-2, we hypothesized that loss of functional ALADIN impacts on proper cellular lipid homeostasis. To address this assumption, BODIPY-staining was performed in control and Allgrove-patient derived fibroblasts. Results of microscopic inspection revealed a statistically significant increase of BODIPY-fluorescence-intensity in patient-derived cells compared to controls (Fig. 3). This result not only accords with our proteomic findings and the regular subcellular localization of ALADIN but also suggests that altered lipid turnover might be part of the pathophysiological cascades leading to clinical manifestation of Allgrove syndrome.

##### CARS-microscopic studies on muscle biopsy

**Fig. 4:**
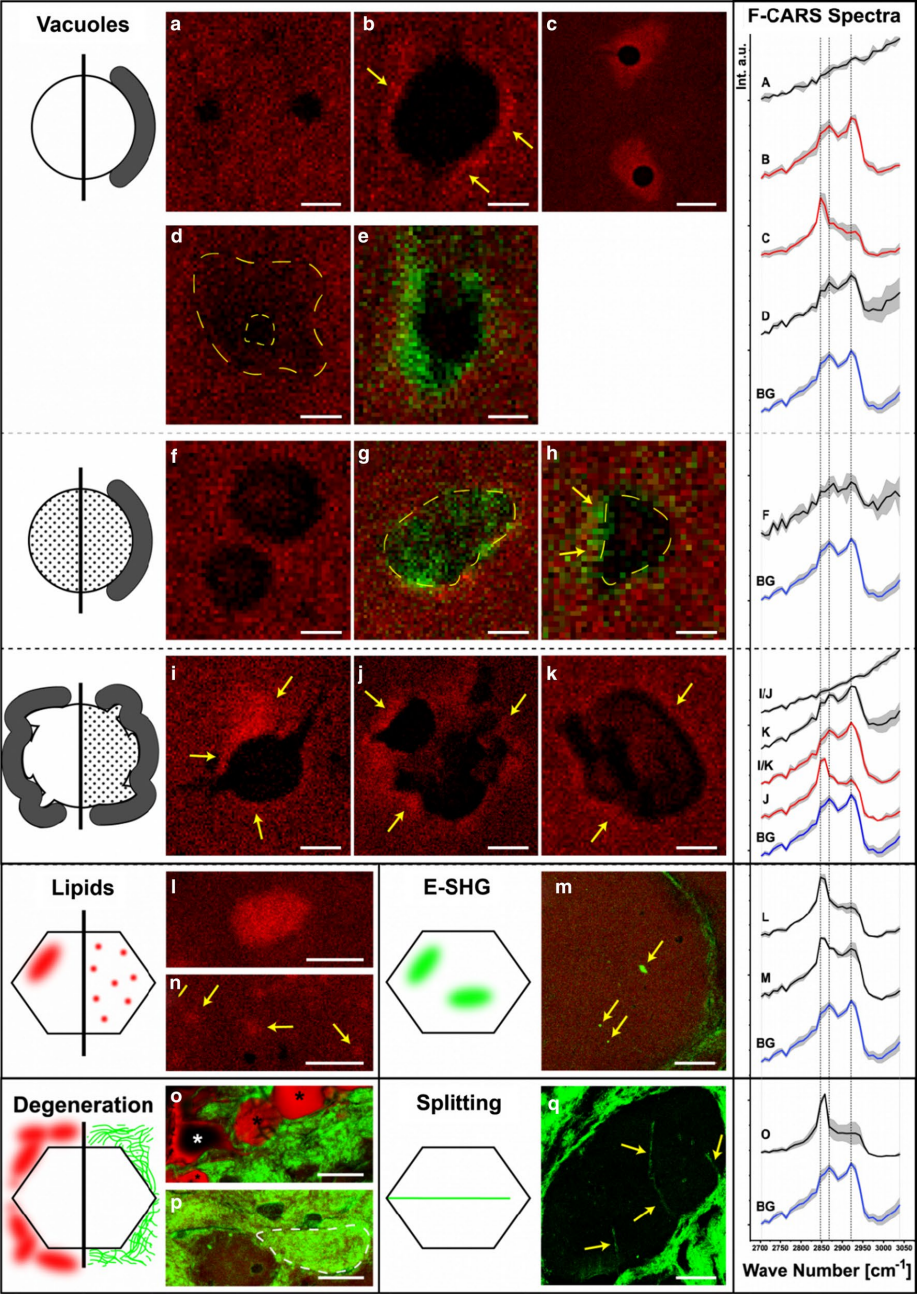
CARS microscopic findings in the muscle biopsy of an Allgrove syndrome patient: F-CARS images are shown in red and E-SHG images in green. Most CARS/SHG images were taken at 2932 cm^−1^, representing protein distribution. The remaining images show lipid distribution at 2841 cm^−1^ (C, L, M, O). “Empty” vacuoles with and without rims: **a** Without rim (scale bar 2.0 µm). **b** With protein rim (marked with arrow; 1.7 µm). **c** With lipid rim (6.3 µm). **d** Fuzzy border (external dotted line marks the assumed boundary; inner dotted line surrounds the "empty" centre; 1.8 µm). **e** With E-SHG rim (1.45 µm). Filled vacuoles with and without rims: **f** Protein filled without rim (1.4 µm). **g** ‘E-SHG filled’ without rim (dotted line marks border; 1.95 µm). **h** Protein filled with ‘E-SHG rim’ (dotted line marks border, arrows mark rim; 1.1 µm). “Empty” and filled amorphous vacuoles with rims: **i** Empty with protein rim (arrows mark rim; 3.0 µm). **j** Empty with lipid rim (arrows mark rim; 4.8 µm). **k** Protein filled with protein rim (arrows mark rim; 2.25 µm). Lipid accumulations: **l** Cloud-like accumulation (7.1 µm). **m** Dot-like accumulations (marked with arrows; 7.7 µm). E-SHG accumulation: **n** Cloud-like accumulation (marked with arrows; 14 µm). Degeneration: **o** Fatty degeneration (marked with asterisks; 39 µm). **p** Fibrous degeneration (dotted line shows the borders of a former muscle fibre, which has been replaced by connective tissue; 17 µm). Fibre splitting: **q** Splitting (marked with arrows; 60 µm). F-CARS spectra: The spectra were obtained from measurements of the corresponding feature findings. Black spectra were taken from the inside of vacuoles or lipid accumulations (L, M, O). Red spectra were taken from vacuole rims. The cell background (blue) was taken from inconspicuous areas near the features. The standard deviation is shown in grey. The vertical dotted lines highlight the wave numbers 2847 cm^−1^, 2868 cm^−1^ and 2921 cm^−1^

To further elucidate the finding of altered lipid homeostasis in fibroblasts we next analysed the biochemical composition of muscle biopsies without any staining, labelling or pre-treatment using coherent anti-Stokes Raman scattering (CARS). CARS microscopy and subsequent data evaluation on muscle biopsies of an Allgrove syndrome patient revealed an increased variability in fibre size [patient: 60.02 µm ± 35.72 µm (based on 103 investigated fibres); control 1: 39.38 µm ± 7.23 µm (based on 318 investigated fibres); control 2: 43.48 µm ± 10.49 µm (based on 184 investigated fibres); control 3: 36.94 µm ± 6.07 µm (based on 89 investigated fibres)] and confirmed some of the feature findings reported by [10] including the presence of rimmed and non-rimmed vacuoles. However, here we moreover sorted 11 vacuole variants into 3 groups (Fig. 4a–k): five variants were assigned to the group of "empty vacuoles” with and without a rim. This type of vacuole is characterized by such a low intensity inside that it appears empty compared to the surrounding area. Within this group, the vacuoles have either no rim (Fig. 4a), a protein rim (Fig. 4b) or a lipid rim (Fig. 4c). Furthermore, some "empty vacuoles” present with broad fuzzy boundaries and just a small “empty” core (Fig. 4d). Other "empty vacuoles” show rims with a second harmonic generation signal (SHG) detected in epi direction (E-SHG) (Fig. 4e) indicating a highly organized substance. The second group comprises “filled vacuoles” with and without rim. The substances within the vacuoles have either a CARS signal detected in forward direction (F-CARS) (Fig. 4f, h) or an E-SHG signal (Fig. 4g). In addition, F-CARS “filled vacuoles” may present with a rim characterized by an E-SHG signal (Fig. 4h). Vacuoles of the third group are characterized by their amorphous form: they can appear both as "empty” (Fig. 4i, j) and “filled” (Fig. 4k) amorphous vacuoles. The "empty amorphous vacuoles” can have a protein rim (Fig. 4i) or a lipid rim (Fig. 4j). In contrast, the “filled amorphous vacuoles” show only protein rims (Fig. 4k). No “amorphous vacuole” presented with an E-SHG signal.

In addition to the vacuoles, our CARS-microscopic studies revealed changes in lipid distribution: “cloud-like” (Fig. 4l) and “dot-like” (Fig. 4m) lipid accumulations in fibres were detected. Another type of “cloud-like” accumulations shows only a pure E-SHG signal (Fig. 4n). Two forms of degeneration were also identified: lipid-filled areas with fibre-like shape (Fig. 4o, asterisk) are attributed to fatty degeneration [22]. Increased amounts of connective tissue around muscle tissue were detected by E-SHG signals (Fig. 4p). Moreover, connective tissue surrounding muscle fibres was also detected (Fig. 4p, dashed line). Both findings indicate fibrous degeneration. Further degenerative processes are indicated by the identified fibre splitting (Fig. 4q).

The F-CARS raw spectra employed to study these features were first normalized. From these, the mean spectra with standard deviations were calculated (Fig. 4, F-CARS spectra). We identified that the standard protein peak at 2932 cm^−1^ [23, 24] is shifted to 2921 cm^−1^. Additionally, the standard peak of ordered lipid [25] at 2889 cm^−1^ seems shifted to 2868 cm^−1^. We consider the vibrational bands at 2932 cm^−1^ and 2921 cm^−1^ as a single vibrational band [26]. The peak of disordered lipids is at 2847 cm^−1^ [25]. The signal ratio 2921/2847 represents the proportion of protein to lipid (Table 1). All spectra are compared to the fibre background (Fig. 4b, g), except the ones representing the lack of content in “empty vacuoles” (steady slope without any discernible peaks). The substance in “filled vacuoles” (Fig. 4f) shows a slightly reduced protein proportion compared to lipid. The substance in the “filled amorphous vacuoles” (Fig. 4k) shows an increased protein share. The ratio of the fuzzy border vacuoles (Fig. 4d) is like the substance from the “amorphous filled vacuoles”. The F-CARS raw spectra of the content of the “filled vacuoles” and the broad borders of the “fuzzy vacuole” have less signal intensity (factor 1.4–2) than the fibre background raw spectra (not visible in Fig. 4 due to normalisation). The protein rims show an increased protein to lipid ratio. The ratio of “amorphous vacuoles” protein rims (Fig. 4i, k) is greater than the one of "empty vacuoles” protein rims (Fig. 4b). Lipid rims are characterised by a protein to lipid ratio < 1 (Fig. 4c, j). The rims of “empty vacuoles” have a highest lipid ratio among the presented features. We are not aware of any previous reports for this feature.

**Table 1:** Ratio between intensities of wave numbers used for the detection of protein and lipid

	Ratio_2921/2847_
Fibre background	1.269
"Empty" vacuoles	
With protein rim	1.343
With lipid rim	0.413
With fuzzy border	1.346
Filled vacuoles	1.202
Amorphous vacuoles	
Filled (inner)	1.359
With protein rim	1.419
with lipid rim	0.691
Lipid accumulations	
Cloud-like	0.507
Dot-like	0.825
Fatty degeneration	0.442

The averaged spectra from Fig. 4 were used to calculate the ratios between 2921 and 2847 cm^−1^. Ratio_2921/2847_ show the relationship between the characteristic wave numbers of protein and lipid

Of note, two types of lipid accumulations were also identified in Allgrove syndrome patient-derived muscle fibres (Fig. 4l, m). The “cloud-like” lipid accumulations show a high lipid ratio. The “dot-like” lipid accumulations show a higher protein proportion than the lipid clouds, but still have a higher lipid than protein signal. In addition to the lipid rims, we also detected areas of fatty degeneration (Fig. 4o). As expected, these areas show a high lipid amount.

##### Immunofluorescence on muscle biopsy

**Fig. 5:**
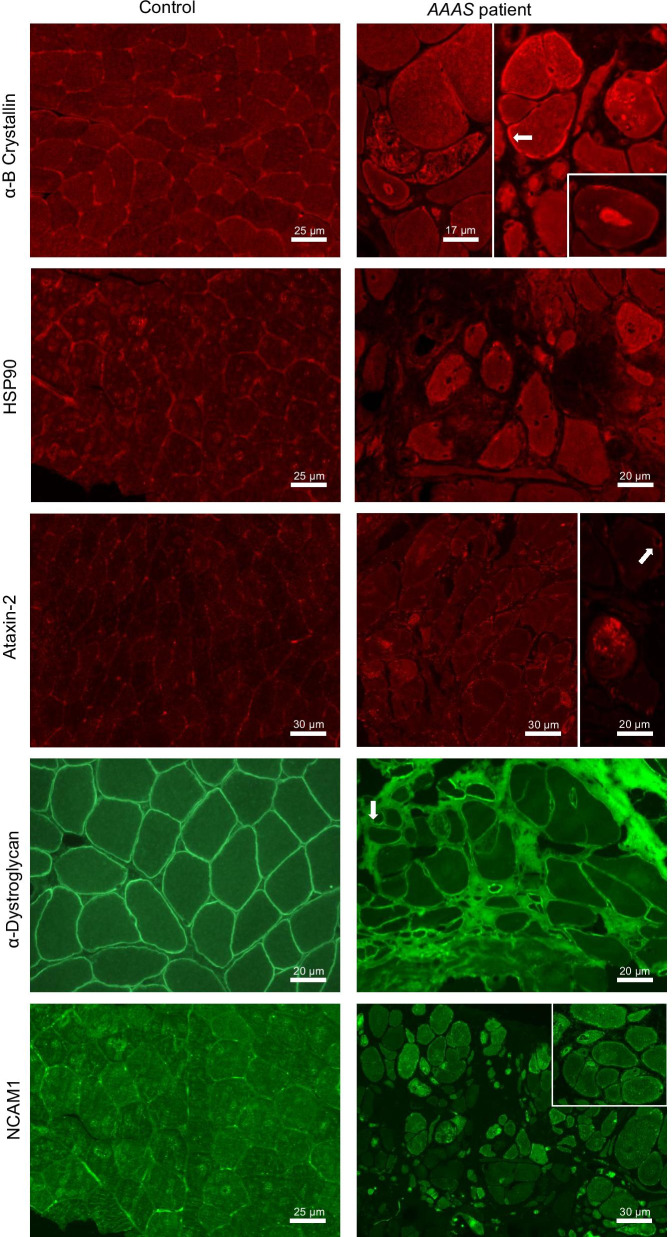
Immunofluorescence-based verification studies of proteomic findings in muscle cells: In comparison to control muscle cells, in the muscle biopsy derived from the Allgrove patient, focal sarcoplasmic and subsarcolemmal enrichment (white arrows) of α-B-Crystallin and HSP90 is found referring to an increase of both cytosolic chaperones. In addition, increase of dots immunoreactive for Ataxin-2 is found in the sarcoplasm and in perinuclear location (white arrow) in the Allgrove patient’s biopsy. Studies of α-Dystroglycan demonstrate uneven, partially increased sarcolemmal immunoreactivity and small immunoreactive sarcoplasmic dots (white arrow) in some fibers. Immunological detection of NCAM1 shows the frequent presence of sarcoplasmic dots of varying size. Moreover, immunological detection of Periostin shows a profound immunoreactivity within the connective tissue with intense foci. FITC-Phalloidin staining visualizing actin unravels perimyonuclear regions with abnormal accumulation (white arrows) as well as subsarcolemmal enrichment (white stars) in a proportion of fibers in the biopsy of the Allgrove patient. Staining of Synaptopodin-2/Myopodin utilizing two different antibodies (M2 & HH9) shows particularly subsarcolemmal enrichment (white arrows) in a proportion of fibers in the biopsy of the Allgrove patient

Results of our global proteomic profiling revealed the altered expression of a variety of proteins known to be causative for or involved in the molecular genesis of neurological diseases. Therefore, vulnerability of paradigmatic ones with known relevance for the development of muscular diseases was confirmed in a muscle biopsy derived from the same Allgrove-patient exhibiting a profound myopathology as described previously [10]. Results of our immunofluorescence-based validation studies revealed an extracellular increase of Periostin. Moreover, uneven sarcolemmal immunoreactivity including focal increases for α-Dystroglycan and focal sarcoplasmic dots/accumulations immunoreactive for α-B-Crystallin and for HSP90 were identified in addition to subsarcolemmal increase of α-B-Crystallin (white arrow) (Fig. 5). In addition, increased abundance of sarcoplasmic dots immunoreactive for Ataxin-2 and perinuclear enrichment of Ataxin-2 (white arrow) was detected. Moreover, sarcoplasmic increase of NCAM1 was identified (Fig. 5). Prompted by the altered expression of proteins involved in actin-cytoskeleton as well as significant increase of actin itself in Allgrove-patient derived skin fibroblasts, FITC-Phalloidin-staining was carried out to study actin-distribution in patient-derived muscle biopsy specimen. Results of these studies showed some focal subsarcolemmal sarcoplasmic increases (white stars) in addition to perinuclear enrichment (white arrows) of actin (Fig. 5). Given that ultra-structural studies of the Allgrove-patient derived muscle biopsy revealed fine filamentous structures in subsarcolemmal location and fibrillary nuclear bodies, immunofluorescence-based analysis of Synaptopodin-2/Myopodin (displaying actin-bundling activity) as a protein altered in both, the global and the nuclear proteome was performed using two different antibodies (M2 and HH9). Results of these studies revealed increased immunoreactivity at and below the sarcolemma (white arrows) as well as focal enrichment within the sarcoplasm (white star) as also observed for actin (by FITC-Phalloidin staining) (Fig. 5).

## Discussion

### Protein cataloguing in human fibroblasts reveals expression of a variety of proteins of neurological relevance

Although collection of muscle and/or nerve biopsies represented a standard procedure in the diagnostic work-up of patients suffering from neuromuscular diseases in the past, this approach became less essential especially in the light of the rapidly developing omics approaches toward the identification of the underlying genetic defect [27]. Although this is less invasive and stressful for the patients, in turn it causes a lack of biomaterial needed for protein studies aiming to address the pathogenicity of identified genetic variants and/or to elucidate pathophysiological cascades—a prerequisite for the definition of new therapeutic intervention concepts. Since fibroblasts have already been widely used for in vitro studies focusing on pathophysiological processes taking place in the development and manifestation of neurological diseases (e.g. [5, 6], we aimed to establish a comprehensive catalogue of proteins expressed in fibroblasts. Subsequently, this catalogue was screened for the occurrence of proteins relevant to processes with particular importance for the function and maintenance of muscle and neuronal cells. Indeed, by cataloguing the most abundant 8280 proteins and subsequent in silico based pathway analyses, we could demonstrate that in human fibroblasts a variety of proteins are expressed which control neurological processes such as muscle cell contraction or neuronal survival (Additional file 1: Table 1). This in turn shows that fibroblasts represent a cellular system suitable to study these processes in vitro. This is further highlighted by the expression of 257 proteins out of a list of 385 (66.8%) associated with the manifestation of genetically caused rare forms of neurological disorders. The finding that the coverage of proteins encoded by genes responsible for motoneuron and peripheral nervous system diseases is much higher than the one for muscle disorders (Additional file 2: Table 2) might be caused by the fact that fibroblasts and neuronal cells derive from the same embryotic layer, the ectoderm bearing the neuroectoderm. In contrast, muscle cells are derived from mesoderm.

Focusing on individual proteins, it is worth noting that Dystroglycan has been identified to be expressed in fibroblasts (Additional file 1: Table 1). This might open new avenues in (i) the diagnostic work-up of patients with the clinical suspicion of a so-called “dystroglycanopathy” making the collection of a muscle biopsy less essential as well as in (ii) the study of the precise molecular nature of this disease group and (iii) to test new therapeutic concepts pre-clinically by having the chance to make use of human material with different genetic defects leading to “dystroglycanopathies” [28]. This postulate is supported by the results of our proteomic and immunofluorescence studies on skin fibroblasts and muscle cells derived from a patient with Allgrove syndrome (see below).

### Proteomic studies on a use case allowed insights into the molecular etiology of the disease

#### Relevance of proteomic- and BODIPY-findings for neurological signs of Allgrove syndrome

The Allgrove syndrome also known as triple A syndrome (AAAS) is caused by biallelic mutations in the *AAAS* gene, encoding a 546 amino acid-sized nuclear pore complex protein known as ALADIN (alacrima achalasia adrenal insufficiency neurologic disorder). ALADIN belongs to the WD-repeat family of regulatory proteins. The broad neurological manifestations of the disease occur later in the course of the disease and Allgrove syndrome patients show a wide variability [29]. Neurodegeneration can affect spinal motoneurons, Purkinje cells, striatal neurons and the autonomic system and the precise molecular mechanisms leading to neuronal loss are still unclear [30]. A recent functional study on central nervous system tissues and fibroblasts of a novel Allgrove syndrome patient (homozygous *AAAS* mutation c.464G>A; p.R155H) presenting with a motor neuron disease, cerebellar ataxia (accompanied by severe loss of motor neurons and Purkinje cells) and autonomic dysfunction revealed significant reduction in the perinuclear expression of p.R155H-mutant ALADIN in the patient's brain correlating with a significant reduction of the *AAAS-1* transcript, while the *AAAS-2* transcript was upregulated in fibroblasts [30]. Taken together, this neuropathological study demonstrated the effect of loss of functional ALADIN in the human central nervous system. Of note, results of our proteomic study indicated altered expression of a variety of proteins decisive for proper neuronal function. This molecular finding might not only provide first insights into the biochemical origin of the broad neurological involvement of Allgrove syndrome but also confirms the suitability of fibroblasts to identify pathophysiological processes of neuronal relevance: Ataxin-2, a polyglutamine protein causative for spinocerebellar ataxia type 2 upon repeat expansion > 34, was shown to function as modifier of Amyotrophic Lateral Sclerosis (ALS) pathology (as a rapidly progressing neurodegenerative disease) with considerable negative impact of Ataxin-2 expression on pathophysiology [31, 32]. Therefore, we hypothesize that increased Ataxin-2 expression as identified by our unbiased proteomic profiling contributes to the pathophysiology of Allgrove syndrome. This assumption is supported by the identification of focal sarcoplasmic and perinuclear enrichment of Ataxin-2 in Allgrove-patient derived muscle cells. Interestingly, a trigger of these pathophysiological processes was associated with elevated stress of the ER, the subcellular compartment to which ALADIN localizes. In the same context, overexpression of human recombinant Sphingomyelin phosphodiesterase 4 (SMPD4; increased in fibroblasts of our patient) shows localization to the outer nuclear envelope and the ER and additionally revealed interactions with several nuclear pore complex proteins as identified by proteomics analysis [15]. In addition, fibroblasts derived from *SMPD4* patients suffering from a developmental disorder characterized by microcephaly and congenital arthrogryposis show ER abnormalities and are more prone to apoptosis under stress conditions. Based on their findings, Magini and co-workers postulated that SMPD4 links homeostasis of membrane sphingolipids to cell fate by regulating the crosstalk between the ER and the outer nuclear envelope [15]. Thus, increased level of SMPD4 in cells lacking functional ALADIN accompanied by the identification of altered lipid homeostasis (dysregulated lipid metabolism has already been linked to a variety of neurodegenerative disease, e.g. [33] as verified by the results of our BODIPY-staining support the concept of a crosstalk between lipid homeostasis and nuclear envelope integrity. Moreover, these combined findings indicate that perturbed lipid homeostasis may be part of the molecular etiology of Allgrove syndrome.

#### Relevance of proteomic and CARS/SHG-microscopic findings for myopathology of Allgrove syndrome

Ultra-structural studies of a muscle biopsy derived from our Allgrove-patient revealed foci of disorganized myofibrils, occasionally vacuole-associated, dilatations of sarcoplasmic reticulum, (containing a fine granular structure), abnormal mitochondria, myonuclei with peripheral enriched heterochromatin and irregular invaginations in addition to the rare finding of intra-myonuclear large diameter tubulo-filamentous inclusions [10]. Interestingly, results of our unbiased proteomic profiling and subsequent verification studies revealed vulnerability of proteins which accords with these pathomorphological findings: altered expression of a variety of mitochondrial proteins—presumably as a biochemical correlate to initiated apoptosis—is in line with perturbed mitochondrial integrity. Increase of chaperones reflecting perturbed protein processing as identified by proteomic profiling of skin fibroblasts could be verified in muscle cells of the same patient by showing a profound increase of sarcoplasmic dots immunoreactive for α-B Crystallin and HSP90. This finding accords with the previous finding of vacuole-associated dilatations of sarcoplasmic reticulum [10] a pathomorphological finding indicative for ER-stress. Results of our CARS-microscopic studies allowed a more precise molecular stratification of these vacuoles and revealed varying compositions of vacuole content and rim. This may indicate vacuoles at different stages of maturation [34, 35] with early autophagic vacuoles (amorphous), late autophagic vacuoles (filled) and autolysosomes (empty) being present simultaneously in the sample. The different rim compositions may either be due to autophagized substances that are released into the cytosol and/or membrane-bound degradation proteins. This is, to the best of our knowledge, the first time, that the chemical composition of rimmed vacuoles has been determined in an untargeted and label-free approach. Furthermore, in accordance with our BODIPY-findings obtained in patient-derived fibroblasts (see above), results of our CARS microscopic studies led to the identification of lipid aggregates suggesting that altered lipid homeostasis is part of the underlying pathophysiology across different cellular populations. Given that bulk membrane lipid biogenesis in primary cells largely occurs in the endomembrane compartment, which includes the domains of the ER [36] and the ER is known to be important for proper lipid transportation to other organelles such as mitochondria [37], one might assume that loss of functional ALADIN perturbs proper lipid transportation leading to irregular cellular build-up. The above-mentioned pathological findings in mitochondria might support this assumption. The previous identification of disorganized myofibrils [10] might correlate with the focal sarcoplasmic enrichment of Actin and Synaptopodin-2 (as an Actin-bundling protein). This finding hints toward a vulnerability of this subcellular structure to which wild type ALADIN localizes. Focal subsarcolemmal and sarcoplasmic increase of Synaptopodin-2 not only indicates an impact of this protein (which is also involved in the modulation of autophagy; [38] in the molecular myopathology of Allgrove syndrome but also accords with our proteomic findings obtained in both proteomic studies on skin fibroblasts derived from the same patient. Given that an adverse effect of elevated Ataxin-2 expression in the neuronal pathophysiology of ALS is known [31, 32], the finding of Ataxin-2 increase in the muscle biopsy derived from the Allgrove patient suggests an impact of elevated Ataxin-2 expression also in myopathological processes. Changes in the level of NCAM1 expression and localization in muscle cells correspond to key morphogenetic events during muscle differentiation and repair [39]. Our immunological findings on α-Dystroglycan (verifying the proteomic findings obtained in fibroblasts) highlight a secondary vulnerability of this protein in the molecular etiology of Allgrove syndrome and thus add this multisystemic disease to the growing list of so-called secondary dystroglycanopathies.

## Conclusions

Intersection of a spectral/protein library obtained from cultured human skin fibroblasts with a list of proteins known to be causative for the manifestation of neurological diseases shows that approximately 80% of proteins causative for motoneuron diseases or disease of the peripheral nervous system are abundant in these cells. Moreover, approximately 40% of proteins encoded by genes causative for diseases of the neuromuscular junction or of the skeletal muscle are present in our spectral/protein library covering the 8280 most abundant proteins. Proteomic profiling of fibroblasts derived from a patient suffering from Allgrove syndrome was carried out as a case example and findings allowed novel insights into the underlying pathophysiology also by adding perturbed lipid homoeostasis to the list of pathobiochemical findings known for this rare neurological disorder. In general, verification of protein-dysregulations as identified in skin fibroblasts in muscle cells derived from the same patient in turn demonstrates the suitability of skin fibroblasts to identify and further study processes relevant for the manifestation of neurological diseases.

## Patient, materials and methods

### Allgrove-patient derived and control skin fibroblasts and muscle biopsy specimen

The Allgrove-patient was identified early in the second decade of life when declining school performance together with mild dysmorphic features lead to suggestion of a neurological disorder. He presented with the well-described signs of type II achalasia and the combination of predominately distal muscle weakness and atrophy with brisk deep tendon reflexes. Clinical severity and manifesting age define this as a case of intermediate severity, although there is no established grading system.

The muscle biopsy derived from this genetically confirmed Allgrove patient (c.762delC mutation in the nucleoporin gene *AAAS*; [10]) was collected for diagnostic purpose including histological and electron microscopic investigations. In the same procedure, a skin biopsy was collected and used to set-up a fibroblast culture now available for further functional studies focusing on the cellular impact of loss of functional ALADIN. Primary human fibroblasts from the Allgrove-patient and controls (n = 3; obtained from the MRC Centre Neuromuscular Biobank Newcastle [40]) were cultured in minimum essential media supplemented with 10% fetal bovine serum, 1% penicillin/streptomycin, 2 mM l‐glutamine, 1 × non‐essential amino acids, 1 × minimum essential medium vitamins, 1 mM sodium pyruvate, 50 μg/mL uridine (ThermoFisher Scientific), at 37 °C, in a humidified 5% CO_2_ atmosphere. Cells were grown to a confluency of 70% prior collection for proteomic studies: cells were collected in a 1.5 ml tube, washed twice with ice-cold PBS, snap-frozen in liquid nitrogen and stored at -80 °C until protein extraction for subsequent proteomic studies (see below).

### Sample preparation of fibroblasts for spectral library generation

Snap-frozen fibroblasts were lysed in a buffer containing 1% SDS, 50 mM Tris, 150 mM NaCl, pH 7.8 and cOmplete™ ULTRA protease inhibitor using the Bioruptor® (Diagenode) for 10 min (30 s on, 30 s off, 10 cycles) at 4 °C. Afterwards, 20 µl of each sample was taken and diluted 1:4 with 10 mM ammoniumbicarbonate buffer, pH 7.8 (ABC) to conduct a BCA-based determination of protein concentration according to the manufacturer’s instructions (Pierce BCA protein assay kit). Reduction and carbamidomethylation of the remaining samples were carried out using 10 mM Tris-(2-carboxyethyl)-phosphin (TCEP) for 30 min at 37 C followed by application of 15 mM Iodacetamide (IAA) for further 30 min at room temperature (RT).

Samples were further processed using the S-Trap™ (Protifi) sample preparation procedure: after acidifying the samples by adding 12% aqueous phosphoric acid, they were diluted with S-Trap binding buffer (90% methanol (MeOH), triethylammonium bicarbonate (TEAB) 100 mM, pH 7.1). Protein-loading to S-Trap columns including centrifugation steps was performed according to the manufacturer’s instructions. Filter-based tryptic digestion was carried out for 2 h at 47 °C using a trypsin to protein ratio of 1:20. Afterwards, peptides were eluted by applying multiple eluting steps starting with 10 mM ABC followed by elution with 0.1% formic acid (FA) and last with 80% acetonitrile (ACN). Drying of the eluted peptides was performed in a vacuum concentrator followed by dissolving of peptides in 0.1% TFA for subsequent LC-MS/MS analysis or 10 mM ammonium acetate with 0.4 mM FA (pH 8.0) for subsequent pH8 reversed phase fractionation.

All proteolytic digests were analyzed using monolithic column separation system (PepSwift monolithic PS-DVB PL-CAP200-PM, Dionex) on an inert Ultimate 3000 HPLC (Dionex) by direct injection of 1 μg sample to proof effectiveness of tryptic digestion. A binary gradient (solvent A: 0.1% TFA, solvent B: 0.08% TFA, 84% ACN) ranging from 5 to 12% B in 5 min and then from 12 to 50% B in 15 min at a flow rate of 2.2 μl/min and at 60 °C, was applied. UV traces were acquired at 214 nm [41].

### PH8-based sample fractionation

Each of the digested and desalted samples selected for the following generation of a spectral library was first dried using a vacuum concentrator. The peptides were then dissolved in a buffer containing 10 mM ammonium acetate and 0.4 mM formiate (pH 8.0) (concentration 50 μg/μl) and separated on a C18 RP chromatography column (loading amount 50 μg). Doing so, peptides were loaded onto the column with solvent A (10 mM ammonium acetate, 0.4 mM formiate, pH 8.0) at a flow rate of 12.5 μl/min. Separation and fractionation were performed with the following gradient with solvent B (84% acetonitrile in 10 mM ammonium acetate, 0.4 mM formiate, pH 8.0): 3–10% in 10 min, 10–25% for 35 min, 25–40% for 20 min, 40–95% for 10 min, 95% for 5 min and 20 min equilibration at 3%. The individual fractions were collected in an interval of 60 s, with each sample divided into 15 fractions. Collection was performed in the time interval of 10 to 75 min of the gradient. The fractions were collected in combined form, so that after reaching the last fraction, the sample was collected again for one minute in fraction 1. After fractionation, the individual samples were dried in a vacuum concentrator and dissolved in 0.1% TFA prior subsequent nano LC-MS/MS analysis (1 μg/μl).

### Spectral library generation

Given that setting up a spectral library is a prerequisite to perform a data independent LC–MS/MS-based sample analysis, all fractions derived from the pH8 fractionation mentioned above were analyzed by nano LC-MS/MS using 1 µg respectively: samples were loaded on an Ultimate 3000 Rapid Separation Liquid chromatography (RSLC) nano system with a ProFlow flow control device coupled to a Fusion Lumos Tribrid mass spectrometer (both from Thermo Scientific). After initial loading, peptides were concentrated on a trapping column (Acclaim C18 PepMap100, 100 µm, 2 cm) using 0.1% TFA at a flowrate of 10 µl/min. Following sample separation was accomplished on a reversed phase column (Acclaim C18 PepMap100, 75 µm 50 cm) using a binary gradient: 3% solvent B (84% ACN with 0.1% TFA) for 10 min, a linear increase of solvent B to 35% for 120 min, a linear increase of solvent B to 95% for 10 min followed by a linear decrease of solvent B to 3% for 5 min. MS survey scans were acquired on the Fusion Lumos using settings as follows: mass spectrometer was operated in data dependent acquisition mode (DDA) with full MS scans from 300 to 1500 m*/z* at a resolution of 120,000 (Orbitrap) using the polysiloxane ion at 445.12002 m*/z* as lock mass. The automatic gain control (AGC) was set to 2E5 and the maximum injection time to 50 ms. The most intense ions above a threshold ion count of 5E3 were selected for fragmentation at a normalized collision energy (nCE) of 30% (HCD) in each cycle of the acquisition analysis, following each survey scan. The dynamic exclusion time was set to 15 s. The number of selected precursor ions for fragmentation was determined by the “rapid” acquisition algorithm. Fragment ions were acquired in the linear ion trap with an AGC of 1E4 and a maximum injection time of 35 ms.

The acquired data were imported into the software Spectronaut (Biognosys). As proteome background the human proteome data was selected from UniProt (www.uniprot.org) containing 20,374 entries. The processing settings were set as following: enzyme was trypsin, the minimum and maximum peptide length was set to 7 and 52 respectively, missed cleavages was set to 2. Carbamidomethyl for cystein was set as fixed modification and acetyl (Protein N-term) and oxidation of methionine was set as variable modifications. All settings regarding the library generation including tolerances, identification, filters, iRT calibration and workflow were set to factory defaults. For the relative quantification, the option Top N max 3 was taken meaning that for each protein the average of the 3 most intense identified peptides are taken to give the protein the quantitative value.

### DIA-LC-MS/MS analysis

For the data independent acquisition (DIA) approach, the same nano LC-MS/MS setup as for the DDA acquisition was used. Each sample analyzed was mixed with an appropriate amount of iRT standard (Biognosys) peptides and 1 µg of each sample was subjected to the analysis. Full MS scans were acquired from 300 to 1100 m*/z* at a resolution of 60,000 (Orbitrap) using the polysiloxane ion at 445.12002 m*/z* as lock mass. The automatic gain control (AGC) was set to 5E5 and the maximum injection time to 20 ms. Full MS scans were followed by 30 DIA windows acquired at a resolution 30,000 (Orbitrap) with an AGC set to 1E6, a maximum injection time of 60 ms and nCE of 32 (HCD).

### Analysis of DIA data

For the analysis of the samples acquired with nano-LC-MS/MS in DIA mode, the data was introduced to the Spectronaut software and analyzed with a library-based search. As library, the above created spectral library was used. Search and extraction settings were kept as standard (BGS Factory settings). As background proteome, same data were used as selected for the establishment of the spectral library.

### Preparation of nuclear protein fraction from fibroblasts

Snap-frozen fibroblasts (3 controls and 3 patient samples) were processed utilizing the “Qproteome Cell Compartment Kit” (Qiagen; Cat No./ID: 37502): frozen cell pellets were dissolved with 1 ml of extraction buffer CE1 (+ protease inhibitor) and incubated for 10 min at 4 °C on a Thermomixer (Eppendorf). After centrifugation (1000*g* for 10 min at 4 °C), supernatant was collected and pellets were dissolved in ice cold extraction buffer CE2 (+ protease inhibitor), followed by an incubation for 30 min at 4 °C (on a Thermomixer). After centrifugation (6000*g* for 10 at 4 °C), the supernatant was collected in a new reaction tube and Benzonase was added to the pellets followed by an incubation for 15 min at RT. Afterwards 500 µl ice cold extraction buffer CE3 (+ protease inhibitor) was added and samples were incubated for 10 min at 4 °C. Insoluble material was pelleted by centrifugation with 6800 g for 10 min at 4 °C and the supernatant containing the nuclear proteins was collected and stored at -80 °C until further processing.

After thawing nuclear proteins fractions on ice, samples were mixed with ice cold acetone and incubated at − 80 °C for 12 h. After centrifugation (20,000*g* for 20 min at 4 °C), supernatant was discarded, and the precipitated protein pellet was dried under a flow hood. Afterwards, 50 µl of 8 M Urea was added to dissolve the protein pellet at RT. After diluting the solution to 2 M urea with 10 mM ammonium bicarbonate buffer, pH 7.8 (ABC), a BCA-based determination of protein concentration was conducted according to the manufacturer’s instructions (Pierce BCA protein assay kit). Reduction and carbamidomethylation of the samples were carried out using 10 mM tris-(2-carboxyethyl)-phosphine (TCEP) for 30 min at 37 °C followed by application of 15 mM iodacetamide (IAA) for another 30 min at room temperature (RT). Samples were further diluted to 1 M urea using 10 mM ABC buffer adding trypsin (ratio 1:100) to warrant protein hydrolyzation over night at 37 °C. Next, the tryptic-digestion was terminated by adding 3 µl of 99% FA to decrease the pH to 2. Afterwards, samples were desalted using solid phase extraction with C18 filter cartridges, washed with 0.1% TFA and eluted with 80% ACN. Cleaned samples were dried by using a vacuum concentrator. Concentration was adjusted to 1 µg/µl with 0.1% TFA.

### Microscopic studies

#### Fibroblasts

Fibroblasts derived from the Allgrove patient as well as from respective sex and age matched controls were grown on coverslips, washed three times with 1xPBS, fixed with 4% formaldehyde solution for 20 min at room temperature and afterwards washed thrice with PBS. After 200 µL BODIPY 493/503 (ThermoFisher Scientific, diluted 1:1000 in PBS) was added to each coverslip, samples were incubated in a climate chamber for 1 h at room temperature in the dark. The staining solution was removed, samples were washed thrice in 1×PBS and mounted onto a slide utilizing a drop of mounting medium (Prolong Gold Antifade reagent with DAPI, Invitrogen). After solidification of the mounting medium, samples were sealed with nail polish and stored at 6 °C in the dark until further microscopic investigation.

Fluorescence measurements were performed on a modified Leica TCS SP8 CARS laser scanning microscope equipped with a 25 × water immersion objective (Fluotar VISIR 25x/0.95 WATER). BODIPY fluorescence was excited at 488 nm and detected with a hybrid detector (Leica HyD, specifications can be found on the manufacturer’s website) in the range 495–600 nm. DAPI fluorescence was excited at 405 nm and detected with a photomultiplier tube ranging from 415 to 475 nm. For each image, both fluorescence measurements were performed sequentially. Fluorescence data were acquired as small 3D stacks with a resolution of 2048 × 2048 pixel (pixel size 227 × 227 nm) in X → Y direction and 9—12 layers in Z direction with a step size of 570 nm.

Data processing was performed using Matlab R2015a. For better comparability, measurements were preprocessed as following: for each image stack, the mean intensity was calculated in Z direction. For the resulting 2D image, background noise was reduced by setting all data points with intensities lower than 1% to this background threshold. To account for cosmic spikes, an upper intensity threshold was determined. Consequently, less than 0.1% of all data points above background threshold showed an intensity value higher than this threshold. Intensities of data points exceeding this upper intensity threshold were set to this value. Afterwards, the image was rescaled to full range (8 bit) between the two threshold values.

From the processed mean images, single cell images were selected using a manually defined irregular octagon. For each of these single cell images, an intensity histogram analysis was generated whereby histogram values were normalized to percentage values (to account for the different sizes of the single cell images). The total fraction of data points showing 50% or more of the maximum intensity value was calculated for each single cell. At least 30 individual cells were evaluated per sample.

#### Muscle biopsy specimen

Cryosections for immunofluorescence studies were cut at 7 µm thickness, fixed in acetone for 5 min at − 20 °C and air dried either to be stained directly or stored at − 20 °C for subsequent staining, except of sections for anti-α-Dystroglycan staining, which were fixed for 1 min at − 20 °C in an 1:1 mixture of ethanol/glacial acetic acid and then washed in PBS, not dried.

Following primary antibodies were used: anti-α-B-Cyrstallin (polyclonal rabbit, 1:200; Stressgen, Canada), anti-α-Dystroglycan (monoclonal mouse, 1:75; VIA4-1; Merck Millipore, Germany), anti-NCAM1 (monoclonal mouse, 1:100; Abcam), anti-HSP90 (polyclonal rabbit, 1:100; Genetex), anti-Ataxin 2 (polyclonal rabbit, 1:50; Abcam), anti-Periostin (polycloncal rabbit, 1:100; Abcam), anti-Synpatopodin-2 (M2 polyconal rabbit, 1:300 and HH9 monoclonal mouse, 1:2; both kindly provided by Peter F.M. van der Ven/Dieter O. Fürst). Primary antibodies were diluted in PBS and applied overnight at 4 °C. Furthermore, fluorescence staining of Actin filaments was performed using the Phalloidin-iFluor 488 reagent (Abcam). Secondary antibodies used were Alexa 488 conjugated donkey anti-mouse IgG (1:400) and Alexa 555 conjugated donkey anti-rabbit IgG (1:800) (Molecular Probes, Invitrogen, Thermo Fisher Scientific, Germany). The secondary antibodies were diluted in 1×PBS and applied for one hour at room temperature. The sections were finally mounted in a Mowiol 4-88 (Calbiochem, Merck Chemicals, Germany) and glycerol mix in pH 8.5 Tris buffer with 0.1% DABCO (1,4-Diazabicyclo(2,2,2)octane; Sigma-Aldrich). Images were acquired with an AxioScope.A1 microscope using an Axiocam 503 color camera and ZEN 2.3 (blue edition) software (all Carl Zeiss Microscopy Ltd., Germany). Further image processing was performed with Adobe Photoshop CS6 Extended (Adobe Systems Inc., CA, USA).

Coherent anti-Stokes Raman scattering (CARS) and second harmonic generation (SHG) measurements were performed on a modified Leica TCS SP8 CARS with an APE picoEmerald laser system. Ten micrometer thick sections were cut from cryo tissue blocks. The samples were stored at -80 °C and thoroughly dried before measurements at room temperature. No further sample preparation was applied. The statistical evaluation is based on the 10 CARS images. A total of 103 muscle fibres were collected for the evaluation. For further details see Additional file 5.

## Supplementary Information

**Additional file 1: Table 1.** Catalogue of proteins expressed in human skin fibroblasts: List of all proteins identified within the spectral library based on the analysis of 45 pH8-fractionated samples. Fractions were analyzed on an Orbitrap Fusion Lumos Tribrid mass spectrometer.

**Additional file 2: Table 2.** Proteins of neuromuscular relevance expressed in human skin fibroblasts: Expression of 257 proteins is categorized according to different genetically caused diseased impacting on proper function of motoneurons, the peripheral nervous system, the neuromuscular junction, and the muscle fibre.

**Additional file 3: Table 3.** List of proteins affected by bi-allelic c.762delC *AAAS* mutation in whole protein extracts of human skin fibroblasts identified by global proteomic profiling: 228 proteins were found to be increased whereas 156 were decreased in the nuclear fractions of patient-derived cells. For each protein, the predicted function as well as the subcellular localization (www.uniprot.org) is provided.

**Additional file 4: Table 4.** List of proteins affected by bi-allelic c.762delC *AAAS* mutation in enriched nuclear fraction of human skin fibroblasts: ten proteins were found to be increased whereas nine were decreased in the nuclear fractions of patient-derived cells. For each protein, the predicted function as well as the subcellular localization (www.uniprot.org) is provided.

**Additional file 5. **Findings of Coherent anti-Stokes Raman scattering (CARS) and second harmonic generation (SHG) measurements on patient-derived muscle biopsy specimen.

## Data Availability

The spectral library data have been deposited to the ProteomeXchange Consortium via the PRIDE partner repository with the dataset identifier PXD019060. The proteomic profiling data have been deposited to the ProteomeXchange Consortium via the PRIDE partner repository with the dataset identifier PXD019201.
