# Supplementary material for: Protein signature of human skin fibroblasts allows the study of the molecular etiology of rare neurological diseases
Source: Orphanet J Rare Dis. 2021 Feb 9;16:73. doi: 10.1186/s13023-020-01669-1 (PMC7874489; doi:10.1186/s13023-020-01669-1)
Supplement: Supplementary file 3 — Additional file 3: Table 3. List of proteins affected by bi-allelic c.762delC AAAS mutation in whole protein extracts of human skin fibroblasts identified by global proteomic profiling: 228 proteins were found to be increased whereas 156 were decreased in the nuclear fractions of patient-derived cells. For each protein, the predicted function as well as the subcellular localization (www.uniprot.org) is provided. [file 13023_2020_1669_MOESM3_ESM.docx]

| **Accession** | **Protein name** | **Unique Peptides** | **Fold change** | **p-value** | **Subcellular localization** | **Involvement in disease** | **Cellular/ biological function (www.uniprot.org)** |
| --- | --- | --- | --- | --- | --- | --- | --- |
| O75832 | 26S proteasome non-ATPase regulatory subunit 10 | 12 | 2.38 | 0.00 | Cytoplasm |  | Acts as a chaperone during the assembly of the 26S proteasome, specifically of the PA700/19S regulatory complex (RC). In the initial step of the base subcomplex assembly is part of an intermediate PSMD10 |
| Q92552 | 28S ribosomal protein S27, mitochondrial | 13 | 2.35 | 0.01 | Cytoplasm |  | RNA-binding component of the mitochondrial small ribosomal subunit (mt-SSU) that plays a role in mitochondrial protein synthesis. |
| P08708 | 40S ribosomal protein S17 | 14 | 2.56 | 0.00 |  | Diamond-Blackfan anemia 4 (DBA4) |  |
| P63220 | 40S ribosomal protein S21 | 11 | 2.17 | 0.00 | Cytoplasm, cytosol |  |  |
| P62273 | 40S ribosomal protein S29 | 4 | 3.04 | 0.00 | Cytoplasm, cytosol | Diamond-Blackfan anemia 13 (DBA13) |  |
| Q96CB9 | 5-methylcytosine rRNA methyltransferase NSUN4 | 6 | 2.18 | 0.00 | Mitochondrion |  | Involved in mitochondrial ribosome assembly. 5-methylcytosine rRNA methyltransferase that probably is involved in mitochondrial ribosome small subunit (SSU) maturation by methylation of mitochondrial 12S rRNA; the function is independent of MTERFD2/MTERF4 and assembled mitochondrial ribosome large subunit (LSU). Targeted to LSU by MTERFD2/MTERF4 and probably is involved in a final step in ribosome biogenesis to ensure that SSU and LSU are assembled. In vitro can methylate 16S rRNA of the LSU; the methylation is enhanced by MTERFD/MTERF4. |
| P05386 | 60S acidic ribosomal protein P1 | 3 | 2.48 | 0.00 |  |  | Plays an important role in the elongation step of protein synthesis. |
| P27635 | 60S ribosomal protein L10 | 12 | 2.80 | 0.00 |  | Autism, X-linked 5 (AUTSX5) | Component of the large ribosomal subunit. |
| P62888 | 60S ribosomal protein L30 | 10 | 5.42 | 0.00 |  |  |  |
| P63261 | Actin, cytoplasmic 2 | 4 | 3.23 | 0.01 | Cytoplasm, cytoskeleton | Deafness, autosomal dominant, 20 (DFNA20) | Actins are highly conserved proteins that are involved in various types of cell motility and are ubiquitously expressed in all eukaryotic cells. |
| Q96PE1 | Adhesion G protein-coupled receptor A2 | 11 | 2.42 | 0.00 | Cell membrane |  | Endothelial receptor which functions together with RECK to enable brain endothelial cells to selectively respond to Wnt7 signals (WNT7A or WNT7B). |
| Q9NX46 | ADP-ribose glycohydrolase ARH3 | 9 | 2.08 | 0.00 | Nucleus | Neurodegeneration, childhood-onset, stress-induced, with variable ataxia and seizures (CONDSIAS) | ADP-ribose glycohydrolase that preferentially hydrolyzes the scissile alpha-O-linkage attached to the anomeric C1'' position of ADP-ribose and acts on different substrates, such as proteins ADP-ribosylated on serine, free poly(ADP-ribose) and O-acetyl-ADP-D-ribose. |
| Q10588 | ADP-ribosyl cyclase/cyclic ADP-ribose hydrolase 2 | 19 | 2.33 | 0.00 | Cell membrane; Lipid-anchor, GPI-anchor. |  | Synthesizes the second messengers cyclic ADP-ribose and nicotinate-adenine dinucleotide phosphate, the former a second messenger that elicits calcium release from intracellular stores. May be involved in pre-B-cell growth. |
| Q8N6T3 | ADP-ribosylation factor GTPase-activating protein 1 | 89 | 3.08 | 0.00 | Cytoplasm |  | GTPase-activating protein (GAP) for the ADP ribosylation factor 1 (ARF1). Involved in membrane trafficking and /or vesicle transport. Promotes hydrolysis of the ARF1-bound GTP and thus, is required for the dissociation of coat proteins from Golgi-derived membranes and vesicles, a prerequisite for vesicle's fusion with target compartment. Probably regulates ARF1-mediated transport via its interaction with the KDELR proteins and TMED2. Overexpression induces the redistribution of the entire Golgi complex to the endoplasmic reticulum, as when ARF1 is deactivated. Its activity is stimulated by phosphoinosides and inhibited by phosphatidylcholine. |
| Q02952 | A-kinase anchor protein 12 | 8 | 2.11 | 0.03 | Cytoplasm, cell cortex |  | Anchoring protein that mediates the subcellular compartmentation of protein kinase A (PKA) and protein kinase C (PKC). |
| Q8TD30 | Alanine aminotransferase 2 | 14 | 2.38 | 0.00 |  | Mental retardation, autosomal recessive 49 (MRT49) | Catalyzes the reversible transamination between alanine and 2-oxoglutarate to form pyruvate and glutamate. |
| P02511 | Alpha-crystallin B chain | 8 | 2.02 | 0.01 | Cytoplasm | Myopathy, myofibrillar, 2 (MFM2) | May contribute to the transparency and refractive index of the lens. Has chaperone-like activity, preventing aggregation of various proteins under a wide range of stress conditions. |
| Q12800 | Alpha-globin transcription factor CP2 | 17 | 2.08 | 0.01 | Nucleus |  | Binds a variety of cellular and viral promoters including fibrinogen, alpha-globin, SV40 and HIV-1 promoters. Activation of the alpha-globin promoter in erythroid cells is via synergistic interaction with UBP1 (By similarity). Functions as part of the SSP (stage selector protein) complex. Facilitates the interaction of the gamma-globin genes with enhancer elements contained in the locus control region in fetal erythroid cells. Interacts by binding to the stage selector element (SSE) in the proximal gamma-globin promoter. |
| Q9NVD7 | Alpha-parvin | 2 | 2.36 | 0.01 | Cell junction, focal adhesion. Cell membrane; Peripheral membrane protein; Cytoplasmic side. Cytoplasm, cytoskeleton. Cytoplasm, myofibril, sarcomere, Z line |  | Plays a role in sarcomere organization and in smooth muscle cell contraction. Required for normal development of the embryonic cardiovascular system, and for normal septation of the heart outflow tract. Plays a role in sprouting angiogenesis and is required for normal adhesion of vascular smooth muscle cells to endothelial cells during blood vessel development (By similarity). Plays a role in the reorganization of the actin cytoskeleton, formation of lamellipodia and ciliogenesis. Plays a role in the establishement of cell polarity, cell adhesion, cell spreading, and directed cell migration. |
| P27216 | Annexin A13 | 23 | 2.26 | 0.01 | Apical cell membrane |  |  |
| Q99700 | Ataxin-2 | 15 | 2.09 | 0.00 | Cytoplasm | Spinocerebellar ataxia 2 (SCA2) | Involved in EGFR trafficking, acting as negative regulator of endocytic EGFR internalization at the plasma membrane. |
| Q8WXF7 | Atlastin-1 | 2 | 7.26 | 0.00 | Endoplasmic reticulum membrane | Spastic paraplegia 3, autosomal dominant (SPG3) | GTPase tethering membranes through formation of trans-homooligomers and mediating homotypic fusion of endoplasmic reticulum membranes. Functions in endoplasmic reticulum tubular network biogenesis. |
| P03928 | ATP synthase protein 8 | 3 | 2.07 | 0.01 | Mitochondrion membrane; Single-pass membrane protein. | Mitochondrial complex V deficiency, mitochondrial 2 (MC5DM2) | Mitochondrial membrane ATP synthase (F(1)F(0) ATP synthase or Complex V) produces ATP from ADP in the presence of a proton gradient across the membrane which is generated by electron transport complexes of the respiratory chain. F-type ATPases consist of two structural domains, F(1) - containing the extramembraneous catalytic core and F(0) - containing the membrane proton channel, linked together by a central stalk and a peripheral stalk. During catalysis, ATP synthesis in the catalytic domain of F(1) is coupled via a rotary mechanism of the central stalk subunits to proton translocation. Part of the complex F(0) domain. Minor subunit located with subunit a in the membrane. |
| P30049 | ATP synthase subunit delta, mitochondrial | 6 | 2.26 | 0.00 | Mitochondrion. Mitochondrion inner membrane. | Mitochondrial complex V deficiency, nuclear type 5 (MC5DN5) | Mitochondrial membrane ATP synthase (F(1)F(0) ATP synthase or Complex V) produces ATP from ADP in the presence of a proton gradient across the membrane which is generated by electron transport complexes of the respiratory chain. |
| P33897 | ATP-binding cassette sub-family D member 1 | 21 | 2.05 | 0.00 | Peroxisome membrane | Adrenoleukodystrophy (ALD) | Plays a role in the transport of free very-long-chain fatty acids (VLCFAs) as well as their CoA-esters across the peroxisomal membrane by acting as an ATP-specific binding subunit releasing ADP after ATP hydrolysis. |
| Q8NFD5 | AT-rich interactive domain-containing protein 1B | 23 | 2.59 | 0.00 | Nucleus | Coffin-Siris syndrome 1 (CSS1) | Involved in transcriptional activation and repression of select genes by chromatin remodeling (alteration of DNA-nucleosome topology). Component of SWI/SNF chromatin remodeling complexes that carry out key enzymatic activities, changing chromatin structure by altering DNA-histone contacts within a nucleosome in an ATP-dependent manner. Belongs to the neural progenitors-specific chromatin remodeling complex (npBAF complex) and the neuron-specific chromatin remodeling complex (nBAF complex). During neural development a switch from a stem/progenitor to a postmitotic chromatin remodeling mechanism occurs as neurons exit the cell cycle and become committed to their adult state. The transition from proliferating neural stem/progenitor cells to postmitotic neurons requires a switch in subunit composition of the npBAF and nBAF complexes. As neural progenitors exit mitosis and differentiate into neurons, npBAF complexes which contain ACTL6A/BAF53A and PHF10/BAF45A, are exchanged for homologous alternative ACTL6B/BAF53B and DPF1/BAF45B or DPF3/BAF45C subunits in neuron-specific complexes (nBAF). The npBAF complex is essential for the self-renewal/proliferative capacity of the multipotent neural stem cells. The nBAF complex along with CREST plays a role regulating the activity of genes essential for dendrite growth (By similarity). Binds DNA non-specifically . |
| Q13884 | Beta-1-syntrophin | 5 | 2.69 | 0.00 | Cell membrane, sarcolemma |  | Adapter protein that binds to and probably organizes the subcellular localization of a variety of membrane proteins. May link various receptors to the actin cytoskeleton and the dystrophin glycoprotein complex. |
| Q9NUP1 | Biogenesis of lysosome-related organelles complex 1 subunit 4 | 8 | 2.10 | 0.01 | Cytoplasm |  | Component of the BLOC-1 complex, a complex that is required for normal biogenesis of lysosome-related organelles (LRO), such as platelet dense granules and melanosomes. In concert with the AP-3 complex, the BLOC-1 complex is required to target membrane protein cargos into vesicles assembled at cell bodies for delivery into neurites and nerve terminals. The BLOC-1 complex, in association with SNARE proteins, is also proposed to be involved in neurite extension. Plays a role in intracellular vesicle trafficking. |
| P50583 | Bis(5'-nucleosyl)-tetraphosphatase | 16 | 2.27 | 0.03 |  |  | Asymmetrically hydrolyzes Ap4A to yield AMP and ATP. Plays a major role in maintaining homeostasis. |
| Q6PJG6 | BRCA1-associated ATM activator 1 | 9 | 4.09 | 0.01 | Nucleus | Rigidity and multifocal seizure syndrome, lethal neonatal (RMFSL) | Involved in DNA damage response; activates kinases ATM, SMC1A and PRKDC by modulating their phosphorylation status following ionizing radiation (IR) stress. |
| P56945 | Breast cancer anti-estrogen resistance protein 1 | 7 | 2.15 | 0.00 | Cell junction, focal adhesion |  | Docking protein which plays a central coordinating role for tyrosine kinase-based signaling related to cell adhesion. Implicated in induction of cell migration. Overexpression confers antiestrogen resistance on breast cancer cells. |
| Q15059 | Bromodomain-containing protein 3 | 4 | 4.65 | 0.02 | Nucleus |  | Chromatin reader that recognizes and binds hyperacetylated chromatin and plays a role in the regulation of transcription, probably by chromatin remodeling and interaction with transcription factors. |
| Q8WUQ7 | Cactin | 9 | 3.35 | 0.01 | Nucleus |  | Involved in the regulation of innate immune response. |
| P63098 | Calcineurin subunit B type 1 | 7 | 2.91 | 0.01 | Cytoplasm, cytosol |  | Regulatory subunit of calcineurin, a calcium-dependent, calmodulin stimulated protein phosphatase. Confers calcium sensitivity. |
| Q9HA72 | Calcium homeostasis modulator protein 2 | 62 | 2.42 | 0.00 | Membrane |  | Pore-forming subunit of a voltage-gated ion channel. |
| Q05682 | Caldesmon | 22 | 2.48 | 0.00 | Cytoplasm, cytoskeleton |  | Actin- and myosin-binding protein implicated in the regulation of actomyosin interactions in smooth muscle and nonmuscle cells (could act as a bridge between myosin and actin filaments). Stimulates actin binding of tropomyosin which increases the stabilization of actin filament structure. In muscle tissues, inhibits the actomyosin ATPase by binding to F-actin. This inhibition is attenuated by calcium-calmodulin and is potentiated by tropomyosin. Interacts with actin, myosin, two molecules of tropomyosin and with calmodulin. Also plays an essential role during cellular mitosis and receptor capping. Involved in Schwann cell migration during peripheral nerve regeneration. |
| P04632 | Calpain small subunit 1 | 15 | 4.11 | 0.00 | Cytoplasm |  | Regulatory subunit of the calcium-regulated non-lysosomal thiol-protease which catalyzes limited proteolysis of substrates involved in cytoskeletal remodeling and signal transduction. |
| P51911 | Calponin-1 | 24 | 6.42 | 0.00 |  |  | Thin filament-associated protein that is implicated in the regulation and modulation of smooth muscle contraction. It is capable of binding to actin, calmodulin, troponin C and tropomyosin. The interaction of calponin with actin inhibits the actomyosin Mg-ATPase activity. |
| Q15417 | Calponin-3 | 25 | 3.59 | 0.01 |  |  | Thin filament-associated protein that is implicated in the regulation and modulation of smooth muscle contraction. It is capable of binding to actin, calmodulin, troponin C and tropomyosin. The interaction of calponin with actin inhibits the actomyosin Mg-ATPase activity. |
| P07858 | Cathepsin B | 7 | 2.03 | 0.01 | Lysosome | Keratolytic winter erythema (KWE) | Thiol protease which is believed to participate in intracellular degradation and turnover of proteins. |
| P29279 | CCN family member 2 | 7 | 2.58 | 0.04 | Secreted, extracellular space, extracellular matrix |  | Major connective tissue mitoattractant secreted by vascular endothelial cells. Promotes proliferation and differentiation of chondrocytes. Mediates heparin- and divalent cation-dependent cell adhesion in many cell types including fibroblasts, myofibroblasts, endothelial and epithelial cells. Enhances fibroblast growth factor-induced DNA synthesis. |
| P13987 | CD59 glycoprotein | 12 | 2.43 | 0.00 | Cell membrane; Lipid-anchor, GPI-anchor. Secreted. | Hemolytic anemia, CD59-mediated, with or without polyneuropathy (HACD59) | Potent inhibitor of the complement membrane attack complex (MAC) action. Acts by binding to the C8 and/or C9 complements of the assembling MAC, thereby preventing incorporation of the multiple copies of C9 required for complete formation of the osmolytic pore. This inhibitor appears to be species-specific. Involved in signal transduction for T-cell activation complexed to a protein tyrosine kinase. |
| P14209 | CD99 antigen | 5 | 2.74 | 0.01 | Membrane |  | Involved in T-cell adhesion processes and in spontaneous rosette formation with erythrocytes. Plays a role in a late step of leukocyte extravasation helping leukocytes to overcome the endothelial basement membrane. Acts at the same site as, but independently of, PECAM1. Involved in T-cell adhesion processes. |
| Q00587 | Cdc42 effector protein 1 | 7 | 3.23 | 0.02 | Endomembrane system |  | Probably involved in the organization of the actin cytoskeleton. Induced membrane extensions in fibroblasts. |
| O14613 | Cdc42 effector protein 2 | 5 | 2.12 | 0.01 | Endomembrane system |  | Probably involved in the organization of the actin cytoskeleton. May act downstream of CDC42 to induce actin filament assembly leading to cell shape changes. Induces pseudopodia formation in fibroblasts in a CDC42-dependent manner. |
| P62633 | Cellular nucleic acid-binding protein | 9 | 3.50 | 0.00 | Cytoplasm | Dystrophia myotonica 2 (DM2) | Single-stranded DNA-binding protein, with specificity to the sterol regulatory element (SRE). Involved in sterol-mediated repression. |
| Q9UFW8 | CGG triplet repeat-binding protein 1 | 20 | 3.88 | 0.00 | Nucleus |  | Binds to nonmethylated 5'-d(CGG)(n)-3' trinucleotide repeats in the FMR1 promoter. May play a role in regulating FMR1 promoter. |
| P51798 | Chloride channel 7 alpha subunit | 21 | 2.24 | 0.01 | Lysosome membrane | Osteopetrosis, autosomal recessive 4 (OPTB4) | Slowly voltage-gated channel mediating the exchange of chloride ions against protons. Functions as antiporter and contributes to the acidification of the lysosome lumen. |
| P83916 | Chromobox protein homolog 1 | 12 | 2.38 | 0.00 | Nucleus |  | Component of heterochromatin. Recognizes and binds histone H3 tails methylated at 'Lys-9', leading to epigenetic repression. Interaction with lamin B receptor (LBR) can contribute to the association of the heterochromatin with the inner nuclear membrane. |
| Q12873 | Chromodomain-helicase-DNA-binding protein 3 | 52 | 2.05 | 0.01 | Nucleus | Snijders Blok-Campeau syndrome (SNIBCPS) | Component of the histone deacetylase NuRD complex which participates in the remodeling of chromatin by deacetylating histones. Required for anchoring centrosomal pericentrin in both interphase and mitosis, for spindle organization and centrosome integrity. |
| O75122 | CLIP-associating protein 2 | 186 | 2.36 | 0.00 | Cytoplasm, cytoskeleton |  | Microtubule plus-end tracking protein that promotes the stabilization of dynamic microtubules. |
| P10909 | Clusterin | 46 | 2.34 | 0.01 |  |  |  |
| Q99715 | Collagen alpha-1 | 2 | 4.11 | 0.00 | Secreted, extracellular space, extracellular matrix | Ullrich congenital muscular dystrophy 2 (UCMD2) | Type XII collagen interacts with type I collagen-containing fibrils, the COL1 domain could be associated with the surface of the fibrils, and the COL2 and NC3 domains may be localized in the perifibrillar matrix. |
| P20908 | Collagen alpha-1 (V) chain | 21 | 4.37 | 0.00 | Secreted, extracellular space, extracellular matrix | Ehlers-Danlos syndrome, classic type, 1 (EDSCL1) | Type V collagen is a member of group I collagen (fibrillar forming collagen). It is a minor connective tissue component of nearly ubiquitous distribution. Type V collagen binds to DNA, heparan sulfate, thrombospondin, heparin, and insulin. |
| P05997 | Collagen alpha-2 (V) chain | 2 | 3.55 | 0.05 | Secreted, extracellular space, extracellular matrix | Ehlers-Danlos syndrome, classic type, 2 (EDSCL2) | Type V collagen is a member of group I collagen (fibrillar forming collagen). It is a minor connective tissue component of nearly ubiquitous distribution. Type V collagen binds to DNA, heparan sulfate, thrombospondin, heparin, and insulin. Type V collagen is a key determinant in the assembly of tissue-specific matrices. |
| Q9BXJ0 | Complement C1q tumor necrosis factor-related protein 5 | 4 | 18.45 | 0.00 | Secreted | Late-onset retinal degeneration (LORD) |  |
| Q9P1F3 | Costars family protein ABRACL | 9 | 3.84 | 0.03 |  |  |  |
| Q9NWM3 | CUE domain-containing protein 1 | 7 | 4.52 | 0.04 |  |  |  |
| O95319 | CUGBP Elav-like family member 2 | 12 | 3.72 | 0.00 | Nucleus. Cytoplasm. Note=Accumulates in the cytoplasm after ionizing radiation (By similarity). Colocalizes with APOBEC1 and A1CF. RNA-binding activity is detected in both nuclear and cytoplasmic compartments. |  | RNA-binding protein implicated in the regulation of several post-transcriptional events. Involved in pre-mRNA alternative splicing, mRNA translation and stability. Mediates exon inclusion and/or exclusion in pre-mRNA that are subject to tissue-specific and developmentally regulated alternative splicing. Specifically activates exon 5 inclusion of TNNT2 in embryonic, but not adult, skeletal muscle. Activates TNNT2 exon 5 inclusion by antagonizing the repressive effect of PTB. Acts as both an activator and repressor of a pair of coregulated exons. |
| Q96BA8 | Cyclic AMP-responsive element-binding protein 3-like protein 1 | 11 | 2.69 | 0.00 | Endoplasmic reticulum membrane | Osteogenesis imperfecta 16 (OI16) | Transcription factor involved in unfolded protein response (UPR). Binds the DNA consensus sequence 5'-GTGXGCXGC-3'. |
| P21291 | Cysteine and glycine-rich protein 1 | 8 | 2.21 | 0.01 | Nucleus |  | Could play a role in neuronal development. |
| Q16527 | Cysteine and glycine-rich protein 2 | 8 | 2.14 | 0.04 | Nucleus |  | Drastically down-regulated in response to PDGF-BB or cell injury, that promote smooth muscle cell proliferation and dedifferentiation. Seems to play a role in the development of the embryonic vascular system. |
| P20674 | Cytochrome c oxidase subunit 5A, mitochondrial | 19 | 2.36 | 0.00 | Mitochondrion inner membrane | Note=Mitochondrial complex IV deficiency is a rare condition caused by mutation in COX5A that lead to pulmonary arterial hypertension (PAH), failure to thrive and lactic acidemia. {ECO | Component of the cytochrome c oxidase, the last enzyme in the mitochondrial electron transport chain which drives oxidative phosphorylation. The respiratory chain contains 3 multisubunit complexes succinate dehydrogenase (complex II, CII), ubiquinol-cytochrome c oxidoreductase (cytochrome b-c1 complex, complex III, CIII) and cytochrome c oxidase (complex IV, CIV), that cooperate to transfer electrons derived from NADH and succinate to molecular oxygen, creating an electrochemical gradient over the inner membrane that drives transmembrane transport and the ATP synthase. Cytochrome c oxidase is the component of the respiratory chain that catalyzes the reduction of oxygen to water. Electrons originating from reduced cytochrome c in the intermembrane space (IMS) are transferred via the dinuclear copper A center (CU(A)) of subunit 2 and heme A of subunit 1 to the active site in subunit 1, a binuclear center (BNC) formed by heme A3 and copper B (CU(B)). The BNC reduces molecular oxygen to 2 water molecules using 4 electrons from cytochrome c in the IMS and 4 protons from the mitochondrial matrix. |
| P10606 | Cytochrome c oxidase subunit 5B, mitochondrial | 34 | 2.70 | 0.01 | Mitochondrion inner membrane |  | Component of the cytochrome c oxidase, the last enzyme in the mitochondrial electron transport chain which drives oxidative phosphorylation. The respiratory chain contains 3 multisubunit complexes succinate dehydrogenase (complex II, CII), ubiquinol-cytochrome c oxidoreductase (cytochrome b-c1 complex, complex III, CIII) and cytochrome c oxidase (complex IV, CIV), that cooperate to transfer electrons derived from NADH and succinate to molecular oxygen, creating an electrochemical gradient over the inner membrane that drives transmembrane transport and the ATP synthase. Cytochrome c oxidase is the component of the respiratory chain that catalyzes the reduction of oxygen to water. Electrons originating from reduced cytochrome c in the intermembrane space (IMS) are transferred via the dinuclear copper A center (CU(A)) of subunit 2 and heme A of subunit 1 to the active site in subunit 1, a binuclear center (BNC) formed by heme A3 and copper B (CU(B)). The BNC reduces molecular oxygen to 2 water molecules using 4 electrons from cytochrome c in the IMS and 4 protons from the mitochondrial matrix. |
| Q6UVY6 | DBH-like monooxygenase protein 1 | 20 | 14.22 | 0.05 | Endoplasmic reticulum membrane |  |  |
| Q9BTC0 | Death-inducer obliterator 1 | 5 | 2.02 | 0.00 | Cytoplasm |  | Putative transcription factor, weakly pro-apoptotic when overexpressed (By similarity). Tumor suppressor. Required for early embryonic stem cell development. |
| Q5JSL3 | Dedicator of cytokinesis protein 11 | 7 | 4.66 | 0.00 |  |  | Guanine nucleotide-exchange factor (GEF) that activates CDC42 by exchanging bound GDP for free GTP. Required for marginal zone (MZ) B-cell development, is associated with early bone marrow B-cell development, MZ B-cell formation, MZ B-cell number and marginal metallophilic macrophages morphology. Facilitates filopodia formation through the activation of CDC42. |
| P36954 | DNA-directed RNA polymerase II subunit RPB9 | 6 | 2.61 | 0.01 | Nucleus, nucleolus |  | DNA-dependent RNA polymerase catalyzes the transcription of DNA into RNA using the four ribonucleoside triphosphates as substrates. Component of RNA polymerase II which synthesizes mRNA precursors and many functional non-coding RNAs. Pol II is the central component of the basal RNA polymerase II transcription machinery. It is composed of mobile elements that move relative to each other. RPB9 is part of the upper jaw surrounding the central large cleft and thought to grab the incoming DNA template. |
| Q9H1X3 | DnaJ homolog subfamily C member 25 | 2 | 2.27 | 0.00 | Membrane |  |  |
| Q9H3Z4 | DnaJ homolog subfamily C member 5 | 6 | 3.47 | 0.00 | Membrane | Ceroid lipofuscinosis, neuronal, 4B (CLN4B) | Acts as a general chaperone in regulated exocytosis (By similarity). Acts as a co-chaperone for the SNARE protein SNAP-25 (By similarity). Involved in the calcium-mediated control of a late stage of exocytosis (By similarity). May have an important role in presynaptic function. May be involved in calcium-dependent neurotransmitter release at nerve endings. |
| Q9P2X0 | Dolichol-phosphate mannosyltransferase subunit 3 | 19 | 2.27 | 0.00 | Endoplasmic reticulum membrane; Multi-pass membrane protein. | Muscular dystrophy-dystroglycanopathy limb-girdle C15 (MDDGC15) | Stabilizer subunit of the dolichol-phosphate mannose (DPM) synthase complex; tethers catalytic subunit DPM1 to the ER. |
| P63167 | Dynein light chain 1, cytoplasmic | 15 | 2.78 | 0.00 | Cytoplasm, cytoskeleton, microtubule organizing center, centrosome |  | Acts as one of several non-catalytic accessory components of the cytoplasmic dynein 1 complex that are thought to be involved in linking dynein to cargos and to adapter proteins that regulate dynein function. Cytoplasmic dynein 1 acts as a motor for the intracellular retrograde motility of vesicles and organelles along microtubules. May play a role in changing or maintaining the spatial distribution of cytoskeletal structures. |
| Q14118 | Dystroglycan | 7 | 3.22 | 0.01 | [Alpha-dystroglycan] | Muscular dystrophy-dystroglycanopathy limb-girdle C9 (MDDGC9) | The dystroglycan complex is involved in a number of processes including laminin and basement membrane assembly, sarcolemmal stability, cell survival, peripheral nerve myelination, nodal structure, cell migration, and epithelial polarization. |
| Q7L5Y9 | E3 ubiquitin-protein transferase MAEA | 3 | 2.16 | 0.01 | Cytoplasm |  | Core component of the CTLH E3 ubiquitin-protein ligase complex that selectively accepts ubiquitin from UBE2H and mediates ubiquitination and subsequent proteasomal degradation of the transcription factor HBP1. MAEA and RMND5A are both required for catalytic activity of the CTLH E3 ubiquitin-protein ligase complex. |
| Q12805 | EGF-containing fibulin-like extracellular matrix protein 1 | 6 | 2.20 | 0.03 | Secreted, extracellular space | Doyne honeycomb retinal dystrophy (DHRD) | Binds EGFR, the EGF receptor, inducing EGFR autophosphorylation and the activation of downstream signaling pathways. May play a role in cell adhesion and migration. May function as a negative regulator of chondrocyte differentiation. In the olfactory epithelium, it may regulate glial cell migration, differentiation and the ability of glial cells to support neuronal neurite outgrowth. |
| Q9BV79 | Enoyl-[acyl-carrier-protein] reductase, mitochondrial | 27 | 2.23 | 0.00 |  | Dystonia, childhood-onset, with optic atrophy and basal ganglia abnormalities (DYTOABG) | Catalyzes the NADPH-dependent reduction of trans-2-enoyl thioesters in mitochondrial fatty acid synthesis (fatty acid synthesis type II). Fatty acid chain elongation in mitochondria uses acyl carrier protein (ACP) as an acyl group carrier, but the enzyme accepts both ACP and CoA thioesters as substrates in vitro. Has a preference for short and medium chain substrates, including trans-2-hexenoyl-CoA (C6), trans-2-decenoyl-CoA (C10), and trans-2-hexadecenoyl-CoA (C16). |
| Q13541 | Eukaryotic translation initiation factor 4E-binding protein 1 | 14 | 5.55 | 0.00 |  |  | Repressor of translation initiation that regulates EIF4E activity by preventing its assembly into the eIF4F complex. |
| Q9Y5Q0 | Fatty acid desaturase 3 | 6 | 7.35 | 0.01 | Endoplasmic reticulum membrane |  | Acts as a methyl-end fatty acyl coenzyme A (CoA) desaturase that introduces a cis double bond between the preexisting double bond and the terminal methyl group of the fatty acyl chain. Desaturates (11E)-octadecenoate (trans-vaccenoate) at carbon 13 to generate (11E,13Z)-octadecadienoate, likely participating in the biohydrogenation pathway of linoleic acid (LA). |
| Q9Y4F1 | FERM, ARHGEF and pleckstrin domain-containing protein 1 | 11 | 2.18 | 0.00 | Cell membrane; Peripheral membrane protein; Cytoplasmic side. Cell junction, synapse. Cell junction, synapse, synaptosome |  | Functions as guanine nucleotide exchange factor for RAC1. May play a role in semaphorin signaling. Plays a role in the assembly and disassembly of dendritic filopodia, the formation of dendritic spines, regulation of dendrite length and ultimately the formation of synapses. |
| O94887 | FERM, ARHGEF and pleckstrin domain-containing protein 2 | 22 | 2.24 | 0.00 |  |  | Functions as guanine nucleotide exchange factor that activates RAC1. May have relatively low activity. Plays a role in the response to class 3 semaphorins and remodeling of the actin cytoskeleton. Plays a role in TNFSF11-mediated osteoclast differentiation, especially in podosome rearrangement and reorganization of the actin cytoskeleton. Regulates the activation of ITGB3, integrin signaling and cell adhesion. |
| P02675 | Fibrinogen beta chain [Cleaved into: Fibrinopeptide B; Fibrinogen beta chain] | 2 | 2.63 | 0.01 | Secreted | Congenital afibrinogenemia (CAFBN) | Cleaved by the protease thrombin to yield monomers which, together with fibrinogen alpha (FGA) and fibrinogen gamma (FGG), polymerize to form an insoluble fibrin matrix. Fibrin has a major function in hemostasis as one of the primary components of blood clots. In addition, functions during the early stages of wound repair to stabilize the lesion and guide cell migration during re-epithelialization. Was originally thought to be essential for platelet aggregation, based on in vitro studies using anticoagulated blood. However subsequent studies have shown that it is not absolutely required for thrombus formation in vivo. Enhances expression of SELP in activated platelets. Maternal fibrinogen is essential for successful pregnancy. Fibrin deposition is also associated with infection, where it protects against IFNG-mediated hemorrhage. May also facilitate the antibacterial immune response via both innate and T-cell mediated pathways. |
| Q8WUP2 | Filamin-binding LIM protein 1 | 15 | 4.32 | 0.00 | Cell junction, focal adhesion |  | Serves as an anchoring site for cell-ECM adhesion proteins and filamin-containing actin filaments. Is implicated in cell shape modulation (spreading) and motility. May participate in the regulation of filamin-mediated cross-linking and stabilization of actin filaments. May also regulate the assembly of filamin-containing signaling complexes that control actin assembly. Promotes dissociation of FLNA from ITGB3 and ITGB7. Promotes activation of integrins and regulates integrin-mediated cell-cell adhesion. |
| P51116 | Fragile X mental retardation syndrome-related protein 2 | 26 | 3.05 | 0.00 | Cytoplasm. |  | RNA-binding protein. |
| Q9H840 | Gem-associated protein 7 | 11 | 2.18 | 0.00 | Nucleus, nucleoplasm |  | The SMN complex plays a catalyst role in the assembly of small nuclear ribonucleoproteins (snRNPs), the building blocks of the spliceosome. Thereby, plays an important role in the splicing of cellular pre-mRNAs. Most spliceosomal snRNPs contain a common set of Sm proteins SNRPB, SNRPD1, SNRPD2, SNRPD3, SNRPE, SNRPF and SNRPG that assemble in a heptameric protein ring on the Sm site of the small nuclear RNA to form the core snRNP. In the cytosol, the Sm proteins SNRPD1, SNRPD2, SNRPE, SNRPF and SNRPG are trapped in an inactive 6S pICln-Sm complex by the chaperone CLNS1A that controls the assembly of the core snRNP. Dissociation by the SMN complex of CLNS1A from the trapped Sm proteins and their transfer to an SMN-Sm complex triggers the assembly of core snRNPs and their transport to the nucleus. |
| Q9UKN8 | General transcription factor 3C polypeptide 4 | 20 | 2.09 | 0.00 | Nucleus. |  | Essential for RNA polymerase III to make a number of small nuclear and cytoplasmic RNAs, including 5S RNA, tRNA, and adenovirus-associated (VA) RNA of both cellular and viral origin. Has histone acetyltransferase activity (HAT) with unique specificity for free and nucleosomal H3. May cooperate with GTF3C5 in facilitating the recruitment of TFIIIB and RNA polymerase through direct interactions with BRF1, POLR3C and POLR3F. May be localized close to the A box. |
| P07093 | Glia-derived nexin | 5 | 2.90 | 0.00 | Secreted, extracellular space. |  | Serine protease inhibitor with activity toward thrombin, trypsin, and urokinase. Promotes neurite extension by inhibiting thrombin. Binds heparin. |
| Q96EK6 | Glucosamine 6-phosphate N-acetyltransferase | 2 | 3.43 | 0.01 | Golgi apparatus membrane; Peripheral membrane protein. Endosome membrane |  |  |
| P09211 | Glutathione S-transferase P | 8 | 5.10 | 0.00 | Cytoplasm |  | Conjugation of reduced glutathione to a wide number of exogenous and endogenous hydrophobic electrophiles. Regulates negatively CDK5 activity via p25/p35 translocation to prevent neurodegeneration. |
| P23434 | Glycine cleavage system H protein, mitochondrial | 8 | 6.45 | 0.00 | Mitochondrion | Non-ketotic hyperglycinemia (NKH) | The glycine cleavage system catalyzes the degradation of glycine. The H protein (GCSH) shuttles the methylamine group of glycine from the P protein (GLDC) to the T protein (GCST). |
| P62873 | Guanine nucleotide-binding protein G | 10 | 2.74 | 0.04 |  | Mental retardation, autosomal dominant 42 (MRD42) | Guanine nucleotide-binding proteins (G proteins) are involved as a modulator or transducer in various transmembrane signaling systems. The beta and gamma chains are required for the GTPase activity, for replacement of GDP by GTP, and for G protein-effector interaction. |
| P62879 | Guanine nucleotide-binding protein G | 10 | 2.32 | 0.03 | Cytoplasm, perinuclear region |  | Guanine nucleotide-binding proteins (G proteins) are involved as a modulator or transducer in various transmembrane signaling systems. The beta and gamma chains are required for the GTPase activity, for replacement of GDP by GTP, and for G protein-effector interaction. |
| P63218 | Guanine nucleotide-binding protein G | 8 | 2.74 | 0.05 | Cell membrane |  | Guanine nucleotide-binding proteins (G proteins) are involved as a modulator or transducer in various transmembrane signaling systems. The beta and gamma chains are required for the GTPase activity, for replacement of GDP by GTP, and for G protein-effector interaction. |
| Q9HAV0 | Guanine nucleotide-binding protein subunit beta-4 | 10 | 3.39 | 0.00 |  | Charcot-Marie-Tooth disease, dominant, intermediate type, F (CMTDIF) | Guanine nucleotide-binding proteins (G proteins) are involved as a modulator or transducer in various transmembrane signaling systems. The beta and gamma chains are required for the GTPase activity, for replacement of GDP by GTP, and for G protein-effector interaction. |
| Q92574 | Hamartin | 5 | 2.59 | 0.00 | Cytoplasm | Tuberous sclerosis 1 (TSC1) | In complex with TSC2, inhibits the nutrient-mediated or growth factor-stimulated phosphorylation of S6K1 and EIF4EBP1 by negatively regulating mTORC1 signaling. |
| O14558 | Heat shock protein beta-6 | 6 | 2.31 | 0.01 | Cytoplasm |  | Small heat shock protein which functions as a molecular chaperone probably maintaining denatured proteins in a folding-competent state. Seems to have versatile functions in various biological processes. Plays a role in regulating muscle function such as smooth muscle vasorelaxation and cardiac myocyte contractility. May regulate myocardial angiogenesis implicating KDR. Overexpression mediates cardioprotection and angiogenesis after induced damage. Stabilizes monomeric YWHAZ thereby supporting YWHAZ chaperone-like activity. |
| P52926 | High mobility group protein HMGI-C | 11 | 3.38 | 0.02 | Nucleus. | Note=A chromosomal aberration involving HMGA2 is associated with a subclass of benign mesenchymal tumors known as lipomas. Translocation t(3;12)(q27-q28;q13-q15) with LPP is shown in lipomas. HMGA2 is also fused with a number of other genes in lipomas. {ECO | Functions as a transcriptional regulator. Functions in cell cycle regulation through CCNA2. Plays an important role in chromosome condensation during the meiotic G2/M transition of spermatocytes. Plays a role in postnatal myogenesis, is involved in satellite cell activation. |
| Q9BX68 | Histidine triad nucleotide-binding protein 2, mitochondrial | 3 | 2.15 | 0.00 | Mitochondrion |  | Hydrolase probably involved in steroid biosynthesis. May play a role in apoptosis. Has adenosine phosphoramidase activity. |
| Q7L3B6 | Hsp90 co-chaperone Cdc37-like 1 | 21 | 3.32 | 0.00 | Cytoplasm |  | Co-chaperone that binds to numerous proteins and promotes their interaction with Hsp70 and Hsp90. |
| Q9Y5U9 | Immediate early response 3-interacting protein 1 | 14 | 4.90 | 0.00 | Endoplasmic reticulum membrane | Microcephaly, epilepsy, and diabetes syndrome (MEDS) | May be implicated in the regulation of apoptosis. May be involved in protein transport between endoplasmic reticulum and Golgi apparatus. |
| O00425 | Insulin-like growth factor 2 mRNA-binding protein 3 | 7 | 7.06 | 0.00 | Nucleus. Cytoplasm. |  | RNA-binding factor that may recruit target transcripts to cytoplasmic protein-RNA complexes (mRNPs). This transcript 'caging' into mRNPs allows mRNA transport and transient storage. It also modulates the rate and location at which target transcripts encounter the translational apparatus and shields them from endonuclease attacks or microRNA-mediated degradation. Binds to the 3'-UTR of CD44 mRNA and stabilizes it, hence promotes cell adhesion and invadopodia formation in cancer cells. Binds to beta-actin/ACTB and MYC transcripts. Binds to the 5'-UTR of the insulin-like growth factor 2 (IGF2) mRNAs. |
| Q16270 | Insulin-like growth factor-binding protein 7 | 12 | 3.11 | 0.04 | Secreted. | Retinal arterial macroaneurysm with supravalvular pulmonic stenosis (RAMSVPS) | Binds IGF-I and IGF-II with a relatively low affinity. Stimulates prostacyclin (PGI2) production. Stimulates cell adhesion. |
| P05362 | Intercellular adhesion molecule 1 | 2 | 2.96 | 0.00 | Membrane; Single-pass type I membrane protein. |  | ICAM proteins are ligands for the leukocyte adhesion protein LFA-1 (integrin alpha-L/beta-2). During leukocyte trans-endothelial migration, ICAM1 engagement promotes the assembly of endothelial apical cups through ARHGEF26/SGEF and RHOG activation. |
| O14879 | Interferon-induced protein with tetratricopeptide repeats 3 | 10 | 2.27 | 0.02 | Cytoplasm. Mitochondrion. |  | IFN-induced antiviral protein which acts as an inhibitor of cellular as well as viral processes, cell migration, proliferation, signaling, and viral replication. Enhances MAVS-mediated host antiviral responses by serving as an adapter bridging TBK1 to MAVS which leads to the activation of TBK1 and phosphorylation of IRF3 and phosphorylated IRF3 translocates into nucleus to promote antiviral gene transcription. Exihibits an antiproliferative activity via the up-regulation of cell cycle negative regulators CDKN1A/p21 and CDKN1B/p27. Normally, CDKN1B/p27 turnover is regulated by COPS5, which binds CDKN1B/p27 in the nucleus and exports it to the cytoplasm for ubiquitin-dependent degradation. IFIT3 sequesters COPS5 in the cytoplasm, thereby increasing nuclear CDKN1B/p27 protein levels. Upregulates CDKN1A/p21 by downregulating MYC, a repressor of CDKN1A/p21. Can negatively regulate the apoptotic effects of IFIT2. |
| Q01629 | Interferon-induced transmembrane protein 2 | 10 | 2.39 | 0.04 | Cell membrane |  | IFN-induced antiviral protein which inhibits the entry of viruses to the host cell cytoplasm, permitting endocytosis, but preventing subsequent viral fusion and release of viral contents into the cytosol. Active against multiple viruses, including influenza A virus, SARS coronavirus (SARS-CoV), Marburg virus (MARV), Ebola virus (EBOV), Dengue virus (DNV), West Nile virus (WNV), human immunodeficiency virus type 1 (HIV-1) and vesicular stomatitis virus (VSV). |
| Q5JVS0 | Intracellular hyaluronan-binding protein 4 | 34 | 2.19 | 0.00 | Nucleus |  | RNA-binding protein that plays a role in the regulation of transcription, pre-mRNA splicing and mRNA translation. |
| P51553 | Isocitrate dehydrogenase [NAD] subunit gamma, mitochondrial | 14 | 2.05 | 0.01 | Mitochondrion |  | Regulatory subunit which plays a role in the allosteric regulation of the enzyme catalyzing the decarboxylation of isocitrate (ICT) into alpha-ketoglutarate. The heterodimer composed of the alpha (IDH3A) and beta (IDH3B) subunits and the heterodimer composed of the alpha (IDH3A) and gamma (IDH3G) subunits, have considerable basal activity but the full activity of the heterotetramer (containing two subunits of IDH3A, one of IDH3B and one of IDH3G) requires the assembly and cooperative function of both heterodimers. |
| Q9P266 | Junctional protein associated with coronary artery disease | 9 | 3.87 | 0.00 | Cell junction, adherens junction |  |  |
| Q9H0B6 | Kinesin light chain 2 | 40 | 2.20 | 0.01 | Cytoplasm, cytoskeleton | Spastic paraplegia, optic atrophy, and neuropathy (SPOAN) | Kinesin is a microtubule-associated force-producing protein that may play a role in organelle transport. The light chain may function in coupling of cargo to the heavy chain or in the modulation of its ATPase activity. |
| Q9UFC0 | Leucine-rich repeat and WD repeat-containing protein 1 | 45 | 2.26 | 0.00 | Nucleus. Chromosome, centromere. Chromosome, telomere. Cytoplasm, cytoskeleton, microtubule organizing center, centrosome |  | Required for G1/S transition. Recruits and stabilizes the origin recognition complex (ORC) onto chromatin during G1 to establish pre-replication complex (preRC) and to heterochromatic sites in post-replicated cells. Binds a combination of DNA and histone methylation repressive marks on heterochromatin. Binds histone H3 and H4 trimethylation marks H3K9me3, H3K27me3 and H4K20me3 in a cooperative manner with DNA methylation. Required for silencing of major satellite repeats. May be important ORC2, ORC3 and ORC4 stability. |
| Q9UPQ0 | LIM and calponin homology domains-containing protein 1 | 30 | 2.07 | 0.01 | Cytoplasm, cytoskeleton, stress fiber |  | Actin stress fibers-associated protein that activates non-muscle myosin IIa. Activates the non-muscle myosin IIa complex by promoting the phosphorylation of its regulatory subunit MRLC/MYL9. Through the activation of non-muscle myosin IIa, positively regulates actin stress fibers assembly and stabilizes focal adhesions. It therefore negatively regulates cell spreading and cell migration. |
| Q9UHB6 | LIM domain and actin-binding protein 1 | 12 | 3.81 | 0.04 | Cytoplasm. Cell junction, focal adhesion |  | Actin-binding protein involved in actin cytoskeleton regulation and dynamics. Increases the number and size of actin stress fibers and inhibits membrane ruffling. Inhibits actin filament depolymerization. Bundles actin filaments, delays filament nucleation and reduces formation of branched filaments. |
| Q93052 | Lipoma-preferred partner | 23 | 2.47 | 0.00 | Nucleus. Cytoplasm. Cell junction. Cell membrane. | Note=A chromosomal aberration involving LPP is associated with a subclass of benign mesenchymal tumors known as lipomas. Translocation t(3;12)(q27-q28;q13-q15) with HMGA2 is shown in lipomas.; DISEASE | May play a structural role at sites of cell adhesion in maintaining cell shape and motility. In addition to these structural functions, it may also be implicated in signaling events and activation of gene transcription. May be involved in signal transduction from cell adhesion sites to the nucleus allowing successful integration of signals arising from soluble factors and cell-cell adhesion sites. Also suggested to serve as a scaffold protein upon which distinct protein complexes are assembled in the cytoplasm and in the nucleus. |
| Q7L5N7 | Lysophosphatidylcholine acyltransferase 2 | 6 | 2.05 | 0.00 | Endoplasmic reticulum membrane |  | Possesses both acyltransferase and acetyltransferase activities. Activity is calcium-dependent. Involved in platelet-activating factor (PAF) biosynthesis by catalyzing the conversion of the PAF precursor, 1-O-alkyl-sn-glycero-3-phosphocholine (lyso-PAF) into 1-O-alkyl-2-acetyl-sn-glycero-3-phosphocholine (PAF). Also converts lyso-PAF to 1-O-alkyl-2-acyl-sn-glycero-3-phosphocholine (PC), a major component of cell membranes and a PAF precursor. Under resting conditions, acyltransferase activity is preferred. Upon acute inflammatory stimulus, acetyltransferase activity is enhanced and PAF synthesis increases. Also catalyzes the conversion of 1-acyl-sn-glycero-3-phosphocholine to 1,2-diacyl-sn-glycero-3-phosphocholine. |
| P10253 | Lysosomal alpha-glucosidase | 7 | 2.49 | 0.00 | Lysosome | Glycogen storage disease 2 (GSD2) | Essential for the degradation of glycogen in lysosomes. |
| P14174 | Macrophage migration inhibitory factor | 2 | 2.16 | 0.01 | Secreted | Rheumatoid arthritis systemic juvenile (RASJ) | Pro-inflammatory cytokine. Involved in the innate immune response to bacterial pathogens. The expression of MIF at sites of inflammation suggests a role as mediator in regulating the function of macrophages in host defense. Counteracts the anti-inflammatory activity of glucocorticoids. Has phenylpyruvate tautomerase and dopachrome tautomerase activity (in vitro), but the physiological substrate is not known. It is not clear whether the tautomerase activity has any physiological relevance, and whether it is important for cytokine activity. |
| O75352 | Mannose-P-dolichol utilization defect 1 protein | 6 | 6.96 | 0.00 | Membrane | Congenital disorder of glycosylation 1F (CDG1F) | Required for normal utilization of mannose-dolichol phosphate (Dol-P-Man) in the synthesis of N-linked and O-linked oligosaccharides and GPI anchors. |
| P49006 | MARCKS-related protein | 8 | 2.20 | 0.03 | Cytoplasm, cytoskeleton |  | Controls cell movement by regulating actin cytoskeleton homeostasis and filopodium and lamellipodium formation. |
| Q9UIS9 | Methyl-CpG-binding domain protein 1 | 17 | 2.60 | 0.00 | Nucleus |  | Transcriptional repressor that binds CpG islands in promoters where the DNA is methylated at position 5 of cytosine within CpG dinucleotides. Binding is abolished by the presence of 7-mG that is produced by DNA damage by methylmethanesulfonate (MMS). Acts as transcriptional repressor and plays a role in gene silencing by recruiting AFT7IP, which in turn recruits factors such as the histone methyltransferase SETDB1. Probably forms a complex with SETDB1 and ATF7IP that represses transcription and couples DNA methylation and histone 'Lys-9' trimethylation. Isoform 1 and isoform 2 can also repress transcription from unmethylated promoters. |
| Q8N3F8 | MICAL-like protein 1 | 12 | 2.20 | 0.05 | Recycling endosome membrane; Peripheral membrane protein. Late endosome membrane. |  | Probable lipid-binding protein with higher affinity for phosphatidic acid, a lipid enriched in recycling endosome membranes. On endosome membranes, may act as a downstream effector of Rab proteins recruiting cytosolic proteins to regulate membrane tubulation. May be involved in a late step of receptor-mediated endocytosis regulating for instance endocytosed-EGF receptor trafficking. Alternatively, may regulate slow endocytic recycling of endocytosed proteins back to the plasma membrane. May indirectly play a role in neurite outgrowth. |
| O43772 | Mitochondrial carnitine/acylcarnitine carrier protein | 3 | 2.30 | 0.00 | Mitochondrion inner membrane; Multi-pass membrane protein. | Carnitine-acylcarnitine translocase deficiency (CACTD) | Mediates the transport of acylcarnitines of different length across the mitochondrial inner membrane from the cytosol to the mitochondrial matrix for their oxidation by the mitochondrial fatty acid-oxidation pathway. |
| Q9BSF4 | Mitochondrial import inner membrane translocase subunit Tim29 | 9 | 3.09 | 0.00 | Mitochondrion inner membrane |  | Component of the TIM22 complex, a complex that mediates the import and insertion of multi-pass transmembrane proteins into the mitochondrial inner membrane. The TIM22 complex forms a twin-pore translocase that uses the membrane potential as the external driving force. Required for the stability of the TIM22 complex and functions in the assembly of the TIMM22 protein into the TIM22 complex. May facilitate cooperation between TIM22 and TOM complexes by interacting with TOMM40. |
| Q9NS69 | Mitochondrial import receptor subunit TOM22 homolog | 3 | 2.24 | 0.05 | Mitochondrion outer membrane |  | Central receptor component of the translocase of the outer membrane of mitochondria (TOM complex) responsible for the recognition and translocation of cytosolically synthesized mitochondrial preproteins. Together with the peripheral receptor TOM20 functions as the transit peptide receptor and facilitates the movement of preproteins into the translocation pore. |
| Q70IA6 | MOB kinase activator 2 | 5 | 2.76 | 0.00 | Nucleus |  | Stimulates the autophosphorylation and kinase activity of STK38 and STK38L. |
| O60669 | Monocarboxylate transporter 2 | 3 | 2.46 | 0.01 | Cell membrane |  | Proton-coupled monocarboxylate transporter. Catalyzes the rapid transport across the plasma membrane of many monocarboxylates such as lactate, pyruvate, branched-chain oxo acids derived from leucine, valine and isoleucine, and the ketone bodies acetoacetate, beta-hydroxybutyrate and acetate. Functions as high-affinity pyruvate transporter. |
| Q8NI22 | Multiple coagulation factor deficiency protein 2 | 5 | 2.03 | 0.00 | Endoplasmic reticulum-Golgi intermediate compartment | Factor V and factor VIII combined deficiency 2 (F5F8D2) | The MCFD2-LMAN1 complex forms a specific cargo receptor for the ER-to-Golgi transport of selected proteins. Plays a role in the secretion of coagulation factors. |
| O95297 | Myelin protein zero-like protein 1 | 4 | 2.04 | 0.03 | Membrane |  | Cell surface receptor, which is involved in signal transduction processes. Recruits PTPN11/SHP-2 to the cell membrane and is a putative substrate of PTPN11/SHP-2. Is a major receptor for concanavalin-A (ConA) and is involved in cellular signaling induced by ConA, which probably includes Src family tyrosine-protein kinases. Isoform 3 seems to have a dominant negative role; it blocks tyrosine phosphorylation of MPZL1 induced by ConA. Isoform 1, but not isoform 2 and isoform 3, may be involved in regulation of integrin-mediated cell motility. |
| Q14814 | Myocyte-specific enhancer factor 2D | 17 | 2.04 | 0.03 | Nucleus |  | Transcriptional activator which binds specifically to the MEF2 element, 5'-YTA[AT](4)TAR-3', found in numerous muscle-specific, growth factor- and stress-induced genes. Mediates cellular functions not only in skeletal and cardiac muscle development, but also in neuronal differentiation and survival. Plays diverse roles in the control of cell growth, survival and apoptosis via p38 MAPK signaling in muscle-specific and/or growth factor-related transcription. Plays a critical role in the regulation of neuronal apoptosis. |
| P29966 | Myristoylated alanine-rich C-kinase substrate | 4 | 2.36 | 0.01 | Cytoplasm, cytoskeleton |  | MARCKS is the most prominent cellular substrate for protein kinase C. This protein binds calmodulin, actin, and synapsin. MARCKS is a filamentous (F) actin cross-linking protein. |
| Q9P032 | NADH dehydrogenase [ubiquinone] 1 alpha subcomplex assembly factor 4 | 8 | 2.16 | 0.02 | Mitochondrion | Mitochondrial complex I deficiency, nuclear type 15 (MC1DN15) | Involved in the assembly of mitochondrial NADH |
| O75380 | NADH dehydrogenase [ubiquinone] iron-sulfur protein 6, mitochondrial | 57 | 2.64 | 0.00 | Mitochondrion inner membrane | Mitochondrial complex I deficiency, nuclear type 9 (MC1DN9) | Accessory subunit of the mitochondrial membrane respiratory chain NADH dehydrogenase (Complex I), that is believed not to be involved in catalysis. Complex I functions in the transfer of electrons from NADH to the respiratory chain. The immediate electron acceptor for the enzyme is believed to be ubiquinone. |
| P08473 | Neprilysin | 5 | 3.15 | 0.04 | Cell membrane; Single-pass type II membrane protein. | Charcot-Marie-Tooth disease 2T (CMT2T) | Thermolysin-like specificity, but is almost confined on acting on polypeptides of up to 30 amino acids. |
| P62166 | Neuronal calcium sensor 1 | 9 | 2.10 | 0.00 | Golgi apparatus |  | Neuronal calcium sensor, regulator of G protein-coupled receptor phosphorylation in a calcium dependent manner. Directly regulates GRK1 (RHOK), but not GRK2 to GRK5. Can substitute for calmodulin (By similarity). Stimulates PI4KB kinase activity (By similarity). Involved in long-term synaptic plasticity through its interaction with PICK1 (By similarity). May also play a role in neuron differentiation through inhibition of the activity of N-type voltage-gated calcium channel. |
| P61916 | NPC intracellular cholesterol transporter 2 | 6 | 4.93 | 0.01 | Secreted | Niemann-Pick disease C2 (NPC2) | Intracellular cholesterol transporter which acts in concert with NPC1 and plays an important role in the egress of cholesterol from the lysosomal compartment. |
| Q96F24 | Nuclear receptor-binding factor 2 | 3 | 3.03 | 0.00 | Nucleus |  | May modulate transcriptional activation by target nuclear receptors. Can act as transcriptional activator (in vitro). |
| Q13952 | Nuclear transcription factor Y subunit gamma | 10 | 2.21 | 0.02 | Nucleus. |  | Component of the sequence-specific heterotrimeric transcription factor (NF-Y) which specifically recognizes a 5'-CCAAT-3' box motif found in the promoters of its target genes. NF-Y can function as both an activator and a repressor, depending on its interacting cofactors. |
| Q9H1E3 | Nuclear ubiquitous casein and cyclin-dependent kinase substrate 1 | 2 | 6.70 | 0.00 | Nucleus. |  |  |
| P22392 | Nucleoside diphosphate kinase B | 5 | 2.28 | 0.01 | Cytoplasm |  | Major role in the synthesis of nucleoside triphosphates other than ATP. The ATP gamma phosphate is transferred to the NDP beta phosphate via a ping-pong mechanism, using a phosphorylated active-site intermediate (By similarity). Negatively regulates Rho activity by interacting with AKAP13/LBC. |
| A8MXV4 | Nucleoside diphosphate-linked moiety X motif 19 | 9 | 2.27 | 0.00 | Peroxisome |  | Coenzyme A diphosphatase that mediates the hydrolysis of a wide range of CoA esters, including choloyl-CoA and branched-chain fatty-acyl-CoA esters. At low substrate concentrations medium and long-chain fatty-acyl-CoA esters are the primary substrates. |
| O75665 | Oral-facial-digital syndrome 1 protein | 5 | 2.49 | 0.00 | Cytoplasm, cytoskeleton, microtubule organizing center, centrosome, centriole | Orofaciodigital syndrome 1 (OFD1) | Component of the centrioles controlling mother and daughter centrioles length. Recruits to the centriole IFT88 and centriole distal appendage-specific proteins including CEP164. Involved in the biogenesis of the cilium, a centriole-associated function. The cilium is a cell surface projection found in many vertebrate cells required to transduce signals important for development and tissue homeostasis. Plays an important role in development by regulating Wnt signaling and the specification of the left-right axis. Only OFD1 localized at the centriolar satellites is removed by autophagy, which is an important step in the ciliogenesis regulation. |
| Q8TAD7 | Overexpressed in colon carcinoma 1 protein | 41 | 2.11 | 0.00 |  |  |  |
| Q8WX93 | Palladin | 10 | 2.21 | 0.01 | Cytoplasm, cytoskeleton | Pancreatic cancer 1 (PNCA1) | Cytoskeletal protein required for organization of normal actin cytoskeleton. Roles in establishing cell morphology, motility, cell adhesion and cell-extracellular matrix interactions in a variety of cell types. May function as a scaffolding molecule with the potential to influence both actin polymerization and the assembly of existing actin filaments into higher-order arrays. Binds to proteins that bind to either monomeric or filamentous actin. Localizes at sites where active actin remodeling takes place, such as lamellipodia and membrane ruffles. Different isoforms may have functional differences. Involved in the control of morphological and cytoskeletal changes associated with dendritic cell maturation. Involved in targeting ACTN to specific subcellular foci. |
| Q96JY6 | PDZ and LIM domain protein 2 | 7 | 2.43 | 0.00 | Cytoplasm |  | Probable adapter protein located at the actin cytoskeleton that promotes cell attachment. Necessary for the migratory capacity of epithelial cells. Overexpression enhances cell adhesion to collagen and fibronectin and suppresses anchorage independent growth. May contribute to tumor cell migratory capacity. |
| Q9Y3C6 | Peptidyl-prolyl cis-trans isomerase-like 1 | 18 | 2.67 | 0.00 | Nucleus |  | Involved in pre-mRNA splicing as component of the spliceosome. |
| Q99541 | Perilipin-2 | 11 | 2.74 | 0.00 | Membrane |  | May be involved in development and maintenance of adipose tissue. |
| Q13610 | Periodic tryptophan protein 1 homolog | 37 | 2.07 | 0.00 | Nucleus |  | Chromatin-associated factor that regulates transcription. |
| Q15063 | Periostin | 19 | 2.25 | 0.00 | Golgi apparatus |  | Induces cell attachment and spreading and plays a role in cell adhesion. |
| O15254 | Peroxisomal acyl-coenzyme A oxidase 3 | 13 | 5.53 | 0.00 | Peroxisome |  | Oxidizes the CoA-esters of 2-methyl-branched fatty acids. |
| P56589 | Peroxisomal biogenesis factor 3 | 8 | 57.75 | 0.03 | Peroxisome membrane | Peroxisome biogenesis disorder complementation group 12 (PBD-CG12) | Involved in peroxisome biosynthesis and integrity. Assembles membrane vesicles before the matrix proteins are translocated. As a docking factor for PEX19, is necessary for the import of peroxisomal membrane proteins in the peroxisomes. |
| Q8IWS0 | PHD finger protein 6 | 2 | 3.13 | 0.02 | Nucleus. Nucleus, nucleolus. Chromosome, centromere, kinetochore | Boerjeson-Forssman-Lehmann syndrome (BFLS) | Transcriptional regulator that associates with ribosomal RNA promoters and suppresses ribosomal RNA (rRNA) transcription. |
| Q9Y2H2 | Phosphatidylinositide phosphatase SAC2 | 28 | 3.69 | 0.00 | Membrane, clathrin-coated pit |  | Inositol 4-phosphatase which mainly acts on phosphatidylinositol 4-phosphate. May be functionally linked to OCRL, which converts phosphatidylinositol 4,5-bisphosphate to phosphatidylinositol, for a sequential dephosphorylation of phosphatidylinositol 4,5-bisphosphate at the 5 and 4 position of inositol, thus playing an important role in the endocytic recycling. |
| Q10472 | Polypeptide N-acetylgalactosaminyltransferase 1 | 10 | 2.41 | 0.00 |  |  | Catalyzes the initial reaction in O-linked oligosaccharide biosynthesis, the transfer of an N-acetyl-D-galactosamine residue to a serine or threonine residue on the protein receptor. Has a broad spectrum of substrates for peptides such as EA2, Muc5AC, Muc1a, Muc1b and Muc7. |
| Q9UKA9 | Polypyrimidine tract-binding protein 2 | 12 | 2.10 | 0.00 | Nucleus |  | RNA-binding protein which binds to intronic polypyrimidine tracts and mediates negative regulation of exons splicing. May antagonize in a tissue-specific manner the ability of NOVA1 to activate exon selection. In addition to its function in pre-mRNA splicing, plays also a role in the regulation of translation. Isoform 5 has a reduced affinity for RNA. |
| P0CG38 | POTE ankyrin domain family member I | 8 | 4.60 | 0.03 |  |  |  |
| P20742 | Pregnancy zone protein | 2 | 22.23 | 0.00 | Secreted. |  | Is able to inhibit all four classes of proteinases by a unique 'trapping' mechanism. This protein has a peptide stretch, called the 'bait region' which contains specific cleavage sites for different proteinases. When a proteinase cleaves the bait region, a conformational change is induced in the protein which traps the proteinase. The entrapped enzyme remains active against low molecular weight substrates (activity against high molecular weight substrates is greatly reduced). Following cleavage in the bait region a thioester bond is hydrolyzed and mediates the covalent binding of the protein to the proteinase. |
| Q8NBM8 | Prenylcysteine oxidase-like | 2 | 8.20 | 0.01 | Secreted. |  | Probable oxidoreductase. |
| Q96IZ0 | PRKC apoptosis WT1 regulator protein | 2 | 3.17 | 0.00 | Cytoplasm. Nucleus. |  | Pro-apoptotic protein capable of selectively inducing apoptosis in cancer cells, sensitizing the cells to diverse apoptotic stimuli and causing regression of tumors in animal models. Induces apoptosis in certain cancer cells by activation of the Fas prodeath pathway and coparallel inhibition of NF-kappa-B transcriptional activity. Inhibits the transcriptional activation and augments the transcriptional repression mediated by WT1. Down-regulates the anti-apoptotic protein BCL2 via its interaction with WT1. Seems also to be a transcriptional repressor by itself. May be directly involved in regulating the amyloid precursor protein (APP) cleavage activity of BACE1. |
| Q8NDH3 | Probable aminopeptidase NPEPL1 | 14 | 2.01 | 0.03 |  |  | Probably catalyzes the removal of unsubstituted N-terminal amino acids from various peptides. |
| Q9Y6V7 | Probable ATP-dependent RNA helicase DDX49 | 12 | 3.25 | 0.00 |  |  |  |
| O00469 | Procollagen-lysine,2-oxoglutarate 5-dioxygenase 2 | 6 | 12.42 | 0.00 | Rough endoplasmic reticulum membrane; Peripheral membrane protein; Lumenal side. | Bruck syndrome 2 (BRKS2) | Forms hydroxylysine residues in -Xaa-Lys-Gly- sequences in collagens. These hydroxylysines serve as sites of attachment for carbohydrate units and are essential for the stability of the intermolecular collagen cross-links. |
| P07602 | Prosaposin | 25 | 2.65 | 0.00 | Lysosome | Combined saposin deficiency (CSAPD) | Saposin-A and saposin-C stimulate the hydrolysis of glucosylceramide by beta-glucosylceramidase (EC 3.2.1.45) and galactosylceramide by beta-galactosylceramidase (EC 3.2.1.46). Saposin-C apparently acts by combining with the enzyme and acidic lipid to form an activated complex, rather than by solubilizing the substrate. |
| Q15185 | Prostaglandin E synthase 3 | 32 | 4.81 | 0.02 | Cytoplasm |  | Cytosolic prostaglandin synthase that catalyzes the oxidoreduction of prostaglandin endoperoxide H2 (PGH2) to prostaglandin E2 (PGE2). |
| Q8WUW1 | Protein BRICK1 | 11 | 3.16 | 0.00 | Cytoplasm, cytoskeleton |  | Involved in regulation of actin and microtubule organization. Part of a WAVE complex that activates the Arp2/3 complex. As component of the WAVE1 complex, required for BDNF-NTRK2 endocytic trafficking and signaling from early endosomes. |
| O60888 | Protein CutA | 8 | 3.49 | 0.00 |  |  | May form part of a complex of membrane proteins attached to acetylcholinesterase (AChE). |
| Q9C005 | Protein dpy-30 homolog | 5 | 2.27 | 0.00 | Nucleus |  | As part of the MLL1/MLL complex, involved in the methylation of histone H3 at 'Lys-4', particularly trimethylation. Histone H3 'Lys-4' methylation represents a specific tag for epigenetic transcriptional activation. May play some role in histone H3 acetylation. In a teratocarcinoma cell, plays a crucial role in retinoic acid-induced differentiation along the neural lineage, regulating gene induction and H3 'Lys-4' methylation at key developmental loci. May also play an indirect or direct role in endosomal transport. |
| Q96KR6 | Protein FAM210B, mitochondrial | 4 | 7.32 | 0.00 | Mitochondrion |  | Plays a role in erythroid differentiation. |
| Q8N9T8 | Protein KRI1 homolog | 3 | 2.19 | 0.02 |  |  |  |
| Q460N5 | Protein mono-ADP-ribosyltransferase PARP14 | 11 | 2.94 | 0.00 | Nucleus |  | ADP-ribosyltransferase that mediates mono-ADP-ribosylation of glutamate residues on target proteins. |
| P39210 | Protein Mpv17 | 2 | 2.20 | 0.02 | Mitochondrion inner membrane | Mitochondrial DNA depletion syndrome 6 (MTDPS6) | Non-selective channel that modulates the membrane potential under normal conditions and oxidative stress, and is involved in mitochondrial homeostasis. |
| P29590 | Protein PML | 36 | 2.05 | 0.00 | Nucleus. Nucleus, nucleoplasm. Cytoplasm | Note=A chromosomal aberration involving PML may be a cause of acute promyelocytic leukemia (APL). Translocation t(15;17)(q21;q21) with RARA. The PML breakpoints (type A and type B) lie on either side of an alternatively spliced exon. {ECO | Functions via its association with PML-nuclear bodies (PML-NBs) in a wide range of important cellular processes, including tumor suppression, transcriptional regulation, apoptosis, senescence, DNA damage response, and viral defense mechanisms. Acts as the scaffold of PML-NBs allowing other proteins to shuttle in and out, a process which is regulated by SUMO-mediated modifications and interactions. Isoform PML-4 has a multifaceted role in the regulation of apoptosis and growth suppression. |
| P31949 | Protein S100-A11 | 8 | 2.93 | 0.00 | Cytoplasm |  | Facilitates the differentiation and the cornification of keratinocytes. |
| Q96FQ6 | Protein S100-A16 | 9 | 2.20 | 0.02 | Nucleus, nucleolus |  | Calcium-binding protein. Binds one calcium ion per monomer. |
| P21980 | Protein-glutamine gamma-glutamyltransferase 2 | 29 | 3.48 | 0.00 |  |  | Catalyzes the cross-linking of proteins, such as WDR54, and the conjugation of polyamines to proteins. |
| P06454 | Prothymosin alpha [Cleaved into: Prothymosin alpha, N-terminally processed; Thymosin alpha-1] | 3 | 6.51 | 0.01 | Nucleus. |  | Prothymosin alpha may mediate immune function by conferring resistance to certain opportunistic infections. |
| Q6GMV3 | Putative peptidyl-tRNA hydrolase PTRHD1 | 6 | 2.34 | 0.02 |  |  |  |
| Q6IAA8 | Ragulator complex protein LAMTOR1 | 12 | 2.11 | 0.00 | Late endosome membrane; Lipid-anchor; Cytoplasmic side. Lysosome membrane; Lipid-anchor; Cytoplasmic side. Cell membrane. |  | As part of the Ragulator complex it is involved in amino acid sensing and activation of mTORC1, a signaling complex promoting cell growth in response to growth factors, energy levels, and amino acids. Activated by amino acids through a mechanism involving the lysosomal V-ATPase, the Ragulator functions as a guanine nucleotide exchange factor activating the small GTPases Rag. Activated Ragulator and Rag GTPases function as a scaffold recruiting mTORC1 to lysosomes where it is in turn activated. LAMTOR1 is directly responsible for anchoring the Ragulator complex to membranes. Also required for late endosomes/lysosomes biogenesis it may regulate both the recycling of receptors through endosomes and the MAPK signaling pathway through recruitment of some of its components to late endosomes. May be involved in cholesterol homeostasis regulating LDL uptake and cholesterol release from late endosomes/lysosomes. May also play a role in RHOA activation. |
| O43504 | Ragulator complex protein LAMTOR5 | 7 | 2.44 | 0.03 | Cytoplasm. Lysosome. |  | As part of the Ragulator complex it is involved in amino acid sensing and activation of mTORC1, a signaling complex promoting cell growth in response to growth factors, energy levels, and amino acids. Activated by amino acids through a mechanism involving the lysosomal V-ATPase, the Ragulator functions as a guanine nucleotide exchange factor activating the small GTPases Rag. Activated Ragulator and Rag GTPases function as a scaffold recruiting mTORC1 to lysosomes where it is in turn activated. When complexed to BIRC5, interferes with apoptosome assembly, preventing recruitment of pro-caspase-9 to oligomerized APAF1, thereby selectively suppressing apoptosis initiated via the mitochondrial/cytochrome c pathway. Down-regulates hepatitis B virus (HBV) replication. |
| Q86VI3 | Ras GTPase-activating-like protein IQGAP3 | 29 | 2.53 | 0.02 |  |  |  |
| Q9Y3L5 | Ras-related protein Rap-2c | 6 | 2.19 | 0.00 | Cytoplasm |  | Small GTP-binding protein which cycles between a GDP-bound inactive and a GTP-bound active form. May play a role in cytoskeletal rearrangements and regulate cell spreading through activation of the effector TNIK. May play a role in SRE-mediated gene transcription. |
| P17081 | Rho-related GTP-binding protein RhoQ | 3 | 4.60 | 0.00 | Cytoplasm |  | Plasma membrane-associated small GTPase which cycles between an active GTP-bound and an inactive GDP-bound state. In active state binds to a variety of effector proteins to regulate cellular responses. Involved in epithelial cell polarization processes. May play a role in CFTR trafficking to the plasma membrane. Causes the formation of thin, actin-rich surface projections called filopodia. |
| P13489 | Ribonuclease inhibitor | 27 | 2.20 | 0.01 | Cytoplasm. |  | Ribonuclease inhibitor which inhibits RNASE1, RNASE2 and ANG. May play a role in redox homeostasis. |
| O95478 | Ribosome biogenesis protein NSA2 homolog | 10 | 2.36 | 0.01 | Nucleus, nucleolus |  | Involved in the biogenesis of the 60S ribosomal subunit. May play a part in the quality control of pre-60S particles. |
| Q5EBL4 | RILP-like protein 1 | 15 | 2.48 | 0.00 | Cytoplasm, cytosol |  | Plays a role in the regulation of cell shape and polarity (By similarity). Plays a role in cellular protein transport, including protein transport away from primary cilia (By similarity). Neuroprotective protein, which acts by sequestring GAPDH in the cytosol and prevent the apoptotic function of GAPDH in the nucleus (By similarity). Competes with SIAH1 for binding GAPDH (By similarity). Does not regulate lysosomal morphology and distribution. |
| Q15434 | RNA-binding motif, single-stranded-interacting protein 2 | 4 | 2.07 | 0.00 | Nucleus |  |  |
| P42696 | RNA-binding protein 34 | 15 | 2.85 | 0.00 | Nucleus, nucleolus |  |  |
| O60613 | Selenoprotein F | 5 | 2.02 | 0.00 | Endoplasmic reticulum lumen |  | May be involved in redox reactions associated with the formation of disulfide bonds (By similarity). May contribute to the quality control of protein folding in the endoplasmic reticulum. |
| Q8WWX9 | Selenoprotein M | 7 | 2.87 | 0.01 | Cytoplasm, perinuclear region |  | May function as a thiol-disulfide oxidoreductase that participates in disulfide bond formation. |
| Q9NPR2 | Semaphorin-4B | 2 | 4.08 | 0.02 | Membrane; Single-pass type I membrane protein. |  | Inhibits axonal extension by providing local signals to specify territories inaccessible for growing axons. |
| P30154 | Serine/threonine-protein phosphatase 2A 65 kDa regulatory subunit A beta isoform | 6 | 4.76 | 0.02 |  |  | The PR65 subunit of protein phosphatase 2A serves as a scaffolding molecule to coordinate the assembly of the catalytic subunit and a variable regulatory B subunit. |
| Q9NY27 | Serine/threonine-protein phosphatase 4 regulatory subunit 2 | 20 | 3.39 | 0.00 | Cytoplasm, cytoskeleton, microtubule organizing center, centrosome. Nucleus. |  | Regulatory subunit of serine/threonine-protein phosphatase 4 (PP4). May regulate the activity of PPP4C at centrosomal microtubule organizing centers. Its interaction with the SMN complex leads to enhance the temporal localization of snRNPs, suggesting a role of PPP4C in maturation of spliceosomal snRNPs. The PPP4C-PPP4R2-PPP4R3A PP4 complex specifically dephosphorylates H2AX phosphorylated on 'Ser-140' (gamma-H2AX) generated during DNA replication and required for DNA double strand break repair. Mediates RPA2 dephosphorylation by recruiting PPP4C to RPA2 in a DNA damage-dependent manner. RPA2 dephosphorylation is required for the efficient RPA2-mediated recruitment of RAD51 to chromatin following double strand breaks, an essential step for DNA repair. |
| Q9H788 | SH2 domain-containing protein 4A | 21 | 2.48 | 0.00 | Cytoplasm |  | Inhibits estrogen-induced cell proliferation by competing with PLCG for binding to ESR1, blocking the effect of estrogen on PLCG and repressing estrogen-induced proliferation. May play a role in T-cell development and function. |
| P42224 | Signal transducer and activator of transcription 1-alpha/beta | 55 | 2.04 | 0.00 | Cytoplasm | Immunodeficiency 31B (IMD31B) | Signal transducer and transcription activator that mediates cellular responses to interferons (IFNs), cytokine KITLG/SCF and other cytokines and other growth factors. Following type I IFN (IFN-alpha and IFN-beta) binding to cell surface receptors, signaling via protein kinases leads to activation of Jak kinases (TYK2 and JAK1) and to tyrosine phosphorylation of STAT1 and STAT2. The phosphorylated STATs dimerize and associate with ISGF3G/IRF-9 to form a complex termed ISGF3 transcription factor, that enters the nucleus. |
| P51692 | Signal transducer and activator of transcription 5B | 4 | 6.28 | 0.00 | Cytoplasm | Growth hormone insensitivity with immunodeficiency (GHII) | Carries out a dual function |
| Q96AG3 | Solute carrier family 25 member 46 | 5 | 5.34 | 0.00 | Mitochondrion outer membrane | Neuropathy, hereditary motor and sensory, 6B (HMSN6B) | May play a role in mitochondrial dynamics by controlling mitochondrial membrane fission. |
| Q9NXE4 | Sphingomyelin phosphodiesterase 4 | 19 | 3.39 | 0.04 | Endoplasmic reticulum membrane |  | Catalyzes the hydrolysis of membrane sphingomyelin to form phosphorylcholine and ceramide. |
| Q9UBI4 | Stomatin-like protein 1 | 4 | 5.81 | 0.00 | Membrane |  | May play a role in cholesterol transfer to late endosomes. |
| Q9UMS6 | Synaptopodin-2 | 28 | 3.27 | 0.01 | Nucleus |  | Has an actin-binding and actin-bundling activity. Can induce the formation of F-actin networks in an isoform-specific manner. |
| P34741 | Syndecan-2 | 7 | 2.17 | 0.00 | Membrane; Single-pass type I membrane protein. |  | Cell surface proteoglycan that bears heparan sulfate. Regulates dendritic arbor morphogenesis. |
| O14981 | TATA-binding protein-associated factor 172 | 26 | 2.92 | 0.00 | Nucleus. |  | Regulates transcription in association with TATA binding protein (TBP). Removes TBP from the TATA box in an ATP-dependent manner. |
| O60637 | Tetraspanin-3 | 4 | 2.55 | 0.00 | Membrane |  | Regulates the proliferation and migration of oligodendrocytes, a process essential for normal myelination and repair. |
| P35442 | Thrombospondin-2 | 24 | 2.48 | 0.00 |  | Intervertebral disc disease (IDD) | Adhesive glycoprotein that mediates cell-to-cell and cell-to-matrix interactions. Ligand for CD36 mediating antiangiogenic properties. |
| P13726 | Tissue factor | 7 | 2.79 | 0.00 |  |  | Initiates blood coagulation by forming a complex with circulating factor VII or VIIa. The [TF |
| Q12788 | Transducin beta-like protein 3 | 19 | 18.44 | 0.04 | Nucleus, nucleolus |  |  |
| Q01995 | Transgelin | 36 | 2.79 | 0.00 | Cytoplasm |  | Actin cross-linking/gelling protein (By similarity). Involved in calcium interactions and contractile properties of the cell that may contribute to replicative senescence. |
| Q9Y5S1 | Transient receptor potential cation channel subfamily V member 2 | 14 | 2.19 | 0.00 | Cell membrane |  | Calcium-permeable, non-selective cation channel with an outward rectification. Seems to be regulated, at least in part, by IGF-I, PDGF and neuropeptide head activator. May transduce physical stimuli in mast cells. Activated by temperatures higher than 52 degrees Celsius; is not activated by vanilloids and acidic pH. |
| Q9BVT8 | Transmembrane and ubiquitin-like domain-containing protein 1 | 2 | 2.64 | 0.02 | Membrane |  | Involved in sterol-regulated ubiquitination and degradation of HMG-CoA reductase HMGCR. |
| Q96A57 | Transmembrane protein 230 | 3 | 3.68 | 0.00 | Membrane | Parkinson disease (PARK) | Involved in trafficking and recycling of synaptic vesicles. |
| P61165 | Transmembrane protein 258 | 5 | 2.19 | 0.00 | Membrane |  | Subunit of the oligosaccharyl transferase (OST) complex that catalyzes the initial transfer of a defined glycan (Glc(3)Man(9)GlcNAc(2) in eukaryotes) from the lipid carrier dolichol-pyrophosphate to an asparagine residue within an Asn-X-Ser/Thr consensus motif in nascent polypeptide chains, the first step in protein N-glycosylation. N-glycosylation occurs cotranslationally and the complex associates with the Sec61 complex at the channel-forming translocon complex that mediates protein translocation across the endoplasmic reticulum (ER). All subunits are required for a maximal enzyme activity. |
| Q6PI78 | Transmembrane protein 65 | 5 | 2.01 | 0.00 | Cell membrane | Note=Defects in TMEM65 may cause a mitochondrial disorder characterized by a complex encephalomyopathic phenotype. Clinical features includ microcephaly, dysmorphic features, psychomotor regression, hypotonia, growth retardation, lactic acidosis, intractable seizures, dyskenetics movements, without cardiomyopathy (PubMed | May play an important role in cardiac development and function. May regulate cardiac conduction and the function of the gap junction protein GJA1. May contribute to the stability and proper localization of GJA1 to cardiac intercalated disk thereby regulating gap junction communication (By similarity). May also play a role in the regulation of mitochondrial respiration and mitochondrial DNA copy number maintenance. |
| Q9UBP6 | tRNA | 7 | 2.10 | 0.03 | Nucleus |  | Methyltransferase that mediates the formation of N(7)-methylguanine in a subset of RNA species, such as tRNAs, mRNAs and microRNAs (miRNAs). |
| Q13641 | Trophoblast glycoprotein | 6 | 2.16 | 0.00 | Cell membrane |  | May function as an inhibitor of Wnt/beta-catenin signaling by indirectly interacting with LRP6 and blocking Wnt3a-dependent LRP6 internalization. |
| P07951 | Tropomyosin beta chain | 18 | 2.40 | 0.00 | Cytoplasm, cytoskeleton | Nemaline myopathy 4 (NEM4) | Binds to actin filaments in muscle and non-muscle cells. Plays a central role, in association with the troponin complex, in the calcium dependent regulation of vertebrate striated muscle contraction. Smooth muscle contraction is regulated by interaction with caldesmon. In non-muscle cells is implicated in stabilizing cytoskeleton actin filaments. The non-muscle isoform may have a role in agonist-mediated receptor internalization. |
| Q9BQE3 | Tubulin alpha-1C chain | 5 | 2.79 | 0.00 | Cytoplasm, cytoskeleton. |  | Tubulin is the major constituent of microtubules. It binds two moles of GTP, one at an exchangeable site on the beta chain and one at a non-exchangeable site on the alpha chain. |
| Q9BUF5 | Tubulin beta-6 chain | 19 | 2.24 | 0.00 | Cytoplasm, cytoskeleton | Facial palsy, congenital, with ptosis and velopharyngeal dysfunction (FPVEPD) | Tubulin is the major constituent of microtubules. It binds two moles of GTP, one at an exchangeable site on the beta chain and one at a non-exchangeable site on the alpha chain. |
| Q03169 | Tumor necrosis factor alpha-induced protein 2 | 13 | 2.46 | 0.00 |  |  | May play a role as a mediator of inflammation and angiogenesis. |
| P07947 | Tyrosine-protein kinase Yes | 8 | 2.22 | 0.05 | Cell membrane. Cytoplasm, cytoskeleton, microtubule organizing center, centrosome. Cytoplasm, cytosol. |  | Non-receptor protein tyrosine kinase that is involved in the regulation of cell growth and survival, apoptosis, cell-cell adhesion, cytoskeleton remodeling, and differentiation. Stimulation by receptor tyrosine kinases (RTKs) including EGRF, PDGFR, CSF1R and FGFR leads to recruitment of YES1 to the phosphorylated receptor, and activation and phosphorylation of downstream substrates. Upon EGFR activation, promotes the phosphorylation of PARD3 to favor epithelial tight junction assembly. Participates in the phosphorylation of specific junctional components such as CTNND1 by stimulating the FYN and FER tyrosine kinases at cell-cell contacts. Upon T-cell stimulation by CXCL12, phosphorylates collapsin response mediator protein 2/DPYSL2 and induces T-cell migration. Participates in CD95L/FASLG signaling pathway and mediates AKT-mediated cell migration. Plays a role in cell cycle progression by phosphorylating the cyclin-dependent kinase 4/CDK4 thus regulating the G1 phase. Also involved in G2/M progression and cytokinesis. |
| O00566 | U3 small nucleolar ribonucleoprotein protein MPP10 | 15 | 3.75 | 0.02 | Nucleus, nucleolus |  | Component of the 60-80S U3 small nucleolar ribonucleoprotein (U3 snoRNP). Required for the early cleavages during pre-18S ribosomal RNA processing. |
| Q8WWY3 | U4/U6 small nuclear ribonucleoprotein Prp31 | 21 | 2.08 | 0.01 | Nucleus | Retinitis pigmentosa 11 (RP11) | Involved in pre-mRNA splicing as component of the spliceosome. |
| Q9Y333 | U6 snRNA-associated Sm-like protein LSm2 | 5 | 2.34 | 0.04 | Nucleus |  | Plays role in pre-mRNA splicing as component of the U4/U6-U5 tri-snRNP complex that is involved in spliceosome assembly, and as component of the precatalytic spliceosome (spliceosome B complex). |
| O95777 | U6 snRNA-associated Sm-like protein LSm8 | 4 | 4.08 | 0.00 | Nucleus |  | Plays role in pre-mRNA splicing as component of the U4/U6-U5 tri-snRNP complex that is involved in spliceosome assembly, and as component of the precatalytic spliceosome (spliceosome B complex). |
| Q6BDS2 | UHRF1-binding protein 1 | 5 | 2.41 | 0.00 |  |  | May act as a negative regulator of cell growth. |
| Q9H425 | Uncharacterized protein C1orf198 | 19 | 3.48 | 0.00 | Cytoplasm |  |  |
| O94854 | Uncharacterized protein KIAA0754 | 11 | 2.68 | 0.01 |  |  |  |
| Q9UFG5 | UPF0449 protein C19orf25 | 4 | 2.14 | 0.01 |  |  |  |
| Q12981 | Vesicle transport protein SEC20 | 9 | 3.27 | 0.01 | Endoplasmic reticulum membrane |  | As part of a SNARE complex may be involved in endoplasmic reticulum membranes fusion and be required for the maintenance of endoplasmic reticulum organization. |
| Q9UNX4 | WD repeat-containing protein 3 | 16 | 3.08 | 0.00 | Nucleus, nucleolus |  |  |
| Q9Y3S2 | Zinc finger protein 330 | 7 | 8.82 | 0.01 | Nucleus |  |  |
| O95218 | Zinc finger Ran-binding domain-containing protein 2 | 19 | 2.00 | 0.02 | Nucleus |  | Splice factor required for alternative splicing of TRA2B/SFRS10 transcripts. May interfere with constitutive 5'-splice site selection. |
| Q9Y6M5 | Zinc transporter 1 | 13 | 2.71 | 0.00 | Cell membrane |  | May be involved in zinc transport out of the cell. |
| P49327 | Fatty acid synthase | 30 | 0.34 | 0.00 | Cytoplasm |  | Fatty acid synthetase catalyzes the formation of long-chain fatty acids from acetyl-CoA, malonyl-CoA and NADPH. This multifunctional protein has 7 catalytic activities as an acyl carrier protein. |
| P15924 | Desmoplakin | 4 | 0.33 | 0.03 | Cell junction, desmosome | Keratoderma, palmoplantar, striate 2 (SPPK2) | Major high molecular weight protein of desmosomes. Involved in the organization of the desmosomal cadherin-plakoglobin complexes into discrete plasma membrane domains and in the anchoring of intermediate filaments to the desmosomes. |
| P02768 | Serum albumin | 5 | 0.48 | 0.03 | Secreted. | Hyperthyroxinemia, familial dysalbuminemic (FDAH) | Serum albumin, the main protein of plasma, has a good binding capacity for water, Ca(2+), Na(+), K(+), fatty acids, hormones, bilirubin and drugs (Probable). Its main function is the regulation of the colloidal osmotic pressure of blood (Probable). Major zinc transporter in plasma, typically binds about 80% of all plasma zinc |
| P04264 | Keratin, type II cytoskeletal 1 | 3 | 0.48 | 0.00 | Cell membrane | Epidermolytic hyperkeratosis (EHK) | May regulate the activity of kinases such as PKC and SRC via binding to integrin beta-1 (ITB1) and the receptor of activated protein C kinase 1 (RACK1). In complex with C1QBP is a high affinity receptor for kininogen-1/HMWK. |
| P35527 | Keratin, type I cytoskeletal 9 | 10 | 0.44 | 0.01 |  | Keratoderma, palmoplantar, epidermolytic (EPPK) | May serve an important special function either in the mature palmar and plantar skin tissue or in the morphogenetic program of the formation of these tissues. Plays a role in keratin filament assembly. |
| Q01813 | ATP-dependent 6-phosphofructokinase, platelet type | 7 | 0.20 | 0.00 | Cytoplasm |  | Catalyzes the phosphorylation of D-fructose 6-phosphate to fructose 1,6-bisphosphate by ATP, the first committing step of glycolysis. |
| Q15154 | Pericentriolar material 1 protein | 2 | 0.14 | 0.00 | Cytoplasm, cytoskeleton. Cytoplasm, cytoskeleton, microtubule organizing center, centrosome |  | Required for centrosome assembly and function |
| Q8IUD2 | ELKS/Rab6-interacting/CAST family member 1 | 16 | 0.49 | 0.00 | Cytoplasm, cytoskeleton, microtubule organizing center, centrosome |  | Regulatory subunit of the IKK complex. Probably recruits IkappaBalpha/NFKBIA to the complex. May be involved in the organization of the cytomatrix at the nerve terminals active zone (CAZ) which regulates neurotransmitter release. May be involved in vesicle trafficking at the CAZ. May be involved in Rab-6 regulated endosomes to Golgi transport. |
| Q9UIG0 | Tyrosine-protein kinase BAZ1B | 8 | 0.29 | 0.02 | Nucleus |  | Atypical tyrosine-protein kinase that plays a central role in chromatin remodeling and acts as a transcription regulator. Involved in DNA damage response by phosphorylating 'Tyr-142' of histone H2AX (H2AXY142ph). H2AXY142ph plays a central role in DNA repair and acts as a mark that distinguishes between apoptotic and repair responses to genotoxic stress. Essential component of the WICH complex, a chromatin remodeling complex that mobilizes nucleosomes and reconfigures irregular chromatin to a regular nucleosomal array structure. The WICH complex regulates the transcription of various genes, has a role in RNA polymerase I and RNA polymerase III transcription, mediates the histone H2AX phosphorylation at 'Tyr-142', and is involved in the maintenance of chromatin structures during DNA replication processes. In the complex, it mediates the recruitment of the WICH complex to replication foci during DNA replication. |
| Q6DD88 | Atlastin-3 | 4 | 0.14 | 0.00 | Endoplasmic reticulum membrane | Neuropathy, hereditary sensory, 1F (HSN1F) | GTPase tethering membranes through formation of trans-homooligomers and mediating homotypic fusion of endoplasmic reticulum membranes. Functions in endoplasmic reticulum tubular network biogenesis |
| O95347 | Structural maintenance of chromosomes protein 2 | 25 | 0.39 | 0.00 | Nucleus |  | Central component of the condensin complex, a complex required for conversion of interphase chromatin into mitotic-like condense chromosomes. The condensin complex probably introduces positive supercoils into relaxed DNA in the presence of type I topoisomerases and converts nicked DNA into positive knotted forms in the presence of type II topoisomerases. |
| O14497 | AT-rich interactive domain-containing protein 1A | 5 | 0.45 | 0.01 | Nucleus | Coffin-Siris syndrome 2 (CSS2) | Involved in transcriptional activation and repression of select genes by chromatin remodeling (alteration of DNA-nucleosome topology). Component of SWI/SNF chromatin remodeling complexes that carry out key enzymatic activities, changing chromatin structure by altering DNA-histone contacts within a nucleosome in an ATP-dependent manner. Binds DNA non-specifically. Belongs to the neural progenitors-specific chromatin remodeling complex (npBAF complex) and the neuron-specific chromatin remodeling complex (nBAF complex). During neural development a switch from a stem/progenitor to a postmitotic chromatin remodeling mechanism occurs as neurons exit the cell cycle and become committed to their adult state. The transition from proliferating neural stem/progenitor cells to postmitotic neurons requires a switch in subunit composition of the npBAF and nBAF complexes. As neural progenitors exit mitosis and differentiate into neurons, npBAF complexes which contain ACTL6A/BAF53A and PHF10/BAF45A, are exchanged for homologous alternative ACTL6B/BAF53B and DPF1/BAF45B or DPF3/BAF45C subunits in neuron-specific complexes (nBAF). The npBAF complex is essential for the self-renewal/proliferative capacity of the multipotent neural stem cells. The nBAF complex along with CREST plays a role regulating the activity of genes essential for dendrite growth. |
| O00291 | Huntingtin-interacting protein 1 | 6 | 0.27 | 0.00 | Cytoplasm. Nucleus. Endomembrane system. Cytoplasmic vesicle, clathrin-coated vesicle membrane. |  | Plays a role in clathrin-mediated endocytosis and trafficking. |
| P13647 | Keratin, type II cytoskeletal 5 | 5 | 0.33 | 0.03 |  | Epidermolysis bullosa simplex, autosomal recessive 1 (EBSB1) |  |
| Q05193 | Dynamin-1 | 10 | 0.45 | 0.01 | Cytoplasm | Epileptic encephalopathy, early infantile, 31 (EIEE31) | Microtubule-associated force-producing protein involved in producing microtubule bundles and able to bind and hydrolyze GTP. Most probably involved in vesicular trafficking processes. Involved in receptor-mediated endocytosis. |
| Q9C0C9 | E3-independent) E2 ubiquitin-conjugating enzyme | 9 | 0.44 | 0.00 | Cytoplasm |  | E2/E3 hybrid ubiquitin-protein ligase that displays both E2 and E3 ligase activities and mediates monoubiquitination of target proteins. |
| Q4V328 | GRIP1-associated protein 1 | 8 | 0.46 | 0.04 | Early endosome membrane |  | Regulates the endosomal recycling back to the neuronal plasma membrane, possibly by connecting early and late recycling endosomal domains and promoting segregation of recycling endosomes from early endosomal membranes. Involved in the localization of recycling endosomes to dendritic spines, thereby playing a role in the maintenance of dendritic spine morphology. Required for the activity-induced AMPA receptor recycling to dendrite membranes and for long-term potentiation and synaptic plasticity. |
| P60228 | Eukaryotic translation initiation factor 3 subunit E | 21 | 0.28 | 0.00 | Cytoplasm. Nucleus, PML body. |  | Component of the eukaryotic translation initiation factor 3 (eIF-3) complex, which is required for several steps in the initiation of protein synthesis (PubMed |
| Q76M96 | Coiled-coil domain-containing protein 80 | 13 | 0.41 | 0.00 | Secreted, extracellular space, extracellular matrix |  | Promotes cell adhesion and matrix assembly. |
| Q9Y5X1 | Sorting nexin-9 | 3 | 0.48 | 0.01 | Cytoplasmic vesicle membrane; Peripheral membrane protein; Cytoplasmic side. Cell membrane; Peripheral membrane protein; Cytoplasmic side. Cytoplasmic vesicle, clathrin-coated vesicle. Golgi apparatus, trans-Golgi network. Cell projection, ruffle. Cytoplasm. |  | Involved in endocytosis and intracellular vesicle trafficking, both during interphase and at the end of mitosis. Required for efficient progress through mitosis and cytokinesis. Required for normal formation of the cleavage furrow at the end of mitosis. Plays a role in endocytosis via clathrin-coated pits, but also clathrin-independent, actin-dependent fluid-phase endocytosis. Plays a role in macropinocytosis. Promotes internalization of TNFR. Promotes degradation of EGFR after EGF signaling. Stimulates the GTPase activity of DNM1. Promotes DNM1 oligomerization. Promotes activation of the Arp2/3 complex by WASL, and thereby plays a role in the reorganization of the F-actin cytoskeleton. Binds to membranes enriched in phosphatidylinositol 4,5-bisphosphate and promotes membrane tubulation. Has lower affinity for membranes enriched in phosphatidylinositol 3-phosphate. |
| P39060 | Collagen alpha-1 | 8 | 0.49 | 0.01 | Secreted, extracellular space, extracellular matrix | Knobloch syndrome 1 (KNO1) | Probably plays a major role in determining the retinal structure as well as in the closure of the neural tube. |
| P61160 | Actin-related protein 2 | 34 | 0.49 | 0.00 | Cytoplasm, cytoskeleton |  | ATP-binding component of the Arp2/3 complex, a multiprotein complex that mediates actin polymerization upon stimulation by nucleation-promoting factor (NPF). |
| P13645 | Keratin, type I cytoskeletal 10 | 42 | 0.45 | 0.02 | Secreted, extracellular space | Epidermolytic hyperkeratosis (EHK) | Plays a role in the establishment of the epidermal barrier on plantar skin. |
| P22033 | Methylmalonyl-CoA mutase, mitochondrial | 32 | 0.12 | 0.01 | Mitochondrion matrix. | Methylmalonic aciduria type mut (MMAM) | Involved in the degradation of several amino acids, odd-chain fatty acids and cholesterol via propionyl-CoA to the tricarboxylic acid cycle. MCM has different functions in other species. |
| P08238 | Heat shock protein HSP 90-beta | 13 | 0.44 | 0.00 | Cytoplasm |  | Molecular chaperone that promotes the maturation, structural maintenance and proper regulation of specific target proteins involved for instance in cell cycle control and signal transduction. Undergoes a functional cycle that is linked to its ATPase activity. This cycle probably induces conformational changes in the client proteins, thereby causing their activation. Interacts dynamically with various co-chaperones that modulate its substrate recognition, ATPase cycle and chaperone function. |
| O43719 | HIV Tat-specific factor 1 | 8 | 0.36 | 0.04 | Nucleus |  | Functions as a general transcription factor playing a role in the process of transcriptional elongation. May mediate the reciprocal stimulatory effect of splicing on transcriptional elongation. In case of infection by HIV-1, it is up-regulated by the HIV-1 proteins NEF and gp120, acts as a cofactor required for the Tat-enhanced transcription of the virus. |
| P35908 | Keratin, type II cytoskeletal 2 epidermal | 5 | 0.32 | 0.01 |  | Ichthyosis bullosa of Siemens (IBS) | Probably contributes to terminal cornification (PubMed |
| O95819 | Mitogen-activated protein kinase kinase kinase kinase 4 | 2 | 0.44 | 0.00 | Cytoplasm |  | Serine/threonine kinase that may play a role in the response to environmental stress and cytokines such as TNF-alpha. Appears to act upstream of the JUN N-terminal pathway. Phosphorylates SMAD1 on Thr-322. |
| P47895 | Aldehyde dehydrogenase family 1 member A3 | 10 | 0.39 | 0.03 | Cytoplasm | Microphthalmia, isolated, 8 (MCOP8) | NAD-dependent aldehyde dehydrogenase that catalyzes the formation of retinoic acid |
| O14920 | Inhibitor of nuclear factor kappa-B kinase subunit beta | 4 | 0.41 | 0.01 | Cytoplasm | Immunodeficiency 15B (IMD15B) | Serine kinase that plays an essential role in the NF-kappa-B signaling pathway which is activated by multiple stimuli such as inflammatory cytokines, bacterial or viral products, DNA damages or other cellular stresses. |
| O95757 | Heat shock 70 kDa protein 4L | 8 | 0.49 | 0.02 | Cytoplasm |  | Possesses chaperone activity in vitro where it inhibits aggregation of citrate synthase. |
| P23229 | Integrin alpha-6 | 10 | 0.34 | 0.02 | Cell membrane | Epidermolysis bullosa letalis, with pyloric atresia (EB-PA) |  |
| O75153 | Clustered mitochondria protein homolog | 15 | 0.36 | 0.03 | Cytoplasm |  | mRNA-binding protein involved in proper cytoplasmic distribution of mitochondria. Specifically binds mRNAs of nuclear-encoded mitochondrial proteins in the cytoplasm and regulates transport or translation of these transcripts close to mitochondria, playing a role in mitochondrial biogenesis. |
| P08779 | Keratin, type I cytoskeletal 16 | 17 | 0.50 | 0.04 |  | Pachyonychia congenita 1 (PC1) | Epidermis-specific type I keratin that plays a key role in skin. Acts as a regulator of innate immunity in response to skin barrier breach. |
| Q7L0Y3 | tRNA methyltransferase 10 homolog C | 19 | 0.45 | 0.02 | Mitochondrion matrix, mitochondrion nucleoid | Combined oxidative phosphorylation deficiency 30 (COXPD30) | Mitochondrial tRNA N(1)-methyltransferase involved in mitochondrial tRNA maturation. |
| P05120 | Plasminogen activator inhibitor 2 | 26 | 0.41 | 0.05 | Cytoplasm. Secreted, extracellular space. |  | Inhibits urokinase-type plasminogen activator. The monocyte derived PAI-2 is distinct from the endothelial cell-derived PAI-1. |
| Q8WYL5 | Protein phosphatase Slingshot homolog 1 | 25 | 0.50 | 0.03 | Cytoplasm, cytoskeleton. Cell projection, lamellipodium. Cleavage furrow. Midbody. |  | Protein phosphatase which regulates actin filament dynamics. Dephosphorylates and activates the actin binding/depolymerizing factor cofilin, which subsequently binds to actin filaments and stimulates their disassembly. Inhibitory phosphorylation of cofilin is mediated by LIMK1, which may also be dephosphorylated and inactivated by this protein. |
| Q14738 | Serine/threonine-protein phosphatase 2A 56 kDa regulatory subunit delta isoform | 6 | 0.38 | 0.02 | Cytoplasm. Nucleus. | Mental retardation, autosomal dominant 35 (MRD35) | The B regulatory subunit might modulate substrate selectivity and catalytic activity, and also might direct the localization of the catalytic enzyme to a particular subcellular compartment. |
| Q92743 | Serine protease HTRA1 | 14 | 0.32 | 0.00 | Cell membrane | Macular degeneration, age-related, 7 (ARMD7) | Serine protease with a variety of targets, including extracellular matrix proteins such as fibronectin. HTRA1-generated fibronectin fragments further induce synovial cells to up-regulate MMP1 and MMP3 production. May also degrade proteoglycans, such as aggrecan, decorin and fibromodulin. Through cleavage of proteoglycans, may release soluble FGF-glycosaminoglycan complexes that promote the range and intensity of FGF signals in the extracellular space. Regulates the availability of insulin-like growth factors (IGFs) by cleaving IGF-binding proteins. Inhibits signaling mediated by TGF-beta family members. This activity requires the integrity of the catalytic site, although it is unclear whether TGF-beta proteins are themselves degraded. By acting on TGF-beta signaling, may regulate many physiological processes, including retinal angiogenesis and neuronal survival and maturation during development. Intracellularly, degrades TSC2, leading to the activation of TSC2 downstream targets. |
| Q96JM3 | Chromosome alignment-maintaining phosphoprotein 1 | 3 | 0.16 | 0.00 | Nucleus | Mental retardation, autosomal dominant 40 (MRD40) | Required for proper alignment of chromosomes at metaphase and their accurate segregation during mitosis. Involved in the maintenance of spindle microtubules attachment to the kinetochore during sister chromatid biorientation. May recruit CENPE and CENPF to the kinetochore. |
| Q01581 | Hydroxymethylglutaryl-CoA synthase, cytoplasmic | 2 | 0.31 | 0.00 | Cytoplasm. |  | This enzyme condenses acetyl-CoA with acetoacetyl-CoA to form HMG-CoA, which is the substrate for HMG-CoA reductase. |
| Q01831 | DNA repair protein complementing XP-C cells | 5 | 0.29 | 0.01 | Nucleus | Xeroderma pigmentosum complementation group C (XP-C) | Involved in global genome nucleotide excision repair (GG-NER) by acting as damage sensing and DNA-binding factor component of the XPC complex. |
| Q13131 | 5'-AMP-activated protein kinase catalytic subunit alpha-1 | 78 | 0.40 | 0.00 | Cytoplasm |  | Catalytic subunit of AMP-activated protein kinase (AMPK), an energy sensor protein kinase that plays a key role in regulating cellular energy metabolism. In response to reduction of intracellular ATP levels, AMPK activates energy-producing pathways and inhibits energy-consuming processes |
| P50452 | Serpin B8 | 16 | 0.34 | 0.01 | Cytoplasm. | Peeling skin syndrome 5 (PSS5) | Has an important role in epithelial desmosome-mediated cell-cell adhesion. |
| Q9BTE3 | Mini-chromosome maintenance complex-binding protein | 10 | 0.38 | 0.03 | Nucleus |  | Associated component of the MCM complex that acts as a regulator of DNA replication. Binds to the MCM complex during late S phase and promotes the disassembly of the MCM complex from chromatin, thereby acting as a key regulator of pre-replication complex (pre-RC) unloading from replicated DNA. Can dissociate the MCM complex without addition of ATP; probably acts by destabilizing interactions of each individual subunits of the MCM complex. Required for sister chromatid cohesion. |
| Q9ULG6 | Cell cycle progression protein 1 | 10 | 0.44 | 0.03 | Cytoplasmic granule membrane |  | Acts as an assembly platform for Rho protein signaling complexes. Limits guanine nucleotide exchange activity of MCF2L toward RHOA, which results in an inhibition of both its transcriptional activation ability and its transforming activity. Does not inhibit activity of MCF2L toward CDC42, or activity of MCF2 toward either RHOA or CDC42 (By similarity). May be involved in cell cycle regulation. |
| P02533 | Keratin, type I cytoskeletal 14 | 30 | 0.38 | 0.00 | Cytoplasm. Nucleus. | Epidermolysis bullosa simplex, Dowling-Meara type (DM-EBS) | The nonhelical tail domain is involved in promoting KRT5-KRT14 filaments to self-organize into large bundles and enhances the mechanical properties involved in resilience of keratin intermediate filaments in vitro. |
| P09871 | Complement C1s subcomponent | 35 | 0.35 | 0.00 |  | Complement component C1s deficiency (C1SD) | C1s B chain is a serine protease that combines with C1q and C1r to form C1, the first component of the classical pathway of the complement system. C1r activates C1s so that it can, in turn, activate C2 and C4. |
| P23919 | Thymidylate kinase | 3 | 0.34 | 0.01 |  |  | Catalyzes the conversion of dTMP to dTDP. |
| P45954 | Short/branched chain specific acyl-CoA dehydrogenase, mitochondrial | 8 | 0.43 | 0.00 | Mitochondrion matrix. | Short/branched-chain acyl-CoA dehydrogenase deficiency (SBCADD) | Has greatest activity toward short branched chain acyl-CoA derivative such as (s)-2-methylbutyryl-CoA, isobutyryl-CoA, and 2-methylhexanoyl-CoA as well as toward short straight chain acyl-CoAs such as butyryl-CoA and hexanoyl-CoA. Can use valproyl-CoA as substrate and may play a role in controlling the metabolic flux of valproic acid in the development of toxicity of this agent. |
| P51648 | Aldehyde dehydrogenase family 3 member A2 | 27 | 0.34 | 0.02 | Microsome membrane | Sjoegren-Larsson syndrome (SLS) | Catalyzes the oxidation of medium and long chain aliphatic aldehydes to fatty acids. Active on a variety of saturated and unsaturated aliphatic aldehydes between 6 and 24 carbons in length (PubMed |
| Q13671 | Ras and Rab interactor 1 | 4 | 0.46 | 0.01 | Cytoplasm |  | Ras effector protein, which may serve as an inhibitory modulator of neuronal plasticity in aversive memory formation. Can affect Ras signaling at different levels. First, by competing with RAF1 protein for binding to activated Ras. Second, by enhancing signaling from ABL1 and ABL2, which regulate cytoskeletal remodeling. Third, by activating RAB5A, possibly by functioning as a guanine nucleotide exchange factor (GEF) for RAB5A, by exchanging bound GDP for free GTP, and facilitating Ras-activated receptor endocytosis. |
| O75351 | Vacuolar protein sorting-associated protein 4B | 118 | 0.47 | 0.00 | Prevacuolar compartment membrane; Peripheral membrane protein. Late endosome membrane |  | Involved in late steps of the endosomal multivesicular bodies (MVB) pathway. Recognizes membrane-associated ESCRT-III assemblies and catalyzes their disassembly, possibly in combination with membrane fission. Redistributes the ESCRT-III components to the cytoplasm for further rounds of MVB sorting. MVBs contain intraluminal vesicles (ILVs) that are generated by invagination and scission from the limiting membrane of the endosome and mostly are delivered to lysosomes enabling degradation of membrane proteins, such as stimulated growth factor receptors, lysosomal enzymes and lipids. In conjunction with the ESCRT machinery also appears to function in topologically equivalent membrane fission events, such as the terminal stages of cytokinesis and enveloped virus budding (HIV-1 and other lentiviruses). VPS4A/B are required for the exosomal release of SDCBP, CD63 and syndecan. |
| Q06546 | GA-binding protein alpha chain | 14 | 0.10 | 0.03 | Nucleus. |  | Transcription factor capable of interacting with purine rich repeats (GA repeats). Necessary for the expression of the Adenovirus E4 gene. |
| Q8IV38 | Ankyrin repeat and MYND domain-containing protein 2 | 13 | 0.46 | 0.01 | Cell projection, cilium |  | May be involved in the trafficking of signaling proteins to the cilia. |
| Q8IVH4 | Methylmalonic aciduria type A protein, mitochondrial | 10 | 0.20 | 0.04 | Mitochondrion | Methylmalonic aciduria type cblA (MMAA) | GTPase, binds and hydrolyzes GTP. |
| Q96BJ3 | Axin interactor, dorsalization-associated protein | 9 | 0.45 | 0.00 |  |  | Acts as a ventralizing factor during embryogenesis. Inhibits axin-mediated JNK activation by binding axin and disrupting axin homodimerization. This in turn antagonizes a Wnt/beta-catenin-independent dorsalization pathway activated by AXIN/JNK-signaling. |
| P08651 | Nuclear factor 1 C-type | 6 | 0.29 | 0.02 | Nucleus. |  | Recognizes and binds the palindromic sequence 5'-TTGGCNNNNNGCCAA-3' present in viral and cellular promoters and in the origin of replication of adenovirus type 2. These proteins are individually capable of activating transcription and replication. |
| P51003 | Poly (A) polymerase alpha | 6 | 0.12 | 0.02 | Cytoplasm. Nucleus. |  | Polymerase that creates the 3'-poly(A) tail of mRNA's. Also required for the endoribonucleolytic cleavage reaction at some polyadenylation sites. May acquire specificity through interaction with a cleavage and polyadenylation specificity factor (CPSF) at its C-terminus. |
| P53582 | Methionine aminopeptidase 1 | 7 | 0.47 | 0.01 | Cytoplasm |  | Cotranslationally removes the N-terminal methionine from nascent proteins. The N-terminal methionine is often cleaved when the second residue in the primary sequence is small and uncharged (Met-Ala-, Cys, Gly, Pro, Ser, Thr, or Val). Required for normal progression through the cell cycle. |
| Q7Z7A4 | PX domain-containing protein kinase-like protein | 8 | 0.31 | 0.01 | Cytoplasm |  | Binds to and modulates brain Na,K-ATPase subunits ATP1B1 and ATP1B3 and may thereby participate in the regulation of electrical excitability and synaptic transmission. May not display kinase activity. |
| Q9BVS4 | Serine/threonine-protein kinase RIO2 | 28 | 0.28 | 0.01 | Cytoplasm |  | Serine/threonine-protein kinase involved in the final steps of cytoplasmic maturation of the 40S ribosomal subunit. Involved in export of the 40S pre-ribosome particles (pre-40S) from the nucleus to the cytoplasm. Its kinase activity is required for the release of NOB1, PNO1 and LTV1 from the late pre-40S and the processing of 18S-E pre-rRNA to the mature 18S rRNA. |
| Q9GZT4 | Serine racemase | 10 | 0.38 | 0.01 |  |  | Catalyzes the synthesis of D-serine from L-serine. D-serine is a key coagonist with glutamate at NMDA receptors. Has dehydratase activity towards both L-serine and D-serine. |
| Q6UX04 | Spliceosome-associated protein CWC27 homolog | 20 | 0.49 | 0.03 | Nucleus | Retinitis pigmentosa with or without skeletal anomalies (RPSKA) | As part of the spliceosome, plays a role in pre-mRNA splicing. |
| Q9UN70 | Protocadherin gamma-C3 | 23 | 0.44 | 0.00 | Cell membrane |  | Potential calcium-dependent cell-adhesion protein. May be involved in the establishment and maintenance of specific neuronal connections in the brain. |
| Q9Y277 | Voltage-dependent anion-selective channel protein 3 | 6 | 0.23 | 0.00 | Mitochondrion outer membrane. |  | Forms a channel through the mitochondrial outer membrane that allows diffusion of small hydrophilic molecules. |
| O14498 | Immunoglobulin superfamily containing leucine-rich repeat protein | 6 | 0.44 | 0.01 | Secreted |  |  |
| O15446 | DNA-directed RNA polymerase I subunit RPA34 | 22 | 0.27 | 0.01 | Nucleus, nucleolus |  | DNA-dependent RNA polymerase catalyzes the transcription of DNA into RNA using the four ribonucleoside triphosphates as substrates. Component of RNA polymerase I which synthesizes ribosomal RNA precursors. Isoform 1 is involved in UBTF-activated transcription, presumably at a step following PIC formation. |
| P05141 | ADP/ATP translocase 2 | 31 | 0.48 | 0.00 | Mitochondrion inner membrane; Multi-pass membrane protein. |  | Catalyzes the exchange of cytoplasmic ADP with mitochondrial ATP across the mitochondrial inner membrane. As part of the mitotic spindle-associated MMXD complex it may play a role in chromosome segregation. |
| P08962 | CD63 antigen | 16 | 0.28 | 0.02 | Cell membrane |  | Functions as cell surface receptor for TIMP1 and plays a role in the activation of cellular signaling cascades. Plays a role in the activation of ITGB1 and integrin signaling, leading to the activation of AKT, FAK/PTK2 and MAP kinases. Promotes cell survival, reorganization of the actin cytoskeleton, cell adhesion, spreading and migration, via its role in the activation of AKT and FAK/PTK2. Plays a role in VEGFA signaling via its role in regulating the internalization of KDR/VEGFR2. Plays a role in intracellular vesicular transport processes, and is required for normal trafficking of the PMEL luminal domain that is essential for the development and maturation of melanocytes. Plays a role in the adhesion of leukocytes onto endothelial cells via its role in the regulation of SELP trafficking. May play a role in mast cell degranulation in response to Ms4a2/FceRI stimulation, but not in mast cell degranulation in response to other stimuli. |
| P19447 | General transcription and DNA repair factor IIH helicase subunit XPB | 10 | 0.46 | 0.01 | Nucleus. | Xeroderma pigmentosum complementation group B (XP-B) | ATP-dependent 3'-5' DNA helicase, component of the general transcription and DNA repair factor IIH (TFIIH) core complex, which is involved in general and transcription-coupled nucleotide excision repair (NER) of damaged DNA and, when complexed to CAK, in RNA transcription by RNA polymerase II. In NER, TFIIH acts by opening DNA around the lesion to allow the excision of the damaged oligonucleotide and its replacement by a new DNA fragment. The ATPase activity of XPB/ERCC3, but not its helicase activity, is required for DNA opening. In transcription, TFIIH has an essential role in transcription initiation. |
| P29372 | DNA-3-methyladenine glycosylase | 20 | 0.44 | 0.01 | Cytoplasm |  | Hydrolysis of the deoxyribose N-glycosidic bond to excise 3-methyladenine, and 7-methylguanine from the damaged DNA polymer formed by alkylation lesions. |
| P43235 | Cathepsin K | 20 | 0.14 | 0.00 | Lysosome | Pycnodysostosis (PKND) | Thiol protease involved in osteoclastic bone resorption and may participate partially in the disorder of bone remodeling. Displays potent endoprotease activity against fibrinogen at acid pH. May play an important role in extracellular matrix degradation. Involved in the release of thyroid hormone thyroxine (T4) by limited proteolysis of TG/thyroglobulin in the thyroid follicle lumen. |
| Q8TAE8 | Growth arrest and DNA damage-inducible proteins-interacting protein 1 | 5 | 0.41 | 0.00 | Mitochondrion |  | Acts as a negative regulator of G1 to S cell cycle phase progression by inhibiting cyclin-dependent kinases. Inhibitory effects are additive with GADD45 proteins but occurs also in the absence of GADD45 proteins. Acts as a repressor of the orphan nuclear receptor NR4A1 by inhibiting AB domain-mediated transcriptional activity. May be involved in the hormone-mediated regulation of NR4A1 transcriptional activity. May play a role in mitochondrial protein synthesis. |
| Q9BSH4 | Translational activator of cytochrome c oxidase 1 | 24 | 0.03 | 0.00 | Mitochondrion | Leigh syndrome (LS) | Acts as a translational activator of mitochondrially-encoded cytochrome c oxidase 1. |
| Q9HD33 | 39S ribosomal protein L47, mitochondrial | 14 | 0.06 | 0.00 | Mitochondrion |  |  |
| P00734 | Prothrombin | 19 | 0.02 | 0.01 | Secreted, extracellular space. | Factor II deficiency (FA2D) | Thrombin, which cleaves bonds after Arg and Lys, converts fibrinogen to fibrin and activates factors V, VII, VIII, XIII, and, in complex with thrombomodulin, protein C. Functions in blood homeostasis, inflammation and wound healing. |
| P35625 | Metalloproteinase inhibitor 3 | 42 | 0.01 | 0.00 | Secreted, extracellular space, extracellular matrix. | Sorsby fundus dystrophy (SFD) | Complexes with metalloproteinases (such as collagenases) and irreversibly inactivates them by binding to their catalytic zinc cofactor. May form part of a tissue-specific acute response to remodeling stimuli. Known to act on MMP-1, MMP-2, MMP-3, MMP-7, MMP-9, MMP-13, MMP-14 and MMP-15. |
| P36404 | ADP-ribosylation factor-like protein 2 | 42 | 0.01 | 0.00 | Mitochondrion intermembrane space. Cytoplasm, cytoskeleton, microtubule organizing center, centrosome. Nucleus. Cytoplasm. |  | Small GTP-binding protein which cycles between an inactive GDP-bound and an active GTP-bound form, and the rate of cycling is regulated by guanine nucleotide exchange factors (GEF) and GTPase-activating proteins (GAP). GTP-binding protein that does not act as an allosteric activator of the cholera toxin catalytic subunit. Regulates formation of new microtubules and centrosome integrity. Prevents the TBCD-induced microtubule destruction. Participates in association with TBCD, in the disassembly of the apical junction complexes. Antagonizes the effect of TBCD on epithelial cell detachment and tight and adherens junctions disassembly. Together with ARL2, plays a role in the nuclear translocation, retention and transcriptional activity of STAT3. Component of a regulated secretory pathway involved in Ca(2+)-dependent release of acetylcholine. Required for normal progress through the cell cycle. |
| P46926 | Glucosamine-6-phosphate isomerase 1 | 22 | 0.07 | 0.01 | Cytoplasm |  | Seems to trigger calcium oscillations in mammalian eggs. These oscillations serve as the essential trigger for egg activation and early development of the embryo. |
| Q5MNZ9 | WD repeat domain phosphoinositide-interacting protein 1 | 30 | 0.01 | 0.00 | Golgi apparatus, trans-Golgi network. Endosome. Cytoplasmic vesicle, clathrin-coated vesicle. Preautophagosomal structure membrane |  | Component of the autophagy machinery that controls the major intracellular degradation process by which cytoplasmic materials are packaged into autophagosomes and delivered to lysosomes for degradation. |
| Q9Y5L4 | Mitochondrial import inner membrane translocase subunit Tim13 | 4 | 0.34 | 0.00 | Mitochondrion inner membrane |  | Mitochondrial intermembrane chaperone that participates in the import and insertion of some multi-pass transmembrane proteins into the mitochondrial inner membrane. Also required for the transfer of beta-barrel precursors from the TOM complex to the sorting and assembly machinery (SAM complex) of the outer membrane. Acts as a chaperone-like protein that protects the hydrophobic precursors from aggregation and guide them through the mitochondrial intermembrane space. The TIMM8-TIMM13 complex mediates the import of proteins such as TIMM23, SLC25A12/ARALAR1 and SLC25A13/ARALAR2, while the predominant TIMM9-TIMM10 70 kDa complex mediates the import of much more proteins. |
| Q9Y6M9 | NADH dehydrogenase [ubiquinone] 1 beta subcomplex subunit 9 | 5 | 0.27 | 0.00 | Mitochondrion inner membrane | Mitochondrial complex I deficiency, nuclear type 24 (MC1DN24) | Accessory subunit of the mitochondrial membrane respiratory chain NADH dehydrogenase (Complex I), that is believed to be not involved in catalysis. Complex I functions in the transfer of electrons from NADH to the respiratory chain. The immediate electron acceptor for the enzyme is believed to be ubiquinone. |
| O43665 | Regulator of G-protein signaling 10 | 7 | 0.39 | 0.02 |  |  | Regulates G protein-coupled receptor signaling cascades, including signaling downstream of the muscarinic acetylcholine receptor CHRM2. Inhibits signal transduction by increasing the GTPase activity of G protein alpha subunits, thereby driving them into their inactive GDP-bound form. |
| O60565 | Gremlin-1 | 9 | 0.35 | 0.01 | Secreted | Polyposis syndrome, mixed hereditary 1 (HMPS1) | Cytokine that may play an important role during carcinogenesis and metanephric kidney organogenesis, as a BMP antagonist required for early limb outgrowth and patterning in maintaining the FGF4-SHH feedback loop. Down-regulates the BMP4 signaling in a dose-dependent manner (By similarity). Antagonist of BMP2; inhibits BMP2-mediated differentiation of osteoblasts (in vitro). |
| P09012 | U1 small nuclear ribonucleoprotein A | 12 | 0.33 | 0.00 | Nucleus. |  | Component of the spliceosomal U1 snRNP, which is essential for recognition of the pre-mRNA 5' splice-site and the subsequent assembly of the spliceosome. U1 snRNP is the first snRNP to interact with pre-mRNA. This interaction is required for the subsequent binding of U2 snRNP and the U4/U6/U5 tri-snRNP. SNRPA binds stem loop II of U1 snRNA. In a snRNP-free form (SF-A) may be involved in coupled pre-mRNA splicing and polyadenylation process. May bind preferentially to the 5'-UGCAC-3' motif on RNAs. |
| P17535 | Transcription factor jun-D | 13 | 0.36 | 0.01 | Nucleus. |  | Transcription factor binding AP-1 sites. |
| P27986 | Phosphatidylinositol 3-kinase regulatory subunit alpha | 24 | 0.40 | 0.00 |  | Agammaglobulinemia 7, autosomal recessive (AGM7) | Binds to activated (phosphorylated) protein-Tyr kinases, through its SH2 domain, and acts as an adapter, mediating the association of the p110 catalytic unit to the plasma membrane. Necessary for the insulin-stimulated increase in glucose uptake and glycogen synthesis in insulin-sensitive tissues. Plays an important role in signaling in response to FGFR1, FGFR2, FGFR3, FGFR4, KITLG/SCF, KIT, PDGFRA and PDGFRB. Likewise, plays a role in ITGB2 signaling. |
| P51688 | N-sulphoglucosamine sulphohydrolase | 7 | 0.32 | 0.01 | Lysosome | Mucopolysaccharidosis 3A (MPS3A) | Catalyzes a step in lysosomal heparan sulfate degradation. |
| Q13795 | ADP-ribosylation factor-related protein 1 | 15 | 0.42 | 0.03 | Golgi apparatus |  | Trans-Golgi-associated GTPase that regulates protein sorting. Controls the targeting of ARL1 and its effector to the trans-Golgi. Required for the lipidation of chylomicrons in the intestine and required for VLDL lipidation in the liver. |
| Q16798 | NADP-dependent malic enzyme, mitochondrial | 9 | 0.33 | 0.00 | Mitochondrion matrix. |  |  |
| Q92934 | Bcl2-associated agonist of cell death | 21 | 0.46 | 0.00 | Mitochondrion outer membrane. Cytoplasm |  | Promotes cell death. Successfully competes for the binding to Bcl-X(L), Bcl-2 and Bcl-W, thereby affecting the level of heterodimerization of these proteins with BAX. Can reverse the death repressor activity of Bcl-X(L), but not that of Bcl-2 (By similarity). Appears to act as a link between growth factor receptor signaling and the apoptotic pathways. |
| Q96J01 | THO complex subunit 3 | 3 | 0.06 | 0.00 | Nucleus |  | Required for efficient export of polyadenylated RNA and spliced mRNA. Acts as component of the THO subcomplex of the TREX complex which is thought to couple mRNA transcription, processing and nuclear export, and which specifically associates with spliced mRNA and not with unspliced pre-mRNA. TREX is recruited to spliced mRNAs by a transcription-independent mechanism, binds to mRNA upstream of the exon-junction complex (EJC) and is recruited in a splicing- and cap-dependent manner to a region near the 5' end of the mRNA where it functions in mRNA export to the cytoplasm via the TAP/NFX1 pathway. The TREX complex is essential for the export of Kaposi's sarcoma-associated herpesvirus (KSHV) intronless mRNAs and infectious virus production. |
| Q96P47 | Arf-GAP with GTPase, ANK repeat and PH domain-containing protein 3 | 6 | 0.44 | 0.00 | Cytoplasm |  | GTPase-activating protein for the ADP ribosylation factor family (Potential). GTPase which may be involved in the degradation of expanded polyglutamine proteins through the ubiquitin-proteasome pathway. |
| Q9H857 | 5'-nucleotidase domain-containing protein 2 | 4 | 0.17 | 0.00 |  |  |  |
| Q9P0I2 | ER membrane protein complex subunit 3 | 6 | 0.33 | 0.00 | Membrane |  |  |
| Q9UIV1 | CCR4-NOT transcription complex subunit 7 | 9 | 0.42 | 0.00 | Nucleus. Cytoplasm, P-body |  | Has 3'-5' poly(A) exoribonuclease activity for synthetic poly(A) RNA substrate. Its function seems to be partially redundant with that of CNOT8. Catalytic component of the CCR4-NOT complex which is one of the major cellular mRNA deadenylases and is linked to various cellular processes including bulk mRNA degradation, miRNA-mediated repression, translational repression during translational initiation and general transcription regulation. During miRNA-mediated repression the complex seems also to act as translational repressor during translational initiation. Additional complex functions may be a consequence of its influence on mRNA expression. Associates with members of the BTG family such as TOB1 and BTG2 and is required for their anti-proliferative activity. |
| O14880 | Microsomal glutathione S-transferase 3 | 8 | 0.35 | 0.04 | Endoplasmic reticulum membrane |  | Catalyzes oxydation of hydroxy-fatty acids (PubMed |
| P01033 | Metalloproteinase inhibitor 1 | 8 | 0.33 | 0.00 | Secreted |  | Metalloproteinase inhibitor that functions by forming one to one complexes with target metalloproteinases, such as collagenases, and irreversibly inactivates them by binding to their catalytic zinc cofactor. Acts on MMP1, MMP2, MMP3, MMP7, MMP8, MMP9, MMP10, MMP11, MMP12, MMP13 and MMP16. Does not act on MMP14. Also functions as a growth factor that regulates cell differentiation, migration and cell death and activates cellular signaling cascades via CD63 and ITGB1. Plays a role in integrin signaling. Mediates erythropoiesis in vitro; but, unlike IL3, it is species-specific, stimulating the growth and differentiation of only human and murine erythroid progenitors. |
| P63313 | Thymosin beta-10 | 4 | 0.48 | 0.00 | Cytoplasm, cytoskeleton. |  | Plays an important role in the organization of the cytoskeleton. Binds to and sequesters actin monomers (G actin) and therefore inhibits actin polymerization. |
| Q96GC5 | 39S ribosomal protein L48, mitochondrial | 12 | 0.40 | 0.01 | Mitochondrion |  |  |
| Q9HAT2 | Sialate O-acetylesterase | 4 | 0.38 | 0.00 | Lysosome | Autoimmune disease 6 (AIS6) | Catalyzes the removal of O-acetyl ester groups from position 9 of the parent sialic acid, N-acetylneuraminic acid. |
| Q9NWS0 | PIH1 domain-containing protein 1 | 36 | 0.27 | 0.01 | Nucleus |  | Involved in the assembly of C/D box small nucleolar ribonucleoprotein (snoRNP) particles. |
| Q9NZD2 | Glycolipid transfer protein | 8 | 0.21 | 0.03 | Cytoplasm |  | Accelerates the intermembrane transfer of various glycolipids. Catalyzes the transfer of various glycosphingolipids between membranes but does not catalyze the transfer of phospholipids. May be involved in the intracellular translocation of glucosylceramides. |
| O14734 | Acyl-coenzyme A thioesterase 8 | 7 | 0.40 | 0.01 | Peroxisome matrix |  | Acyl-coenzyme A (acyl-CoA) thioesterases are a group of enzymes that catalyze the hydrolysis of acyl-CoAs to the free fatty acid and coenzyme A (CoASH), providing the potential to regulate intracellular levels of acyl-CoAs, free fatty acids and CoASH. |
| O15258 | Protein RER1 | 4 | 0.27 | 0.00 | Golgi apparatus membrane; Multi-pass membrane protein. |  | Involved in the retrieval of endoplasmic reticulum membrane proteins from the early Golgi compartment. |
| P46976 | Glycogenin-1 | 18 | 0.28 | 0.01 |  | Glycogen storage disease 15 (GSD15) | Self-glucosylates, via an inter-subunit mechanism, to form an oligosaccharide primer that serves as substrate for glycogen synthase. |
| P62304 | Small nuclear ribonucleoprotein E | 12 | 0.44 | 0.00 | Cytoplasm, cytosol | Hypotrichosis 11 (HYPT11) | Plays role in pre-mRNA splicing as core component of the SMN-Sm complex that mediates spliceosomal snRNP assembly and as component of the spliceosomal U1, U2, U4 and U5 small nuclear ribonucleoproteins (snRNPs), the building blocks of the spliceosome. |
| P69905 | Hemoglobin subunit alpha | 5 | 0.44 | 0.02 |  | Heinz body anemias (HEIBAN) | Involved in oxygen transport from the lung to the various peripheral tissues. |
| Q7LGA3 | Heparan sulfate 2-O-sulfotransferase 1 | 18 | 0.43 | 0.00 | Golgi apparatus membrane |  | Catalyzes the transfer of sulfate to the C2-position of selected hexuronic acid residues within the maturing heparan sulfate (HS). 2-O-sulfation within HS, particularly of iduronate residues, is essential for HS to participate in a variety of high-affinity ligand-binding interactions and signaling processes. Mediates 2-O-sulfation of both L-iduronyl and D-glucuronyl residues. |
| Q86Y39 | NADH dehydrogenase [ubiquinone] 1 alpha subcomplex subunit 11 | 6 | 0.43 | 0.00 | Mitochondrion inner membrane | Mitochondrial complex I deficiency, nuclear type 14 (MC1DN14) | Accessory subunit of the mitochondrial membrane respiratory chain NADH dehydrogenase (Complex I), that is believed not to be involved in catalysis. Complex I functions in the transfer of electrons from NADH to the respiratory chain. The immediate electron acceptor for the enzyme is believed to be ubiquinone. |
| Q8NB37 | Glutamine amidotransferase-like class 1 domain-containing protein 1 | 5 | 0.41 | 0.03 | Secreted |  |  |
| Q96K37 | Solute carrier family 35 member E1 | 3 | 0.41 | 0.01 | Membrane |  | Putative transporter. |
| Q96S97 | Myeloid-associated differentiation marker | 3 | 0.50 | 0.04 | Membrane |  |  |
| Q9Y6G5 | COMM domain-containing protein 10 | 3 | 0.43 | 0.00 | Cytoplasm |  | May modulate activity of cullin-RING E3 ubiquitin ligase (CRL) complexes. |
| P07311 | Acylphosphatase-1 | 9 | 0.38 | 0.00 |  |  | Its physiological role is not yet clear. |
| P08236 | Beta-glucuronidase | 11 | 0.17 | 0.02 | Lysosome. | Mucopolysaccharidosis 7 (MPS7) | Plays an important role in the degradation of dermatan and keratan sulfates. |
| P48059 | LIM and senescent cell antigen-like-containing domain protein 1 | 12 | 0.43 | 0.00 | Cell junction, focal adhesion. Cell membrane; Peripheral membrane protein; Cytoplasmic side. |  | Adapter protein in a cytoplasmic complex linking beta-integrins to the actin cytoskeleton, bridges the complex to cell surface receptor tyrosine kinases and growth factor receptors. Involved in the regulation of cell survival, cell proliferation and cell differentiation. |
| P52758 | 2-iminobutanoate/2-iminopropanoate deaminase | 14 | 0.33 | 0.04 | Cytoplasm |  | Catalyzes the hydrolytic deamination of enamine/imine intermediates that form during the course of normal metabolism. May facilitate the release of ammonia from these potentially toxic reactive metabolites, reducing their impact on cellular components. It may act on enamine/imine intermediates formed by several types of pyridoxal-5'-phosphate-dependent dehydratases including L-threonine dehydratase. |
| Q14624 | Inter-alpha-trypsin inhibitor heavy chain H4 | 8 | 0.21 | 0.02 | Secreted. |  | Type II acute-phase protein (APP) involved in inflammatory responses to trauma. May also play a role in liver development or regeneration. |
| Q15035 | Translocating chain-associated membrane protein 2 | 4 | 0.24 | 0.04 | Membrane |  | Necessary for collagen type I synthesis. May couple the activity of the ER Ca(2+) pump SERCA2B with the activity of the translocon. This coupling may increase the local Ca(2+) concentration at the site of collagen synthesis, and a high Ca(2+) concentration may be necessary for the function of molecular chaperones involved in collagen folding. Required for proper insertion of the first transmembrane helix N-terminus of TM4SF20 into the ER lumen, may act as a ceramide sensor for regulated alternative translocation (RAT). |
| Q8IYB5 | Stromal membrane-associated protein 1 | 17 | 0.23 | 0.00 | Cell membrane |  | GTPase activating protein that acts on ARF6. Plays a role in clathrin-dependent endocytosis. May play a role in erythropoiesis. |
| Q8N9N2 | Activating signal cointegrator 1 complex subunit 1 | 12 | 0.30 | 0.00 | Nucleus | Barrett esophagus (BE) | Plays a role in DNA damage repair as component of the ASCC complex. |
| Q96C90 | Protein phosphatase 1 regulatory subunit 14B | 12 | 0.45 | 0.02 | Cytoplasm |  | Inhibitor of PPP1CA. Has over 50-fold higher inhibitory activity when phosphorylated. |
| Q9BTE7 | DCN1-like protein 5 | 17 | 0.41 | 0.00 |  |  |  |
| Q9H4B6 | Protein salvador homolog 1 | 15 | 0.40 | 0.00 | Nucleus |  | Regulator of STK3/MST2 and STK4/MST1 in the Hippo signaling pathway which plays a pivotal role in organ size control and tumor suppression by restricting proliferation and promoting apoptosis. The core of this pathway is composed of a kinase cascade wherein STK3/MST2 and STK4/MST1, in complex with its regulatory protein SAV1, phosphorylates and activates LATS1/2 in complex with its regulatory protein MOB1, which in turn phosphorylates and inactivates YAP1 oncoprotein and WWTR1/TAZ. Phosphorylation of YAP1 by LATS1/2 inhibits its translocation into the nucleus to regulate cellular genes important for cell proliferation, cell death, and cell migration. SAV1 is required for STK3/MST2 and STK4/MST1 activation and promotes cell-cycle exit and terminal differentiation in developing epithelial tissues. Plays a role in centrosome disjunction by regulating the localization of NEK2 to centrosomes, and its ability to phosphorylate CROCC and CEP250. In conjunction with STK3/MST2, activates the transcriptional activity of ESR1 through the modulation of its phosphorylation. |
| Q9Y5R8 | Trafficking protein particle complex subunit 1 | 42 | 0.32 | 0.00 | Golgi apparatus, cis-Golgi network |  | May play a role in vesicular transport from endoplasmic reticulum to Golgi. |
| P02788 | Lactotransferrin | 14 | 0.46 | 0.04 |  |  | Transferrins are iron binding transport proteins which can bind two Fe(3+) ions in association with the binding of an anion, usually bicarbonate. |
| P05155 | Plasma protease C1 inhibitor | 7 | 0.37 | 0.01 | Secreted. | Hereditary angioedema (HAE) | Activation of the C1 complex is under control of the C1-inhibitor. It forms a proteolytically inactive stoichiometric complex with the C1r or C1s proteases. May play a potentially crucial role in regulating important physiological pathways including complement activation, blood coagulation, fibrinolysis and the generation of kinins. Very efficient inhibitor of FXIIa. Inhibits chymotrypsin and kallikrein. |
| P17676 | CCAAT/enhancer-binding protein beta | 6 | 0.43 | 0.02 | Nucleus |  | Important transcription factor regulating the expression of genes involved in immune and inflammatory responses. |
| Q02447 | Transcription factor Sp3 | 6 | 0.45 | 0.00 | Nucleus. Nucleus, PML body. |  | Transcriptional factor that can act as an activator or repressor depending on isoform and/or post-translational modifications. Binds to GT and GC boxes promoter elements. Competes with SP1 for the GC-box promoters. Weak activator of transcription but can activate a number of genes involved in different processes such as cell-cycle regulation, hormone-induction and house-keeping. |
| Q6P1X6 | UPF0598 protein C8orf82 | 26 | 0.45 | 0.00 |  |  |  |
| Q8IZQ5 | Selenoprotein H | 11 | 0.34 | 0.00 |  |  | May be involved in a redox-related process. |
| Q8N9A8 | Nuclear envelope phosphatase-regulatory subunit 1 | 5 | 0.43 | 0.00 | Nucleus membrane |  | Forms with the serine/threonine protein phosphatase CTDNEP1 an active complex which dephosphorylates and may activate LPIN1 and LPIN2. LPIN1 and LPIN2 are phosphatidate phosphatases that catalyze the conversion of phosphatidic acid to diacylglycerol and control the metabolism of fatty acids at different levels. May indirectly modulate the lipid composition of nuclear and/or endoplasmic reticulum membranes and be required for proper nuclear membrane morphology and/or dynamics. May also indirectly regulate the production of lipid droplets and triacylglycerol. |
| Q8WUR7 | UPF0235 protein C15orf40 | 33 | 0.31 | 0.05 |  |  |  |
| Q9NRP0 | Oligosaccharyltransferase complex subunit OSTC | 2 | 0.20 | 0.00 | Endoplasmic reticulum |  | Subunit of the oligosaccharyl transferase (OST) complex that catalyzes the initial transfer of a defined glycan (Glc(3)Man(9)GlcNAc(2) in eukaryotes) from the lipid carrier dolichol-pyrophosphate to an asparagine residue within an Asn-X-Ser/Thr consensus motif in nascent polypeptide chains, the first step in protein N-glycosylation. N-glycosylation occurs cotranslationally and the complex associates with the Sec61 complex at the channel-forming translocon complex that mediates protein translocation across the endoplasmic reticulum (ER). All subunits are required for a maximal enzyme activity. May be involved in N-glycosylation of APP (amyloid-beta precursor protein). Can modulate gamma-secretase cleavage of APP by enhancing endoprotelysis of PSEN1. |
| Q9NYB9 | Abl interactor 2 | 8 | 0.48 | 0.00 | Cytoplasm |  | Regulator of actin cytoskeleton dynamics underlying cell motility and adhesion. Functions as a component of the WAVE complex, which activates actin nucleating machinery Arp2/3 to drive lamellipodia formation. |
| Q9UJJ9 | N-acetylglucosamine-1-phosphotransferase subunit gamma | 14 | 0.47 | 0.02 | Secreted | Mucolipidosis type III complementation group C (MLIIIC) | Non-catalytic subunit of the N-acetylglucosamine-1-phosphotransferase complex, an enzyme that catalyzes the formation of mannose 6-phosphate (M6P) markers on high mannose type oligosaccharides in the Golgi apparatus. Binds and presents the high mannose glycans of the acceptor to the catalytic alpha and beta subunits (GNPTAB). Enhances the rate of N-acetylglucosamine-1-phosphate transfer to the oligosaccharides of acid hydrolase acceptors. |
| Q9Y385 | Ubiquitin-conjugating enzyme E2 J1 | 7 | 0.12 | 0.00 | Endoplasmic reticulum membrane |  | Catalyzes the covalent attachment of ubiquitin to other proteins. Functions in the selective degradation of misfolded membrane proteins from the endoplasmic reticulum (ERAD). |
| Q9Y399 | 28S ribosomal protein S2, mitochondrial | 5 | 0.45 | 0.00 | Mitochondrion | Combined oxidative phosphorylation deficiency 36 (COXPD36) | Required for mitoribosome formation and stability, and mitochondrial translation. |
| Q9Y3B2 | Exosome complex component CSL4 | 3 | 0.38 | 0.00 | Nucleus, nucleolus |  | Non-catalytic component of the RNA exosome complex which has 3'->5' exoribonuclease activity and participates in a multitude of cellular RNA processing and degradation events. In the nucleus, the RNA exosome complex is involved in proper maturation of stable RNA species such as rRNA, snRNA and snoRNA, in the elimination of RNA processing by-products and non-coding 'pervasive' transcripts, such as antisense RNA species and promoter-upstream transcripts (PROMPTs), and of mRNAs with processing defects, thereby limiting or excluding their export to the cytoplasm. The RNA exosome may be involved in Ig class switch recombination (CSR) and/or Ig variable region somatic hypermutation (SHM) by targeting AICDA deamination activity to transcribed dsDNA substrates. In the cytoplasm, the RNA exosome complex is involved in general mRNA turnover and specifically degrades inherently unstable mRNAs containing AU-rich elements (AREs) within their 3' untranslated regions, and in RNA surveillance pathways, preventing translation of aberrant mRNAs. It seems to be involved in degradation of histone mRNA. The catalytic inactive RNA exosome core complex of 9 subunits (Exo-9) is proposed to play a pivotal role in the binding and presentation of RNA for ribonucleolysis, and to serve as a scaffold for the association with catalytic subunits and accessory proteins or complexes. EXOSC1 as peripheral part of the Exo-9 complex stabilizes the hexameric ring of RNase PH-domain subunits through contacts with EXOSC6 and EXOSC8. |
| Q9Y3Q3 | Transmembrane emp24 domain-containing protein 3 | 8 | 0.37 | 0.01 | Endoplasmic reticulum-Golgi intermediate compartment membrane |  | Potential role in vesicular protein trafficking, mainly in the early secretory pathway. Contributes to the coupled localization of TMED2 and TMED10 in the cis-Golgi network. |
| O43731 | ER lumen protein-retaining receptor 3 | 4 | 0.39 | 0.02 | Endoplasmic reticulum membrane |  | Receptor for the C-terminal sequence motif K-D-E-L that is present on endoplasmic reticulum resident proteins and that mediates their recycling from the Golgi back to the endoplasmic reticulum. |
| O43819 | Protein SCO2 homolog, mitochondrial | 10 | 0.29 | 0.03 | Mitochondrion inner membrane | Cardioencephalomyopathy, fatal infantile, due to cytochrome c oxidase deficiency 1 (CEMCOX1) | Copper metallochaperone essential for the synthesis and maturation of cytochrome c oxidase subunit II (MT-CO2/COX2). Involved in transporting copper to the Cu(A) site on MT-CO2/COX2. |
| O95070 | Protein YIF1A | 5 | 0.34 | 0.00 | Endoplasmic reticulum membrane |  | Possible role in transport between endoplasmic reticulum and Golgi. |
| P00156 | Cytochrome b (Complex III subunit 3) | 4 | 0.46 | 0.00 | Mitochondrion inner membrane |  | Component of the ubiquinol-cytochrome c reductase complex (complex III or cytochrome b-c1 complex) that is part of the mitochondrial respiratory chain. The b-c1 complex mediates electron transfer from ubiquinol to cytochrome c. Contributes to the generation of a proton gradient across the mitochondrial membrane that is then used for ATP synthesis. |
| P05412 | Transcription factor AP-1 | 19 | 0.40 | 0.03 | Nucleus. |  | Transcription factor that recognizes and binds to the enhancer heptamer motif 5'-TGA[CG]TCA-3'. |
| P56377 | AP-1 complex subunit sigma-2 | 35 | 0.19 | 0.05 | Golgi apparatus. Cytoplasmic vesicle membrane; Peripheral membrane protein; Cytoplasmic side. Membrane, clathrin-coated pit. | Pettigrew syndrome (PGS) | Subunit of clathrin-associated adaptor protein complex 1 that plays a role in protein sorting in the late-Golgi/trans-Golgi network (TGN) and/or endosomes. The AP complexes mediate both the recruitment of clathrin to membranes and the recognition of sorting signals within the cytosolic tails of transmembrane cargo molecules. |
| Q12974 | Protein tyrosine phosphatase type IVA 2 | 8 | 0.20 | 0.00 | Cell membrane. Early endosome. Cytoplasm. |  | Protein tyrosine phosphatase which stimulates progression from G1 into S phase during mitosis. Promotes tumors. Inhibits geranylgeranyl transferase type II activity by blocking the association between RABGGTA and RABGGTB. |
| Q15796 | Mothers against decapentaplegic homolog 2 | 3 | 0.47 | 0.01 | Cytoplasm |  | Receptor-regulated SMAD (R-SMAD) that is an intracellular signal transducer and transcriptional modulator activated by TGF-beta (transforming growth factor) and activin type 1 receptor kinases. Binds the TRE element in the promoter region of many genes that are regulated by TGF-beta and, on formation of the SMAD2/SMAD4 complex, activates transcription. May act as a tumor suppressor in colorectal carcinoma. Positively regulates PDPK1 kinase activity by stimulating its dissociation from the 14-3-3 protein YWHAQ which acts as a negative regulator. |
| Q16540 | 39S ribosomal protein L23, mitochondrial | 4 | 0.22 | 0.00 | Mitochondrion |  |  |
| Q96IX5 | ATP synthase membrane subunit DAPIT, mitochondrial | 4 | 0.09 | 0.00 | Mitochondrion membrane |  | Mitochondrial membrane ATP synthase (F(1)F(0) ATP synthase or Complex V) produces ATP from ADP in the presence of a proton gradient across the membrane which is generated by electron transport complexes of the respiratory chain. F-type ATPases consist of two structural domains, F(1) - containing the extramembraneous catalytic core and F(0) - containing the membrane proton channel, linked together by a central stalk and a peripheral stalk. During catalysis, ATP synthesis in the catalytic domain of F(1) is coupled via a rotary mechanism of the central stalk subunits to proton translocation (Probable). Minor subunit required to maintain the ATP synthase population in the mitochondria. |
| Q9NVA1 | Ubiquinol-cytochrome-c reductase complex assembly factor 1 | 4 | 0.27 | 0.01 | Mitochondrion inner membrane |  | Required for the assembly of the ubiquinol-cytochrome c reductase complex (mitochondrial respiratory chain complex III or cytochrome b-c1 complex). Involved in cytochrome b translation and/or stability. |
| Q15125 | 3-beta-hydroxysteroid-Delta | 3 | 0.49 | 0.00 | Endoplasmic reticulum membrane | Chondrodysplasia punctata 2, X-linked dominant (CDPX2) | Catalyzes the conversion of Delta(8)-sterols to their corresponding Delta(7)-isomers. |
| Q99643 | Succinate dehydrogenase cytochrome b560 subunit, mitochondrial | 13 | 0.48 | 0.01 | Mitochondrion inner membrane; Multi-pass membrane protein. | Paragangliomas 3 (PGL3) | Membrane-anchoring subunit of succinate dehydrogenase (SDH) that is involved in complex II of the mitochondrial electron transport chain and is responsible for transferring electrons from succinate to ubiquinone (coenzyme Q). |
| Q9H8M2 | Bromodomain-containing protein 9 | 11 | 0.49 | 0.00 |  |  | Plays a role in chromatin remodeling and regulation of transcription. |
| Q9UDW1 | Cytochrome b-c1 complex subunit 9 | 9 | 0.34 | 0.00 | Mitochondrion inner membrane |  | Component of the ubiquinol-cytochrome c oxidoreductase, a multisubunit transmembrane complex that is part of the mitochondrial electron transport chain which drives oxidative phosphorylation. The respiratory chain contains 3 multisubunit complexes succinate dehydrogenase (complex II, CII), ubiquinol-cytochrome c oxidoreductase (cytochrome b-c1 complex, complex III, CIII) and cytochrome c oxidase (complex IV, CIV), that cooperate to transfer electrons derived from NADH and succinate to molecular oxygen, creating an electrochemical gradient over the inner membrane that drives transmembrane transport and the ATP synthase. The cytochrome b-c1 complex catalyzes electron transfer from ubiquinol to cytochrome c, linking this redox reaction to translocation of protons across the mitochondrial inner membrane, with protons being carried across the membrane as hydrogens on the quinol. In the process called Q cycle, 2 protons are consumed from the matrix, 4 protons are released into the intermembrane space and 2 electrons are passed to cytochrome c. |
