# Supplementary material for: Protein signature of human skin fibroblasts allows the study of the molecular etiology of rare neurological diseases
Source: Orphanet J Rare Dis. 2021 Feb 9;16:73. doi: 10.1186/s13023-020-01669-1 (PMC7874489; doi:10.1186/s13023-020-01669-1)
Supplement: Supplementary file 4 — Additional file 4: Table 4. List of proteins affected by bi-allelic c.762delC AAAS mutation in enriched nuclear fraction of human skin fibroblasts: ten proteins were found to be increased whereas nine were decreased in the nuclear fractions of patient-derived cells. For each protein, the predicted function as well as the subcellular localization (www.uniprot.org) is provided. [file 13023_2020_1669_MOESM4_ESM.docx]

| **Accession** | **Protein names** | **Unique peptides** | **Fold change** | **Pvalue** | **Subcellular location** | **Involvment in disease** | **Function** |
| --- | --- | --- | --- | --- | --- | --- | --- |
| Q99715 | Collagen alpha-1(XII) chain | 3 | 7.33 | 0.01 | extracellular matrix | Ullrich congenital muscular dystrophy 2 (UCMD2)/ Bethlem myopathy 2 (BTHLM2) | Type XII collagen interacts with type I collagen-containing fibrils, the COL1 domain could be associated with the surface of the fibrils, and the COL2 and NC3 domains may be localized in the perifibrillar matrix. |
| Q13642 | Four and a half LIM domains protein 1 | 2 | 5.53 | 0.00 | Cytoplasm, Nucleus | Emery-Dreifuss muscular dystrophy 6, X-linked (EDMD6)/Scapuloperoneal myopathy, X-linked dominant (SPM)/Myopathy, X-linked, with postural muscle atrophy (XMPMA)/Reducing body myopathy, X-linked 1A, severe, with infantile or early childhood onset (RBMX1A)/Reducing body myopathy, X-linked 1B, with late childhood or adult onset (RBMX1B)/Uruguay faciocardiomusculoskeletal syndrome (FCMSU) | May have an involvement in muscle development or hypertrophy. |
| P04216 | Thy-1 membrane glycoprotein | 5 | 5.10 | 0.03 | Cell membrane, GPI-anchor |  | May play a role in cell-cell or cell-ligand interactions during synaptogenesis and other events in the brain. |
| Q15063 | Periostin | 3 | 3.87 | 0.00 | Golgi apparatus, extracellular matrix, secreted |  | Induces cell attachment and spreading and plays a role in cell adhesion. Enhances incorporation of BMP1 in the fibronectin matrix of connective tissues, and subsequent proteolytic activation of lysyl oxidase LOX. |
| Q6UXB8 | Peptidase inhibitor 16 | 2 | 3.76 | 0.00 | Secreted |  | May inhibit cardiomyocyte growth. |
| Q16270 | Insulin-like growth factor-binding protein 7 | 3 | 3.42 | 0.00 | Secreted | Retinal arterial macroaneurysm with supravalvular pulmonic stenosis (RAMSVPS) | Binds IGF-I and IGF-II with a relatively low affinity. Stimulates prostacyclin (PGI2) production. Stimulates cell adhesion. |
| P40261 | Nicotinamide N-methyltransferase | 5 | 2.83 | 0.00 | Cytoplasm |  | Catalyzes the N-methylation of nicotinamide and other pyridines to form pyridinium ions. This activity is important for biotransformation of many drugs and xenobiotic compounds. |
| P02452 | Collagen alpha-1(I) chain | 32 | 2.80 | 0.00 | extracellular matrix PROSITE-ProRule annotation | Caffey disease (CAFFD)/Ehlers-Danlos syndrome, classic type, 1 (EDSCL1)/Ehlers-Danlos syndrome, arthrochalasia type, 1 (EDSARTH1)/Osteogenesis imperfecta 1-4 (OI1-4)/Osteoporosis (OSTEOP) | Type I collagen is a member of group I collagen (fibrillar forming collagen). |
| Q96CG8 | Collagen triple helix repeat-containing protein 1 | 65 | 2.75 | 0.00 | extracellular matrix | Barrett esophagus (BE) | May act as a negative regulator of collagen matrix deposition. |
| P23381 | Tryptophan--tRNA ligase, cytoplasmic | 2 | 2.71 | 0.00 | Cytoplasm | Neuronopathy, distal hereditary motor, 9 (HMN9) | Isoform 1, isoform 2 and T1-TrpRS have aminoacylation activity while T2-TrpRS lacks it. Isoform 2, T1-TrpRS and T2-TrpRS possess angiostatic activity whereas isoform 1 lacks it. T2-TrpRS inhibits fluid shear stress-activated responses of endothelial cells. Regulates ERK, Akt, and eNOS activation pathways that are associated with angiogenesis, cytoskeletal reorganization and shear stress-responsive gene expression. |
| P18859 | ATP synthase-coupling factor 6 | 3 | 2.53 | 0.04 | Mitochondrion |  | Mitochondrial membrane ATP synthase (F1F0 ATP synthase or Complex V) produces ATP from ADP in the presence of a proton gradient across the membrane which is generated by electron transport complexes of the respiratory chain. F-type ATPases consist of two structural domains, F1 - containing the extramembraneous catalytic core and F0 - containing the membrane proton channel, linked together by a central stalk and a peripheral stalk. During catalysis, ATP synthesis in the catalytic domain of F1 is coupled via a rotary mechanism of the central stalk subunits to proton translocation. Part of the complex F0 domain and the peripheric stalk, which acts as a stator to hold the catalytic alpha3beta3 subcomplex and subunit a/ATP6 static relative to the rotary elements. Also involved in the restoration of oligomycin-sensitive ATPase activity to depleted F1-F0 complexes. |
| P55145 | Mesencephalic astrocyte-derived neurotrophic factor | 4 | 2.48 | 0.01 | Secreted, ER, Sarcoplasm |  | Selectively promotes the survival of dopaminergic neurons of the ventral mid-brain. Modulates GABAergic transmission to the dopaminergic neurons of the substantia nigra. Enhances spontaneous, as well as evoked, GABAergic inhibitory postsynaptic currents in dopaminergic neurons. Inhibits cell proliferation and endoplasmic reticulum (ER) stress-induced cell death. Retained in the ER/sarcoplasmic reticulum (SR) through association with the endoplasmic reticulum chaperone protein HSPA5 under normal conditions. Up-regulated and secreted by the ER/SR in response to ER stress and hypoxia. Following secretion by the ER/SR, directly binds to 3-O-sulfogalactosylceramide, a lipid sulfatide in the outer cell membrane of target cells. Sulfatide binding promotes its cellular uptake by endocytosis, and is required for its role in alleviating ER stress and cell toxicity under hypoxic and ER stress conditions. |
| Q9NYL4 | Peptidyl-prolyl cis-trans isomerase FKBP11 | 75 | 2.44 | 0.00 | Membrane Curated; Single-pass membrane protein Curated |  | PPIases accelerate the folding of proteins during protein synthesis. |
| O43175 | D-3-phosphoglycerate dehydrogenase | 8 | 2.44 | 0.00 |  | Phosphoglycerate dehydrogenase deficiency (PHGDHD)/Neu-Laxova syndrome 1 (NLS1) | Catalyzes the reversible oxidation of 3-phospho-D-glycerate to 3-phosphonooxypyruvate, the first step of the phosphorylated L-serine biosynthesis pathway. Also catalyzes the reversible oxidation of 2-hydroxyglutarate to 2-oxoglutarate and the reversible oxidation of (S)-malate to oxaloacetate. |
| Q99873 | Protein arginine N-methyltransferase 1 | 3 | 2.36 | 0.00 | Nucleus, nucleoplasm, cytoplasm |  | Arginine methyltransferase that methylates (mono and asymmetric dimethylation) the guanidino nitrogens of arginyl residues present in proteins such as ESR1, histone H2, H3 and H4, ILF3, HNRNPA1, HNRNPD, NFATC2IP, SUPT5H, TAF15, EWS, HABP4 and SERBP1. Constitutes the main enzyme that mediates monomethylation and asymmetric dimethylation of histone H4 'Arg-4' (H4R3me1 and H4R3me2a, respectively), a specific tag for epigenetic transcriptional activation. May be involved in the regulation of TAF15 transcriptional activity, act as an activator of estrogen receptor (ER)-mediated transactivation, play a key role in neurite outgrowth and act as a negative regulator of megakaryocytic differentiation, by modulating p38 MAPK pathway. Methylates RBM15, promoting ubiquitination and degradation of RBM15. Methylates FOXO1 and retains it in the nucleus increasing its transcriptional activity. Methylates CHTOP and this methylation is critical for its 5-hydroxymethylcytosine (5hmC)-binding activity. Methylates H4R3 in genes involved in glioblastomagenesis in a CHTOP- and/or TET1-dependent manner. |
| P06748 | Nucleophosmin | 2 | 2.34 | 0.00 | nucleolus, nucleoplasm, centrosome | A chromosomal aberration involving NPM1 is found in a form of non-Hodgkin lymphoma. Translocation t(2;5)(p23;q35) with ALK. | Involved in diverse cellular processes such as ribosome biogenesis, centrosome duplication, protein chaperoning, histone assembly, cell proliferation, and regulation of tumor suppressors p53/TP53 and ARF. Binds ribosome presumably to drive ribosome nuclear export. Associated with nucleolar ribonucleoprotein structures and bind single-stranded nucleic acids. Acts as a chaperonin for the core histones H3, H2B and H4. Stimulates APEX1 endonuclease activity on apurinic/apyrimidinic (AP) double-stranded DNA but inhibits APEX1 endonuclease activity on AP single-stranded RNA. May exert a control of APEX1 endonuclease activity within nucleoli devoted to repair AP on rDNA and the removal of oxidized rRNA molecules. In concert with BRCA2, regulates centrosome duplication. Regulates centriole duplication: phosphorylation by PLK2 is able to trigger centriole replication. Negatively regulates the activation of EIF2AK2/PKR and suppresses apoptosis through inhibition of EIF2AK2/PKR autophosphorylation. Antagonizes the inhibitory effect of ATF5 on cell proliferation and relieves ATF5-induced G2/M blockade. In complex with MYC enhances the transcription of MYC target genes. |
| Q16666 | Gamma-interferon-inducible protein 16 | 3 | 2.19 | 0.00 | Nucleus |  | Binds double-stranded DNA. Binds preferentially to supercoiled DNA and cruciform DNA structures. Seems to be involved in transcriptional regulation. May function as a transcriptional repressor. Could have a role in the regulation of hematopoietic differentiation through activation of unknown target genes. Controls cellular proliferation by modulating the functions of cell cycle regulatory factors including p53/TP53 and the retinoblastoma protein. May be involved in TP53-mediated transcriptional activation by enhancing TP53 sequence-specific DNA binding and modulating TP53 phosphorylation status. Seems to be involved in energy-level-dependent activation of the ATM/ AMPK/TP53 pathway coupled to regulation of autophagy. May be involved in regulation of TP53-mediated cell death also involving BRCA1. May be involved in the senescence of prostate epithelial cells. Involved in innate immune response by recognizing viral dsDNA in the cytosol and probably in the nucleus. After binding to viral DNA in the cytoplasm recruits TMEM173/STING and mediates the induction of IFN-beta. Has anti-inflammatory activity and inhibits the activation of the AIM2 inflammasome, probably via association with AIM2. Proposed to bind viral DNA in the nucleus, such as of Kaposi's sarcoma-associated herpesvirus, and to induce the formation of nuclear caspase-1-activating inflammasome formation via association with PYCARD. Inhibits replication of herpesviruses such as human cytomegalovirus (HCMV) probably by interfering with promoter recruitment of members of the Sp1 family of transcription factors. Necessary to activate the IRF3 signaling cascade during human herpes simplex virus 1 (HHV-1) infection and promotes the assembly of heterochromatin on herpesviral DNA and inhibition of viral immediate-early gene expression and replication. Involved in the MTA1-mediated epigenetic regulation of ESR1 expression in breast cancer. |
| P50395 | Rab GDP dissociation inhibitor beta | 4 | 2.19 | 0.00 | Cytoplasm, membrane |  | Regulates the GDP/GTP exchange reaction of most Rab proteins by inhibiting the dissociation of GDP from them, and the subsequent binding of GTP to them. |
| P20908 | Collagen alpha-1(V) chain | 4 | 2.18 | 0.01 | extracellular matrix PROSITE-ProRule annotation | Ehlers-Danlos syndrome, classic type, 1 (EDSCL1) | Type V collagen is a member of group I collagen (fibrillar forming collagen). It is a minor connective tissue component of nearly ubiquitous distribution. Type V collagen binds to DNA, heparan sulfate, thrombospondin, heparin, and insulin. |
| P08123 | Collagen alpha-2(I) chain | 8 | 2.12 | 0.00 | extracellular matrix | Ehlers-Danlos syndrome, arthrochalasia type, 2 (EDSARTH2)/Osteogenesis imperfecta 1-4 (OI1-4) | Type I collagen is a member of group I collagen (fibrillar forming collagen). |
| Q92896 | Golgi apparatus protein 1 | 32 | 2.11 | 0.00 | Golgi apparatus membrane, microtubule |  | Binds fibroblast growth factor and E-selectin (cell-adhesion lectin on endothelial cells mediating the binding of neutrophils). |
| Q14192 | Four and a half LIM domains protein 2 | 3 | 2.10 | 0.00 | Cytoplasm, nucleus |  | May function as a molecular transmitter linking various signaling pathways to transcriptional regulation. Negatively regulates the transcriptional repressor E4F1 and may function in cell growth. Inhibits the transcriptional activity of FOXO1 and its apoptotic function by enhancing the interaction of FOXO1 with SIRT1 and FOXO1 deacetylation. Negatively regulates the calcineurin/NFAT signaling pathway in cardiomyocytes. |
| P60981 | Destrin | 8 | 2.07 | 0.00 |  |  | Actin-depolymerizing protein. Severs actin filaments (F-actin) and binds to actin monomers (G-actin). Acts in a pH-independent manner. |
| P02751 | Fibronectin | 7 | 2.04 | 0.00 | extracellular matrix | Glomerulopathy with fibronectin deposits 2 (GFND2)/Spondylometaphyseal dysplasia, corner fracture type (SMDCF) | Fibronectins bind cell surfaces and various compounds including collagen, fibrin, heparin, DNA, and actin. Fibronectins are involved in cell adhesion, cell motility, opsonization, wound healing, and maintenance of cell shape. Involved in osteoblast compaction through the fibronectin fibrillogenesis cell-mediated matrix assembly process, essential for osteoblast mineralization. Participates in the regulation of type I collagen deposition by osteoblasts. |
| Q96CS3 | FAS-associated factor 2 | 6 | 2.04 | 0.01 | Cytoplasm, lipid droplet, ER |  | Plays an important role in endoplasmic reticulum-associated degradation (ERAD) that mediates ubiquitin-dependent degradation of misfolded endoplasmic reticulum proteins. By controlling the steady-state expression of the IGF1R receptor, indirectly regulates the insulin-like growth factor receptor signaling pathway. Involved in inhibition of lipid droplet degradation by binding to phospholipase PNPL2 and inhibiting its activity by promoting dissociation of PNPL2 from its endogenous activator, ABHD5 which inhibits the rate of triacylglycerol hydrolysis. |
| Q13488 | V-type proton ATPase 116 kDa subunit a isoform 3 | 2 | 2.04 | 0.04 | Membrane | Osteopetrosis, autosomal recessive 1 (OPTB1) | Part of the proton channel of V-ATPases. Seems to be directly involved in T-cell activation. |
| P09936 | Ubiquitin carboxyl-terminal hydrolase isoenzym L1 | 2 | 2.03 | 0.00 | Cytoplasm, ER | Parkinson disease 5 (PARK5)/Spastic paraplegia 79, autosomal recessive (SPG79) | Ubiquitin-protein hydrolase involved both in the processing of ubiquitin precursors and of ubiquitinated proteins (Probable). This enzyme is a thiol protease that recognizes and hydrolyzes a peptide bond at the C-terminal glycine of ubiquitin. Also binds to free monoubiquitin and may prevent its degradation in lysosomes. The homodimer may have ATP-independent ubiquitin ligase activity. |
| Q969G5 | Caveolae-associated protein 3 | 9 | 0.50 | 0.00 | Cytoplasm, caveola |  | Regulates the traffic and/or budding of caveolae. Plays a role in caveola formation in a tissue-specific manner. Required for the formation of caveolae in smooth muscle but not in the lung and heart endothelial cells. Regulates the equilibrium between cell surface-associated and cell surface-dissociated caveolae by promoting the rapid release of caveolae from the cell surface. Plays a role in the regulation of the circadian clock. Modulates the period length and phase of circadian gene expression and also regulates expression and interaction of the core clock components PER1/2 and CRY1/2. |
| P30038 | Delta-1-pyrroline-5-carboxylate dehydrogenase | 2 | 0.50 | 0.03 | Mitochondrion matrix | Hyperprolinemia 2 (HYRPRO2) | Irreversible conversion of delta-1-pyrroline-5-carboxylate (P5C), derived either from proline or ornithine, to glutamate. This is a necessary step in the pathway interconnecting the urea and tricarboxylic acid cycles. The preferred substrate is glutamic gamma-semialdehyde, other substrates include succinic, glutaric and adipic semialdehydes. |
| Q15121 | Astrocytic phosphoprotein PEA-15 | 4 | 0.48 | 0.00 | Cytoplasm |  | Blocks Ras-mediated inhibition of integrin activation and modulates the ERK MAP kinase cascade. Inhibits RPS6KA3 activities by retaining it in the cytoplasm. Inhibits both TNFRSF6- and TNFRSF1A-mediated CASP8 activity and apoptosis. Regulates glucose transport by controlling both the content of SLC2A1 glucose transporters on the plasma membrane and the insulin-dependent trafficking of SLC2A4 from the cell interior to the surface. |
| P30041 | Peroxiredoxin-6 | 22 | 0.48 | 0.01 | Cytoplasm, lysosome |  | Thiol-specific peroxidase that catalyzes the reduction of hydrogen peroxide and organic hydroperoxides to water and alcohols, respectively. Can reduce H2O2 and short chain organic, fatty acid, and phospholipid hydroperoxides. Also has phospholipase activity, and can therefore either reduce the oxidized sn-2 fatty acyl grup of phospholipids (peroxidase activity) or hydrolyze the sn-2 ester bond of phospholipids (phospholipase activity). These activities are dependent on binding to phospholipids at acidic pH and to oxidized phospholipds at cytosolic pH. Plays a role in cell protection against oxidative stress by detoxifying peroxides and in phospholipid homeostasis. |
| Q9Y6N5 | Sulfide:quinone oxidoreductase | 3 | 0.47 | 0.00 | Mitochondrion |  | Catalyzes the oxidation of hydrogen sulfide with the help of a quinone, such as ubiquinone-10, giving rise to thiosulfate and ultimately to sulfane (molecular sulfur) atoms. Requires an additional electron acceptor; can use sulfite, sulfide or cyanide (in vitro). It is believed the in vivo electron acceptor is glutathione. |
| P20337 | Ras-related protein Rab-3B | 4 | 0.46 | 0.00 | Cell membrane, lipid-anchor, Golgi apparatus |  | Protein transport. Probably involved in vesicular traffic. |
| Q15746 | Myosin light chain kinase | 3 | 0.44 | 0.03 | Cytoplasm, lamellipodium, stress fiber | Aortic aneurysm, familial thoracic 7 (AAT7) | Calcium/calmodulin-dependent myosin light chain kinase implicated in smooth muscle contraction via phosphorylation of myosin light chains (MLC). In the nervous system it has been shown to control the growth initiation of astrocytic processes in culture and to participate in transmitter release at synapses formed between cultured sympathetic ganglion cells. Critical participant in signaling sequences that result in fibroblast apoptosis. May regulate optic nerve head astrocyte migration. |
| P07942 | Laminin subunit beta-1 | 2 | 0.44 | 0.00 | basement membrane | Lissencephaly 5 (LIS5) | Binding to cells via a high affinity receptor, laminin is thought to mediate the attachment, migration and organization of cells into tissues during embryonic development by interacting with other extracellular matrix components. Involved in the organization of the laminar architecture of cerebral cortex. It is probably required for the integrity of the basement membrane/glia limitans that serves as an anchor point for the endfeet of radial glial cells and as a physical barrier to migrating neurons. Radial glial cells play a central role in cerebral cortical development, where they act both as the proliferative unit of the cerebral cortex and a scaffold for neurons migrating toward the pial surface. |
| P48681 | Nestin | 4 | 0.41 | 0.00 |  |  | Required for brain and eye development. Promotes the disassembly of phosphorylated vimentin intermediate filaments (IF) during mitosis and may play a role in the trafficking and distribution of IF proteins and other cellular factors to daughter cells during progenitor cell division. Required for survival, renewal and mitogen-stimulated proliferation of neural progenitor cells. |
| P05204 | Non-histone chromosomal protein | 22 | 0.40 | 0.00 | Nucleus, cytoplasm |  | Binds to the inner side of the nucleosomal DNA thus altering the interaction between the DNA and the histone octamer. May be involved in the process which maintains transcribable genes in a unique chromatin conformation. |
| Q14203 | Dynactin subunit 1 | 3 | 0.26 | 0.05 | Cytoplasm, cytoskeleton, centrosome, spindle, nucleus envelope | Neuronopathy, distal hereditary motor, 7B (HMN7B)/Amyotrophic lateral sclerosis (ALS)/Perry syndrome (PERRYS) | Plays a key role in dynein-mediated retrograde transport of vesicles and organelles along microtubules by recruiting and tethering dynein to microtubules. Binds to both dynein and microtubules providing a link between specific cargos, microtubules and dynein. Essential for targeting dynein to microtubule plus ends, recruiting dynein to membranous cargos and enhancing dynein processivity (the ability to move along a microtubule for a long distance without falling off the track). Can also act as a brake to slow the dynein motor during motility along the microtubule. Can regulate microtubule stability by promoting microtubule formation, nucleation and polymerization and by inhibiting microtubule catastrophe in neurons. Inhibits microtubule catastrophe by binding both to microtubules and to tubulin, leading to enhanced microtubule stability along the axon. Plays a role in metaphase spindle orientation. Plays a role in centriole cohesion and subdistal appendage organization and function. Its recruitment to the centriole in a KIF3A-dependent manner is essential for the maintenance of centriole cohesion and the formation of subdistal appendage. Also required for microtubule anchoring at the mother centriole. Plays a role in primary cilia formation. |
| P04083 | Annexin A1 | 6 | 0.25 | 0.00 | Nucleus, cytoplasm, cell membrane, secreted |  | Plays important roles in the innate immune response as effector of glucocorticoid-mediated responses and regulator of the inflammatory process. Has anti-inflammatory activity. Plays a role in glucocorticoid-mediated down-regulation of the early phase of the inflammatory response. Promotes resolution of inflammation and wound healing. Functions at least in part by activating the formyl peptide receptors and downstream signaling cascades. Promotes chemotaxis of granulocytes and monocytes via activation of the formyl peptide receptors. Contributes to the adaptive immune response by enhancing signaling cascades that are triggered by T-cell activation, regulates differentiation and proliferation of activated T-cells. Promotes the differentiation of T-cells into Th1 cells and negatively regulates differentiation into Th2 cells. Has no effect on unstimulated T cells. Promotes rearrangement of the actin cytoskeleton, cell polarization and cell migration. Negatively regulates hormone exocytosis via activation of the formyl peptide receptors and reorganization of the actin cytoskeleton. Has high affinity for Ca2+ and can bind up to eight Ca2+ ions. Displays Ca2+-dependent binding to phospholipid membranes. Plays a role in the formation of phagocytic cups and phagosomes. Plays a role in phagocytosis by mediating the Ca2+-dependent interaction between phagosomes and the actin cytoskeleton. |
| Q9UMS6 | Synaptopodin-2 | 9 | 0.24 | 0.00 | Nucleus, cytoplasm, cytoskeleton |  | Has an actin-binding and actin-bundling activity. Can induce the formation of F-actin networks in an isoform-specific manner. At the sarcomeric Z lines is proposed to act as adapter protein that links nascent myofibers to the sarcolemma via ZYX and may play a role in early assembly and stabilization of the Z lines. Involved in autophagosome formation. May play a role in chaperone-assisted selective autophagy (CASA) involved in Z lines maintenance in striated muscle under mechanical tension; may link the client-processing CASA chaperone machinery to a membrane-tethering and fusion complex providing autophagosome membranes. Involved in regulation of cell migration. May be a tumor suppressor. |
| Q9BZF9 | Uveal autoantigen with coiled-coil domains and ankyrin repeats | 2 | 0.21 | 0.02 | Nucleus, cytoplasm, cytoskeleton |  | Regulates APAF1 expression and plays an important role in the regulation of stress-induced apoptosis. Promotes apoptosis by regulating three pathways, apoptosome up-regulation, LGALS3/galectin-3 down-regulation and NF-kappa-B inactivation. Regulates the redistribution of APAF1 into the nucleus after proapoptotic stress. Down-regulates the expression of LGALS3 by inhibiting NFKB1 |
| P45880 | Voltage-dependent anion-selective channel protein 2 | 3 | 0.20 | 0.01 | Mitochondrion outer membrane |  | Forms a channel through the mitochondrial outer membrane that allows diffusion of small hydrophilic molecules. The channel adopts an open conformation at low or zero membrane potential and a closed conformation at potentials above 30-40 mV. The open state has a weak anion selectivity whereas the closed state is cation-selective. |
