# Supplementary material for: Protein signature of human skin fibroblasts allows the study of the molecular etiology of rare neurological diseases
Source: Orphanet J Rare Dis. 2021 Feb 9;16:73. doi: 10.1186/s13023-020-01669-1 (PMC7874489; doi:10.1186/s13023-020-01669-1)
Supplement: Supplementary file 5 — Additional file 5. Findings of Coherent anti-Stokes Raman scattering (CARS) and second harmonic generation (SHG) measurements on patient-derived muscle biopsy specimen. [file 13023_2020_1669_MOESM5_ESM.docx]

Hentschel et al., 2020; OJRD: Fibroblasts to study rare neurological diseases

# **Supplemental document 1**

***Coherent anti-Stokes Raman scattering and Second Harmonic Generation spectroscopy***

CARS is a nonlinear variant of Raman spectroscopy. CARS microscopy provides inherently label-free biochemical information about the sample. We use this type of microscopy to investigate the spatially resolved distribution of lipid (e.g. 2845 cm^-1^, 2889 cm^-1^) (1) and protein distribution (e.g. 2932 cm^-1^) (2, 3) within muscle biopsies.

Materials and Methods

CARS measurements were performed on a modified Leica TCS SP 8 CARS microscope with an APE picoEmerald as laser source. The 1064 nm output of the picoEmerald was used as Stokes, the OPO provided both pump and probe. Before measuring spectra, single images were first taken with the OPO settings at 811/814/817 nm to get a first overview of the sample section. This combination results in a CARS signal at 655/659/663 nm and corresponds to a wavenumber of 2932/2887/2841 cm^−1^. Both lasers were fixed at a power of 900 mW at the picoEmerald output port. Further subsequent laser attenuation in the Leica systems was to 25 %. The laser beams were focused onto the sample using a Leica HCL IRAPO 40×/1.1 water objective. The resulting CARS signal was collected via the same objective in EPI (backward) direction and subsequently detected in the spectral regime of 560–750 nm via a photo multiplier tube (PMT) detector. The Second Harmonic Generation and Two-Photon fluorescence signals were collected at the same time (380–560 nm). Furthermore, all signals were also measured in a forward direction via a NA 0.55 condenser and the same filter/detector arrangement as in the EPI direction.

For the recording of spectra, the OPO signal was tuned in the range of 804-826.4 nm in steps of 0.7 nm, corresponding to 2700-3040 cm^-1^ and a resolution of roughly 10 cm^-1^. Thus, 33 images were obtained at the respective wavelengths and combined to a hypercube containing a spectrum for each point.

The Second Harmonic Generation (SHG) results from the interaction of two photons with molecules. The emission has the double frequency of the excitation. This effect can only occur in structures without inversion symmetry (4). In biological such structures are often called biophotonic crystals. The generation of an SHG signal in animal tissue is dominated by collagen (5, 6). Here, the aforementioned lasers were used to generate SHG signals.

The samples were stored on slides at -80 °C. Before the measurements in the CARS/SHG system, the samples were first thoroughly dried under a constant stream of dry air at room temperature.

***Statistical evaluation of muscle fibre calibres***

The Leica software LAS X was used to manually measure the length and width of fully imaged muscle fibres from the CARS images. The muscle fibre calibre was averaged from the two lengths. For muscle fibres in longitudinal section, the width of the fibre was determined as the calibre. For this purpose, a total of 10 images of the patient were analyzed.

***Acquisition of the spectra of the feature findings***

The spectral CARS/SHG measurements were manually screened for conspicuous features. These were grouped according to their appearance (see Fig. 4). The CARS spectra in each group were first normalized and then averaged. For comparison, inconspicuous areas neighboring the features were handled alike. A total of 78 spectra were used for the spectra shown in figure 4: A: 3, B: 6, C: 5, D: 4, F: 3, I: 4, J: 4, K: 5, L: 4, M: 4, O: 3, BG: 33.

References

1. Takamori M. Synaptic Homeostasis and Its Immunological Disturbance in Neuromuscular Junction Disorders. Int J Mol Sci. 2017;18(4).

2. Gonzalez Coraspe JA, Weis J, Anderson ME, Munchberg U, Lorenz K, Buchkremer S, et al. Biochemical and pathological changes result from mutated Caveolin-3 in muscle. Skelet Muscle. 2018;8(1):28.

3. Cheng JX, Xie XS. Vibrational spectroscopic imaging of living systems: An emerging platform for biology and medicine. Science. 2015;350(6264):aaa8870.

4. Schürmann S. Second Harmonic Generation Mikroskopie in biomedizinischen Anwendungen (Second Harmonic Generation Microscopy in Biomedical Applications): University Heidelberg (Germany); 2010.

5. Plotnikov SV, Millard AC, Campagnola PJ, Mohler WA. Characterization of the Myosin-Based Source for Second-Harmonic Generation from Muscle Sarcomeres. Biophys J. 2006;90(2):693-703.

6. Zipfel WR, Williams RM, Christie R, Nikitin AY, Hyman BT, Webb WW. Live tissue intrinsic emission microscopy using multiphoton-excited native fluorescence and second harmonic generation. Proc Natl Acad Sci USA. 2003;100(12):7075.
